# Unveiling the perfect workout: exercise modalities and dosages to ameliorate adipokine dysregulation in individuals with overweight and obesity: a systematic review with pairwise, network, and dose–response meta-analyses

**DOI:** 10.3389/fnut.2025.1653449

**Published:** 2025-08-12

**Authors:** Hai Wang, Hao Wang, Enyan Zhan, Xiaodong Liu

**Affiliations:** Capital University of Physical Education and Sports, Beijing, China

**Keywords:** exercise, adipokine, adiponectin, leptin, overweight, obesity, meta-analysis

## Abstract

**Background:**

Obesity is commonly associated with dysregulation of adipokines, particularly characterized by elevated leptin levels and reduced adiponectin levels. These abnormalities are closely linked to an increased risk of developing type 2 diabetes mellitus (T2DM), cardiovascular diseases, and certain cancers.

**Objective:**

To systematically evaluate the effects of different exercise modalities—AE, RT, COM, HIIT—and their dosages on the regulation of adipokines (leptin and adiponectin) in individuals with overweight and obesity.

**Methods:**

A comprehensive literature search was conducted across Cochrane, Embase, PubMed, Web of Science, and EBSCO databases for randomized controlled trials (RCTs) published between 2000 and January 2025. A random-effects Bayesian model was applied to perform pairwise meta-analysis, network meta-analysis, and dose–response meta-analysis to compare the effects of various exercise interventions and their respective doses. Additionally, meta-regression analysis was conducted to explore potential moderating effects of age, sex, body mass index (BMI), and body fat percentage on the intervention outcomes.

**Results:**

A total of 61 RCTs involving 3,069 participants were included. The network meta-analysis showed that all exercise interventions significantly increased adiponectin levels, with HIIT yielding the most pronounced effect (SMD = 0.85, 95% CrI: 0.24–1.45; SUCRA = 68%), followed by RT, AE, and COM. For leptin levels, COM was the most effective intervention (SMD = −0.99, 95% CrI: −1.48 to −0.51; SUCRA = 84%), followed by AE and HIIT, while RT did not demonstrate a statistically significant effect. Dose–response analysis indicated an inverted U-shaped relationship between exercise and improvements in adiponectin, with the optimal effective dose around 880 MET-min/week. In contrast, a negative linear relationship was observed between exercise and leptin, with notable improvements starting at approximately 770 MET-min/week. The dose–response relationships varied by exercise type. Meta-regression further revealed that age and BMI were positively associated with improvements in adiponectin, while BMI and body fat percentage were positively correlated with improvements in leptin. However, age was negatively associated with changes in leptin. No significant moderating effect of sex was observed on changes in adipokine levels.

**Conclusion:**

This study provides moderate-quality evidence supporting the effectiveness of HIIT, COM, and AE in improving adipokine profiles among individuals with overweight and obesity, although the effects differ by exercise modality. The well-defined dose–response relationships underscore the importance of individualized exercise prescriptions, with moderate to high weekly doses (approximately 800–1,300 MET-min/week) recommended to optimize endocrine and metabolic health. Future research should further investigate inter-individual variability in response to exercise interventions and their long-term clinical benefits.

**Systematic review registration:**

CRD420251010709, https://www.crd.york.ac.uk/PROSPERO/.

## 1 Introduction

Global obesity rates are rising at an unprecedented pace. According to the World Health Organization (WHO), the global prevalence of obesity has more than doubled since 1980, with over 25% of adults currently classified as having obesity (1). This alarming trend not only poses a major public health challenge but also significantly increases the incidence and mortality rates associated with type 2 diabetes, cardiovascular diseases, and certain types of cancer (2–4). The high prevalence of obesity and its related complications necessitate an in-depth exploration of its underlying mechanisms to identify effective intervention strategies.

Adipose tissue, beyond its role in energy storage, also functions as an endocrine organ that secretes various bioactive substances, among which leptin and adiponectin are the most representative. Leptin is predominantly secreted by white adipose tissue, with its levels positively correlated with fat mass. It plays a central role in appetite regulation and energy balance (5, 6). However, individuals with obesity often exhibit markedly elevated leptin levels accompanied by a phenomenon known as “leptin resistance,” wherein high leptin concentrations fail to effectively suppress appetite or promote energy expenditure (7). In contrast, adiponectin—first identified in 1995—enhances insulin sensitivity, possesses anti-inflammatory properties, and inhibits angiogenesis (8–10). Of concern is that adiponectin levels are typically reduced in individuals with obesity, a dysregulation that may exacerbate insulin resistance and inflammatory responses, thereby further contributing to cardiovascular disease and metabolic syndrome (11, 12).

In recent years, numerous studies have indicated that lifestyle modifications can positively regulate serum leptin and adiponectin levels. Measures such as improved sleep duration, smoking cessation, dietary control, and increased fruit and vegetable intake have been shown to lower leptin levels while elevating adiponectin concentrations (13–16). Among these, exercise—defined as planned, structured, and repetitive physical activity—has emerged as a key intervention for improving adipokine dysregulation due to its direct impact on energy expenditure and body fat composition (16). Moreover, exercise can indirectly modulate leptin and adiponectin secretion by improving insulin sensitivity, reducing systemic inflammation, and restoring endothelial function (17). For instance, several randomized controlled trials (RCTs) have demonstrated that long-term aerobic exercise significantly increases adiponectin levels while decreasing leptin concentrations (18, 19). However, some studies have reported no significant changes, suggesting that the effects of exercise on adipokines may vary depending on exercise modality, intensity, duration, and the baseline metabolic status of participants (20, 21).

The mechanisms underlying the regulation of adipokines by exercise are multifaceted. Firstly, exercise significantly reduces body fat, thereby decreasing both the number and size of adipocytes, which in turn lowers leptin secretion (22). Secondly, exercise improves insulin resistance, enabling insulin to resume its regulatory role in adiponectin secretion, thereby increasing adiponectin levels (23). In addition, the anti-inflammatory effects of exercise—through the suppression of pro-inflammatory cytokines such as TNF-*α* and CRP—help alleviate inflammation-mediated adipokine dysregulation (24). These interacting mechanisms collectively contribute to the beneficial effects of exercise on endocrine disturbances in obesity. Nonetheless, existing RCT findings on the relationship between exercise interventions and adipokine changes remain inconsistent, potentially due to heterogeneity in intervention protocols, sample characteristics, and study methodologies.

Given these discrepancies, there is a pressing need for a systematic review and quantitative synthesis to clarify the effects of different exercise modalities and their “doses” on leptin and adiponectin regulation. Network meta-analysis (NMA), as an advanced methodological approach, allows for the integration of direct and indirect comparisons, offering a unified assessment of multiple interventions. Dose–response meta-analysis further facilitates the exploration of quantitative relationships between factors such as exercise intensity, frequency, and duration, and changes in adipokine levels. These approaches are crucial for elucidating the mechanisms through which exercise modulates obesity-related endocrine abnormalities.

Accordingly, the present study aims to systematically evaluate the effects of different exercise modalities and dosages on serum leptin and adiponectin levels. Using pairwise meta-analysis, network meta-analysis, and dose–response meta-analysis, we comprehensively compared the efficacy of various intervention protocols. By synthesizing data from existing RCTs, we sought to address the following key questions: First, do different exercise modalities (e.g., aerobic exercise, resistance training, high-intensity interval training, and combined training) exert differential effects on leptin and adiponectin regulation? Second, is there a dose–response relationship between exercise and improvements in adipokine levels? Third, which exercise interventions provide the most favorable endocrine regulatory benefits for individuals with obesity, thereby offering evidence-based guidance for the development of personalized exercise prescriptions in clinical settings.

## 2 Methods

### 2.1 Registration

The protocol of this systematic review and meta-analysis has been registered and approved in the International Prospective Register of Systematic Reviews (PROSPERO; registration number: CRD420251010709). The study has been reported in accordance with the PRISMA-NMA checklist (25).

### 2.2 Search strategy and study selection

This study conducted a comprehensive search of five major databases—Cochrane, Embase, PubMed, Web of Science, and EBSCO—for randomized controlled trials (RCTs) investigating the effects of exercise on adipokines (adiponectin and leptin). The search was limited to articles published in English between 2000 and January 2025. In addition, the reference lists of previously published systematic reviews and meta-analyses were manually screened to identify additional relevant RCTs. The search strategy included the use of subject headings and keywords such as “exercise,” “overweight,” “obesity,” “individuals with overweight and obesity,” “adipokines,” “adiponectin,” “leptin,” and “RCT.” A detailed search strategy is provided in Appendix 1.

Two researchers (Cx-W and HW) independently screened the titles and abstracts, followed by full-text screening based on predefined inclusion criteria. Discrepancies were resolved by consulting a third researcher (Xd-L). Endnote X9 (Thompson ISI Research Soft, Philadelphia, PA, USA) was used to manage and organize the retrieved records.

### 2.3 Eligibility criteria

Studies were included if they met the following criteria.

#### 2.3.1 Study design

The study must be a randomized controlled trial (RCT) published in a peer-reviewed English-language journal.

#### 2.3.2 Intervention

At least one intervention group must have undergone a structured exercise training program lasting no less than 8 weeks, without the concurrent use of supplements or dietary restrictions during the intervention period. Details regarding the classification of exercise modalities are provided in Appendix 2. The control group was required to maintain a non-exercise lifestyle and preserve habitual living behaviors. Additionally, studies that included two or more exercise intervention groups without a separate control group were also eligible for inclusion to enable direct comparisons between exercise modalities.

#### 2.3.3 Participant criteria

Participants were required to be individuals with overweight and obesity, defined according to the following body mass index (BMI) thresholds:

For European populations: overweight defined as BMI > 25 kg/m^2^ and obesity as BMI ≥ 30 kg/m^2^.

For Asian populations: overweight defined as BMI ≥ 24 kg/m^2^ and obesity as BMI ≥ 28 kg/m^2^.

If BMI data were not reported, body fat percentage (%BF) was used as an alternative criterion, with thresholds set at ≥ 30% for women and ≥ 25% for men. No age restrictions were imposed on study participants.

#### 2.3.4 Outcomes

Studies were required to assess changes in circulating (plasma or serum) levels of adipokines—specifically leptin and adiponectin—before and after the exercise intervention.

Studies were excluded if they met any of the following criteria:

1. Duplicate publications, letters to the editor, dissertations, studies investigating only the acute effects of a single exercise session, or animal studies.
2. Non-original research articles, such as reviews, conference abstracts, or case reports.
3. Studies combining exercise with other interventions or lifestyle modifications.
4. Studies lacking sufficient detail on participant characteristics or intervention protocols.
5. Studies for which full texts or relevant data could not be obtained, even after contacting the authors.

### 2.4 Data extraction

Two researchers (Cx-W and HW) independently extracted data from studies that met the inclusion criteria. The following information was extracted from each eligible record: (1) first author and year of publication; (2) country; (3) participant characteristics, including sample size in both intervention and control groups, sex distribution, mean age, body mass index (BMI), body fat percentage (%BF), and comorbidities; (4) detailed information on the intervention, including exercise type, frequency, duration, intervention period, intensity, and exercise modality; and (5) primary outcome measures—adipokines (adiponectin and leptin). Any discrepancies in data extraction were resolved through consultation with a third reviewer (Xd-L). In cases of missing data, the corresponding authors of the relevant studies were contacted up to three times over a three-week period to obtain the required information.

### 2.5 Risk of bias

Two independent researchers (Cx-W and HW) assessed the risk of bias of the included studies using the second version of the Cochrane Risk of Bias tool (26). Any disagreements were resolved through consultation with a third experienced researcher (Xd-L).

### 2.6 Quality of evidence evaluation

Two independent reviewers assessed the quality of evidence using the Grading of Recommendations Assessment, Development, and Evaluation (GRADE) (27)approach. The GRADE framework evaluates evidence based on five key factors: study limitations, consistency, directness, precision, and publication bias. Based on these factors, the quality of evidence is classified as “high,” “moderate,” “low,” or “very low.”

### 2.7 Data coding and management

In this study, exercise intensity was quantified using metabolic equivalent minutes per week (MET-min/week). A metabolic equivalent (MET) represents the energy cost of physical activity, expressed as a multiple of the resting metabolic rate. By multiplying the MET value of a given activity by its duration (in minutes) and frequency per week, the total energy expenditure of a specific exercise regimen can be comprehensively calculated. MET-min/week is a standardized unit widely used in the field of exercise science, as it incorporates both the intensity and duration of physical activity, thereby allowing for direct comparisons across different exercise modalities—even when they vary in type, duration, or frequency. A substantial body of research has validated MET-min as a reliable metric for evaluating the dose of physical activity in relation to various health outcomes. For instance, the *2024 Compendium of Physical Activities* (28) provides robust evidence supporting the use of MET values to quantify energy expenditure. Additionally, both the *World Health Organization’s Guidelines on Physical Activity and Sedentary Behavior* (29) and the *American College of Sports Medicine’s recommendations* (30) endorse the use of MET-min/week to characterize physical activity, emphasizing its clinical relevance and enhancing the generalizability of this study’s findings. Using MET-min/week enables more precise quantification of exercise dosage and improves the interpretability of the dose–response relationship between exercise and sleep quality.

In practical application, the intensity of each specific exercise was coded based on the *2024 Compendium of Physical Activities* (28), which includes 821 codes for specific activities covering nearly all forms of physical activity. Exercise frequency was defined as the total number of exercise sessions per week, including multiple sessions within a single day. If the duration of a single exercise session was not explicitly reported, the average duration across all relevant studies involving the same intervention was used. In cases where exercise duration gradually increased over several weeks, the average of the total intervention period was calculated. Finally, to facilitate network connectivity and dose–response network analysis, the weekly MET-min values were categorized into six levels: 0 (control group), 300, 600, 900, 1,200, and 1,500 MET-min/week (31).

### 2.8 Measures of treatment effect

This meta-analysis evaluated treatment effects using the mean difference (MD) and standard deviation (SD) of changes from baseline. If SDs were not directly reported, they were calculated from standard errors, 95% confidence intervals, *p*-values, or *t*-statistics (32). In addition, a correlation coefficient of 0.8 was assumed when calculating the SD of change scores before and after the intervention. This assumption was based on widely accepted evidence in the literature indicating moderate measurement repeatability. The choice of this value aimed to balance the potential variability between pre- and post-intervention measurements while ensuring the conservativeness and reliability of the results (32).

### 2.9 Statistical analysis

#### 2.9.1 Data transformation

To address the limitation that the GEMTC package in R supports only mean difference (MD) calculations and does not directly allow for standardized mean difference (SMD) computations—and given the inconsistency in measurement units across studies—this study adopted the following data processing procedures.

##### 2.9.1.1 SMD calculation

All original data were imported into STATA 16. Based on recommended methods in the literature (Hedges’s method), the SMD and its corresponding variance were calculated for each study (33).

##### 2.9.1.2 Data export and conversion

The SMD and variance values calculated in STATA were exported in CSV format, ensuring compatibility with the data format required for subsequent use in R.

##### 2.9.1.3 Network meta-analysis in R

The pre-calculated SMDs and their variances were imported into the R environment using a relative data format (34), and Bayesian network meta-analysis was conducted using the GEMTC package. Although GEMTC natively supports only MD calculations, this preprocessing approach enabled the implementation of network meta-analysis based on SMD values.

#### 2.9.2 Pairwise meta-analyses and publication bias

A random-effects model was employed for the pairwise meta-analysis in this study. Effect sizes were synthesized based on the standardized mean difference (SMD) of treatment effects, along with the corresponding 95% credible intervals (CrIs). Effect sizes were classified according to the Cochrane handbook as large (SMD > 0.70), moderate (SMD: 0.40—0.70), or small (SMD < 0.40) (32). Heterogeneity across studies was assessed using the *I*^2^ statistic, with an *I*^2^ value greater than 50% indicating substantial heterogeneity between studies (35). Additionally, potential publication bias was evaluated using the corrected funnel plot asymmetry test and Egger’s test (36).

#### 2.9.3 Network meta-analysis

Bayesian network meta-analysis was conducted using the “gemtc” package in R (37, 38) and the web-based tool Metainsight (39). This approach is based on the Bayesian statistical framework and utilizes the Markov Chain Monte Carlo (MCMC) algorithm to estimate model parameters (37). During the analysis, four parallel chains were run simultaneously, each initialized with different random starting values, and a total of 50,000 iterations were performed. To minimize the influence of initial values, the first 5,000 iterations were discarded after reaching the target distribution (40). Model convergence was assessed using the Brooks–Gelman–Rubin diagnostic statistic. Given the anticipated heterogeneity across studies, a random-effects model was applied (41).

Global inconsistency was evaluated by comparing the model fit, deviance information criterion (DIC), and variance parameters between the consistency model and the unrelated mean effects model. Node-splitting analysis was further employed to assess local inconsistency between direct and indirect comparisons within the network; a *p*-value < 0.05 was considered indicative of inconsistency (42). In addition, a network plot was constructed to visually display the direct and indirect comparisons among different exercise modalities. The surface under the cumulative ranking curve (SUCRA) was used to rank the efficacy of each intervention, with values ranging from 0 to 100%, where higher scores indicate greater effectiveness.

#### 2.9.4 Network meta-analysis of dose–response

This study employed a random-effects Bayesian model-based network meta-analysis (MBNMA) to investigate the dose–response relationship between exercise and adipokines (adiponectin and leptin) (43). During the analysis, no evidence was found to suggest violations of the key assumptions of MBNMA, including network transitivity (31), data consistency (44), and network connectivity (45) (see Appendix 13 for details). The standardized mean difference (SMD) was used as the effect size metric, and 95% credible intervals (CrIs) were used to evaluate the precision of the estimates.

To compare the goodness-of-fit of different dose–response function models, we assessed the deviance information criterion (DIC), between-study standard deviation, model parameters, and residual deviance for the Emax model, restricted cubic spline, quadratic function, and non-parametric models (46). Results indicated that the quadratic function model outperformed the others across all evaluation metrics and was therefore selected to characterize the nonlinear dose–response relationship (see Appendix 14). In the nonlinear analysis, the random-effects quadratic model not only demonstrated good convergence but also provided a more realistic representation of the underlying biological patterns under time- and dose-dependent conditions compared to linear models (47).

An effect was considered statistically significant if the 95% CrI for the SMD did not include zero. All analyses were conducted in R software (version 4.3.1), with the “MBNMAdose” package used for MBNMA and dose–response modeling, and the “ggplot2” package used to generate dose–response curves for data visualization.

## 3 Results

### 3.1 Literature selection

**Figure 1:**
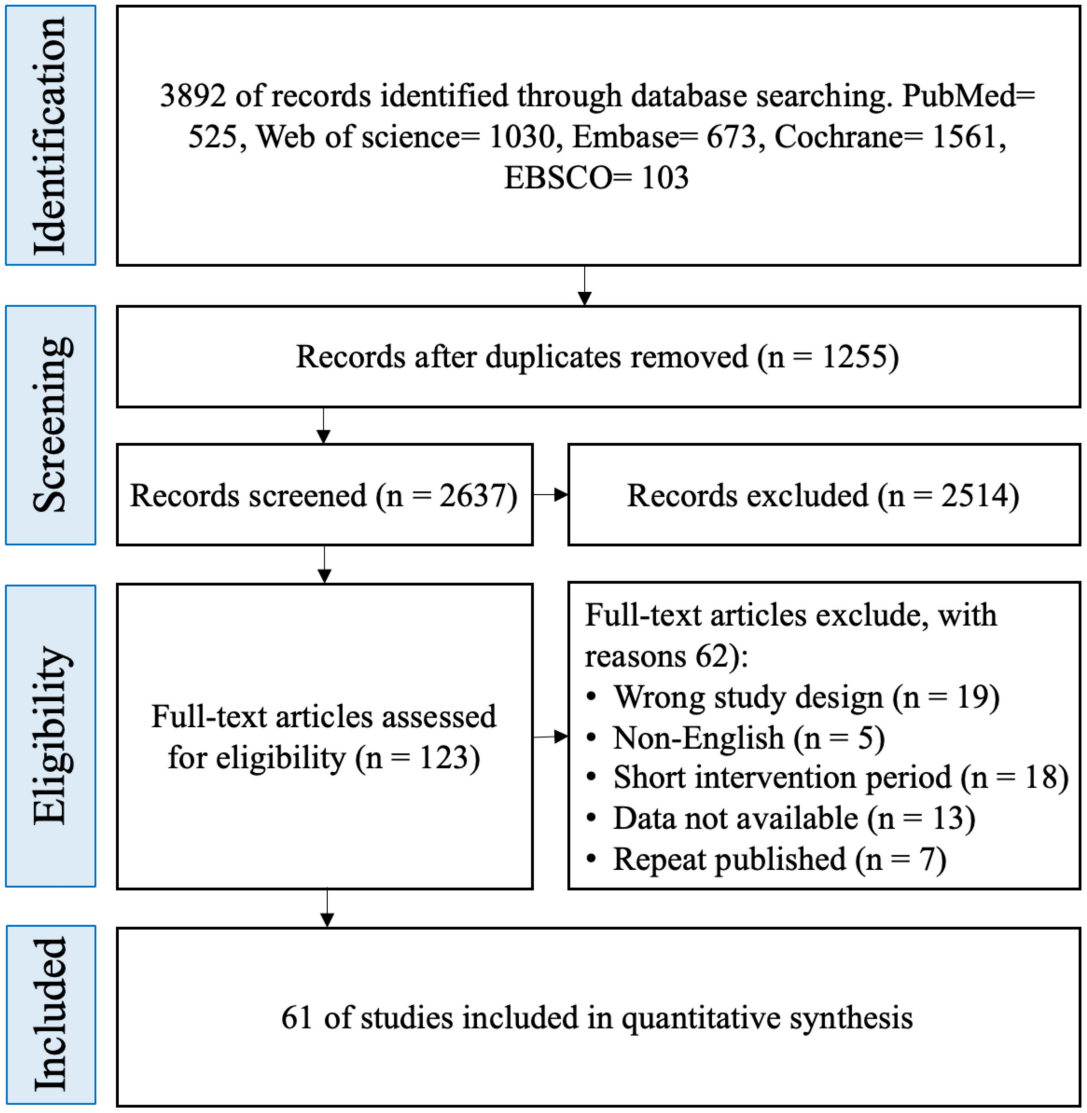
Literature screening process and results.

A total of 3,892 potentially eligible randomized controlled trials (RCTs) were identified through systematic searches of five major databases: Cochrane, Embase, PubMed, Web of Science, and EBSCO. After removing 1,255 duplicate records, 2,637 articles remained. Title and abstract screening led to the exclusion of 2,514 studies. Full-text review was subsequently conducted for the remaining 123 articles, of which 62 were excluded based on the predefined eligibility criteria. Ultimately, 61 RCTs were included in the final analysis (Figure 1).

### 3.2 Characteristics of the included studies

The included studies were predominantly published between 2000 and 2025. In terms of participants, a total of 3,069 individuals were involved, of whom 1,712 were women (56%). Specifically, 23 studies included only female participants, 21 included only male participants, 16 included both sexes, and the remaining 5 studies did not report the sex or sex distribution of the participants. The average body mass index (BMI) of participants ranged from 18 kg/m^2^ to 43.91 kg/m^2^, and the average body fat percentage ranged from 22.9 to 51.05%.

Regarding the characteristics of the exercise interventions, four types of exercise modalities were included: AE, RT, COM, and HIIT. Among the intervention groups, 35 studies implemented AE, 18 implemented RT, 24 implemented COM, and 17 implemented HIIT. A total of 55 studies included a CON, in which participants either did not engage in any exercise or performed only light stretching or received health education. The duration of exercise interventions ranged from 8 to 52 weeks, with 8-week and 12-week durations being the most common.

In terms of adipokine outcomes, 49 studies reported data on adiponectin, while 41 studies reported data on leptin (The detailed characteristics of the included studies are presented in Appendix 3).

#### 3.2.1 Results of ROB assessment

Among the 61 studies included in this analysis, 9 were assessed as having a high risk of bias, 14 as having a moderate risk of bias, and 38 as having a low risk of bias. Detailed results of the risk of bias assessment are presented in Appendix 4.

### 3.3 GRADE assessment

Appendix 12 presents the GRADE assessment for each comparison and the SUCRA rankings for the effects of exercise on adipokines. Overall, the quality of evidence for most comparisons related to adiponectin and leptin was rated as moderate to low. The GRADE rating for the SUCRA rankings was moderate.

### 3.4 Pairwise meta-analyses and publication bias

**Figure 2:**
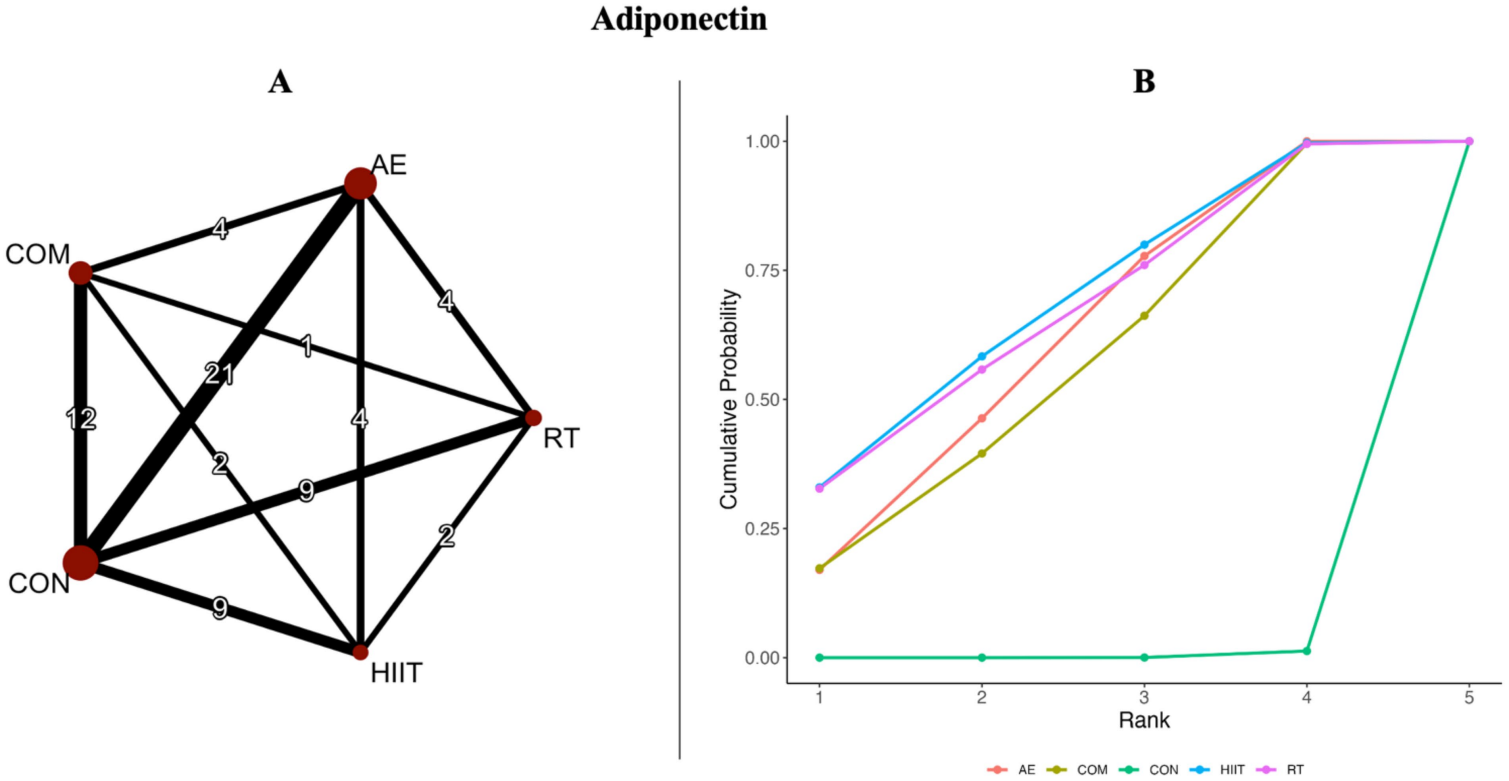
**(A)** Network diagrams depicting the direct and indirect comparisons for the network meta-analyses. **(B)** Bayesian ranking panel plots. The surface under the cumulative ranking curve (SUCRA) value is used to assess the relative effectiveness of different exercise interventions. Higher SUCRA values indicate a better exercise effect. AE, aerobic exercise; RT, resistance exercise; COM, AE combined RT; HIIT, high-intensity interval training; CON, control.

**Figure 3:**
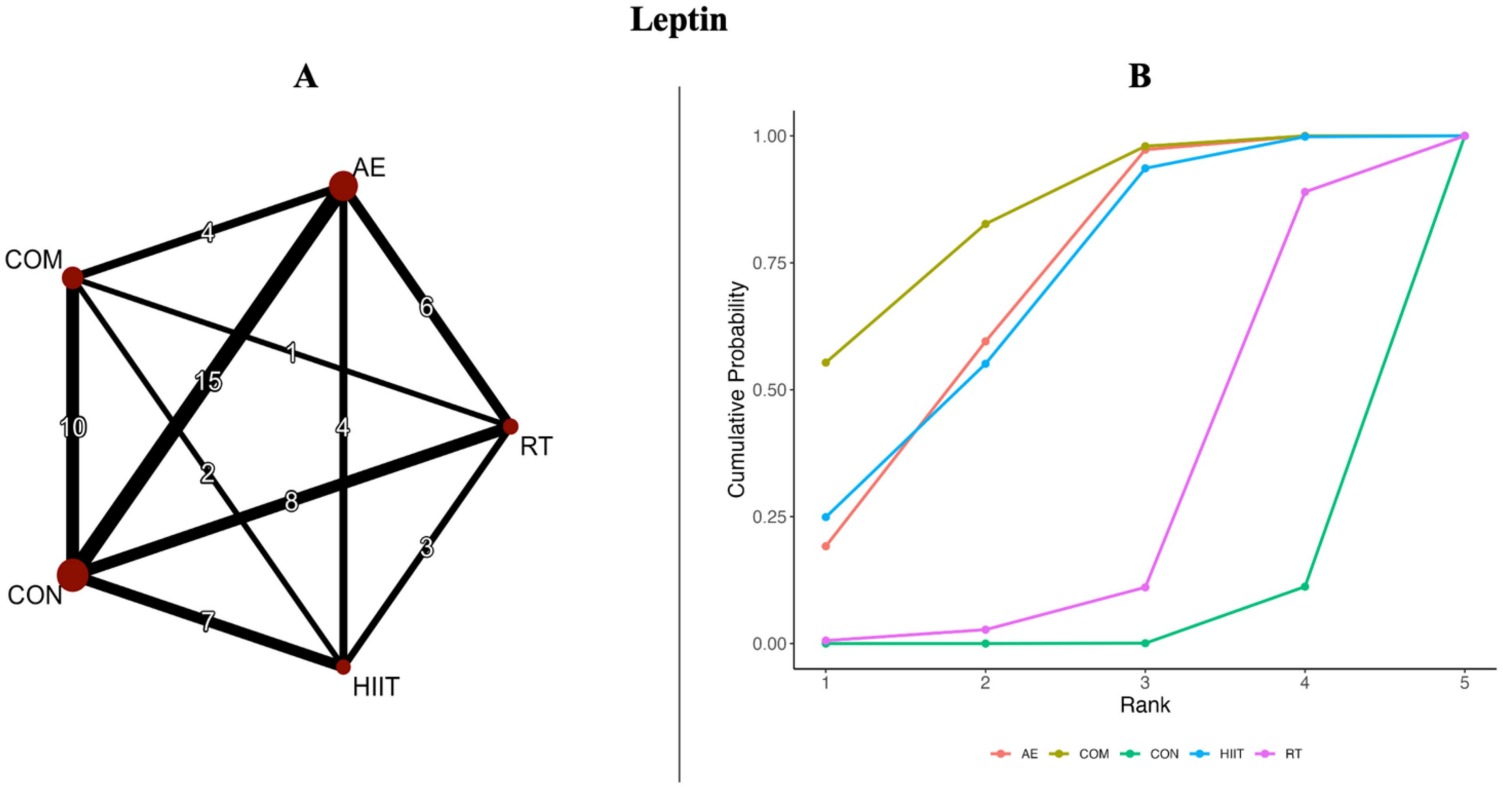
**(A)** Network diagrams depicting the direct and indirect comparisons for the network meta-analyses. **(B)** Bayesian ranking panel plots. The surface under the cumulative ranking curve (SUCRA) value is used to assess the relative effectiveness of different exercise interventions. Higher SUCRA values indicate a better exercise effect. AE, aerobic exercise; RT, resistance exercise; COM, AE combined RT; HIIT, high-intensity interval training, CON, control.

The results of the pairwise meta-analysis are illustrated in the orange areas of Figures 2, 3 and further detailed in Appendix 3.

**Figure 4:**
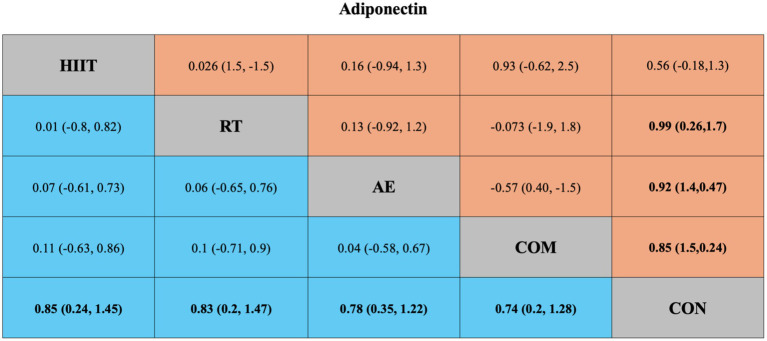
League table of direct and network comparisons of the effects of different exercise interventions on Adiponectin. The data shown in the table are mean differences and 95% credible intervals. For efficacy in post-exercise, a mean difference less than 0 favors the column-defining treatment. Exercises are reported in order of surface under the curve cumulative ranking. Results of the network meta-analysis are presented in the left lower half and results from the pairwise meta-analysis in the upper right half, if available. Cells in bold print indicate significant results. AE, aerobic exercise; RT, resistance exercise; COM, AE combined RT; HIIT, high-intensity interval training; CON, control.

For adiponectin, the pairwise meta-analysis included four direct comparisons. Compared with the control group, all intervention modalities significantly improved adiponectin levels except for HIIT (SMD = 0.56, 95% CrI [−0.18, 1.3], *I*^2^ = 75.73%) (Figure 4). However, substantial heterogeneity was observed across most comparisons (*I*^2^ > 70%) (Appendix 5; Table 2). In addition, visual inspection of the funnel plot and results from Egger’s test (*p* = 0.0445) suggested potential publication bias and small-study effects (Appendix 6; Figure 1).

**Table 2:** Results of meta-regression adiponectin.

Moderator	Study (*n*)	*β*	SE	*Z*	*p*	95% CI	R^2^
Age	44	0.019	0.009	2.18	0.03	(0.002, 0.04)	9%
Gender	31	−0.41	0.4	−1.04	0.3	(−1.19, 0.37)	0%
BMI	44	0.14	0.05	2.7	0.007	(0.04, 0.24)	14%
BF%	26	−0.006	0.03	−0.22	0.83	(−0.06, 0.05)	0%

**Figure 5:**
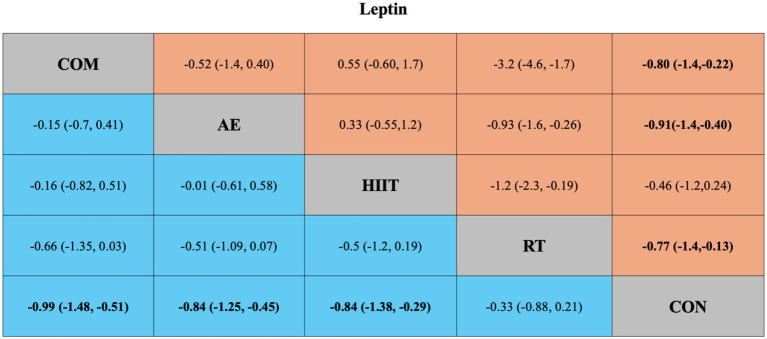
League table of direct and network comparisons of the effects of different exercise interventions on Leptin. The data shown in the table are mean differences and 95% credible intervals. For efficacy in post-exercise, a mean difference less than 0 favors the column-defining treatment. Exercises are reported in order of surface under the curve cumulative ranking. Results of the network meta-analysis are presented in the left lower half and results from the pairwise meta-analysis in the upper right half, if available. Cells in bold print indicate significant results. AE, aerobic exercise; RT, resistance exercise; COM, AE combined RT; HIIT, high-intensity interval training; CON, control.

For leptin, four pairwise comparisons were also included. Compared with the control group, all interventions significantly reduced leptin levels except for HIIT (SMD = −0.46, 95% CrI [−1.2, 0.24], *I*^2^ = 81.66%) (Figure 5). Again, high heterogeneity was observed across most comparisons (*I*^2^ > 70%) (Appendix 5; Table 3). The funnel plot and Egger’s test (*p* = 0.0351) indicated possible publication bias and small-study effects (Appendix 6; Figure 2).

**Table 3:** Results of meta-regression leptin.

Moderator	Study (*n*)	*β*	SE	*Z*	*p*	95% CI	R^2^
Age	35	0.011	0.005	2.32	0.020	(0.002, 0.02)	30%
Gender	33	−0.05	0.28	−0.18	0.86	(−0.60, 0.5)	0
BMI	29	−0.11	0.05	−2.04	0.04	(−0.21, −0.004)	11%
BF%	19	−0.05	0.02	−2.29	0.02	(−0.08, −0.006)	18%

*β*, The meta-regression coefficient, representing the estimated effect of the regressor on the effect size. SE, The standard error of the regression coefficient, indicating the uncertainty of the estimate. *p*, The *p*-value associated with the *Z* statistic, indicating the statistical significance of the regression coefficient. 95% CI, The 95% confidence interval for the regression coefficient. R^2^, The proportion of between-study heterogeneity explained by the covariate in the meta-regression model. BMI, Body mass index; BF%, Body fat percentage.

### 3.5 Network meta-analysis

Figures 2A, 3A present the network evidence maps for the effects of five intervention modalities—AE, RT, COM, HIIT, and CON—on adipokines (adiponectin and leptin) in individuals with overweight or obesity. In these figures, the size of the red circles corresponds to the sample size for each intervention. Black lines indicate direct comparisons between interventions, with the numbers on the lines representing the number of studies comparing the two interventions directly.

#### 3.5.1 Adiponectin

The network meta-analysis of adiponectin included 49 trials with a total of 2,365 participants, comparing four exercise interventions. The blue area in Figure 4 shows the results of the network meta-analysis, including standardized mean differences (SMDs) and 95% credible intervals (CrIs). Compared with the control group, all four exercise modalities significantly improved adiponectin levels in individuals with overweight or obesity: HIIT (SMD = 0.85, 95% CrI [0.24, 1.45], GRADE: Moderate), RT (SMD = 0.83, 95% CrI [0.20, 1.47], GRADE: Moderate), AE (SMD = 0.78, 95% CrI [0.35, 1.22], GRADE: Low), and COM (SMD = 0.74, 95% CrI [0.20, 1.28], GRADE: Low).

Additionally, the SUCRA rankings were used to assess the relative effectiveness of each intervention. HIIT ranked highest (SUCRA: 68%, GRADE: Moderate), followed by RT (SUCRA: 66%, GRADE: Moderate), AE (SUCRA: 60%, GRADE: Moderate), and COM (SUCRA: 56%, GRADE: Moderate) (Figure 2B; Table 1, Adiponectin section).

**Table 1:** Ranking of exercise interventions in order of effectiveness.

Adiponectin		Leptin	
Treatment	SUCRA	Treatment	SUCRA
HIIT	68%	COM	84%
RT	66%	AE	69%
AE	60%	HIIT	68%
COM	56%	RT	26%
CON	0.34%	CON	2.8%

AE, aerobic exercise; RT, resistance exercise; COM, AE combined RT; HIIT, high-intensity interval training; CON, control.

#### 3.5.2 Leptin

The network meta-analysis of leptin included 41 trials with a total of 2,000 participants, also comparing four exercise modalities. The blue area in Figure 5 shows the network meta-analysis results, including SMDs and 95% CrIs. Compared with the control group, all exercise interventions except RT significantly reduced leptin levels in individuals with overweight or obesity: COM (SMD = −0.99, 95% CrI [−1.48, −0.51], GRADE: Very Low), AE (SMD = −0.84, 95% CrI [−1.25, −0.45], GRADE: Moderate), HIIT (SMD = −0.84, 95% CrI [−1.38, −0.29], GRADE: Moderate), while RT showed no significant effect (SMD = 0.33, 95% CrI [−0.88, 0.21], GRADE: Low).

SUCRA rankings indicated that COM had the highest SUCRA value (SUCRA: 84%, GRADE: Moderate), followed by AE (SUCRA: 69%, GRADE: Moderate), HIIT (SUCRA: 68%, GRADE: Moderate), and RT (SUCRA: 26%, GRADE: Moderate) (Figure 3B; Table 2, Leptin section).

Appendix 7 reports the model fit statistics for the network meta-analyses of adiponectin and leptin. There was no substantial difference in model fit between the random-effects NMA model (DIC = 317.1) and the unrelated mean effects model (DIC = 321.5) for either adiponectin or leptin.

Appendix 10 presents the node-splitting results for adiponectin and leptin. For adiponectin, no significant inconsistency was detected between direct and indirect comparisons. For leptin, significant inconsistency was observed only in the comparisons between RT vs. COM and RT vs. CON.

Appendix 8 provides the MCMC convergence diagnostic plots for adiponectin and leptin. The shrinkage factors for all parameters approached 1 in the later iterations, indicating good model convergence.

Appendix 9 displays the MCMC sampling trajectories and posterior distributions for adiponectin and leptin. The sampling paths and posterior distributions of all parameters supported the reliability and convergence of the model.

### 3.6 Dose–response NMAs

#### 3.6.1 Adiponectin

**Figure 6:**
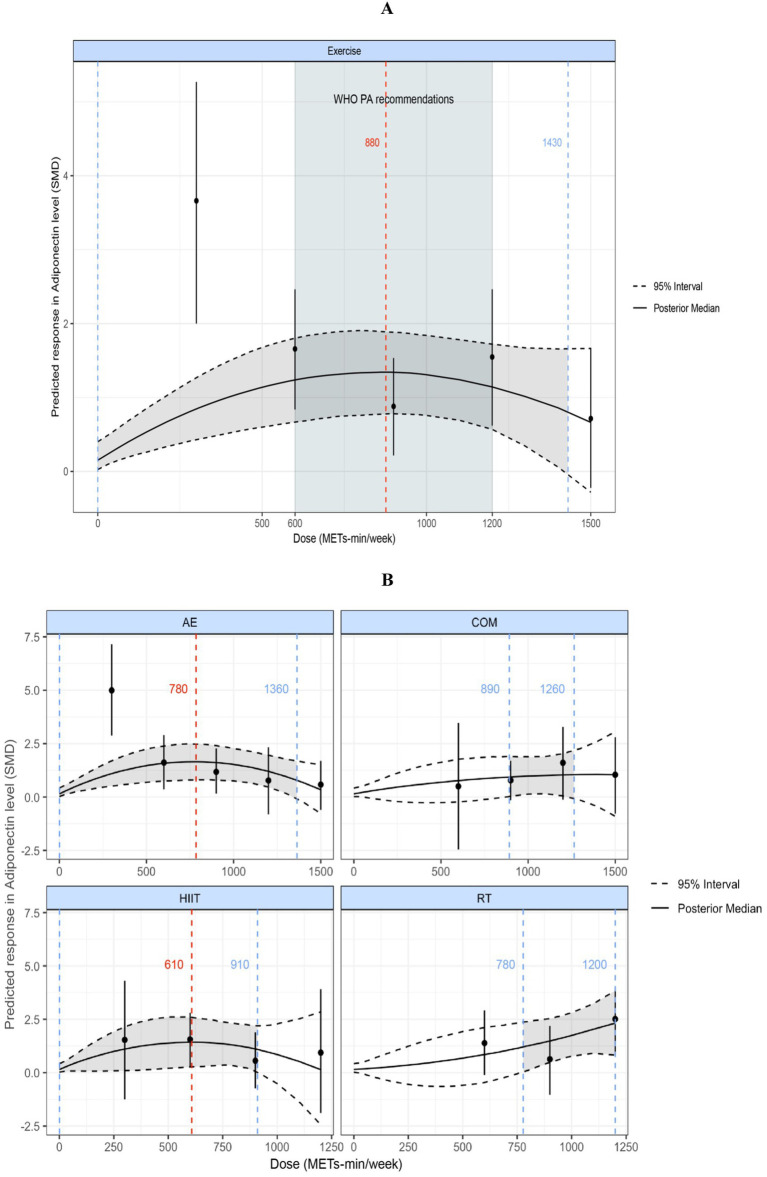
**(A)** Dose–response relationship between total weekly exercise volume and adiponectin levels in individuals with overweight or obesity. **(B)** Dose–response relationship between different types of weekly exercise and adiponectin levels in individuals with overweight or obesity. The blue dashed lines indicate the beginning and end of the significant 95% credible interval, and the blue numbers represent the corresponding dose values at these points (METs-min/week); the red dashed line and red numbers indicate the position and specific dose value at which the optimal dose–response occurs (METs-min/week). MET, metabolic equivalent of task; PA, physical activity; SMD, standardized mean difference; WHO, World Health Organization. AE, aerobic exercise; RT, resistance exercise; COM, AE combined RT; HIIT, high-intensity interval training.

Figure 6A displays a nonlinear, inverted U-shaped dose–response relationship between exercise volume and adiponectin levels. The peak significant effect of total exercise on adiponectin was observed at 880 METs-min/week (SMD = 1.34; 95% CrI: 0.75, 1.96; SD = 0.30). When the total weekly exercise dose exceeded 1,430 METs-min/week (SMD = 0.68; 95% CrI: −0.28, 1.65; SD = 0.49), the effect became nonsignificant (95% CrI includes 0). This dose–response pattern suggests that any exercise volume below 1,430 METs-min/week may significantly improve adiponectin levels in individuals with overweight or obesity (95% CrI does not include 0). At 600 METs-min/week, the predicted effect size was large [corresponding to the lower limit of energy expenditure recommended by the World Health Organization (29)] (SMD = 1.24; 95% CrI: 0.64, 1.86; SD = 0.31), and at 1,200 METs-min/week, the predicted effect size remained large [equivalent to the upper limit of WHO-recommended physical activity levels (29)] (SMD = 1.15; 95% CrI: 0.58, 1.73; SD = 0.29).

Figure 6B shows the dose–response curves for the various exercise modalities analyzed in this study with respect to their effects on adiponectin. We found an inverted U-shaped dose–response relationship for both AE and HIIT. The maximum significant effect for AE occurred at 780 METs-min/week (SMD = 1.65; 95% CrI: 0.79, 2.48; SD = 0.42), and for HIIT at 610 METs-min/week (SMD = 1.43; 95% CrI: 1.03, 2.6; SD = 0.59). When AE exceeded 1,360 METs-min/week or HIIT exceeded 910 METs-min/week, the effects became nonsignificant (95% CrI includes 0). COM showed a nonlinear dose–response relationship, with the minimum significant dose at 890 METs-min/week (SMD = 0.94; 95% CrI: 0.023, 1.9; SD = 0.47); effects became nonsignificant when COM exceeded 1,260 METs-min/week (95% CrI includes 0). RT exhibited a nonlinear, positively correlated dose–response pattern, with a minimum significant dose of 780 METs-min/week (SMD = 1.22; 95% CrI: 0.045, 2.38; SD = 0.58).

#### 3.6.2 Leptin

**Figure 7:**
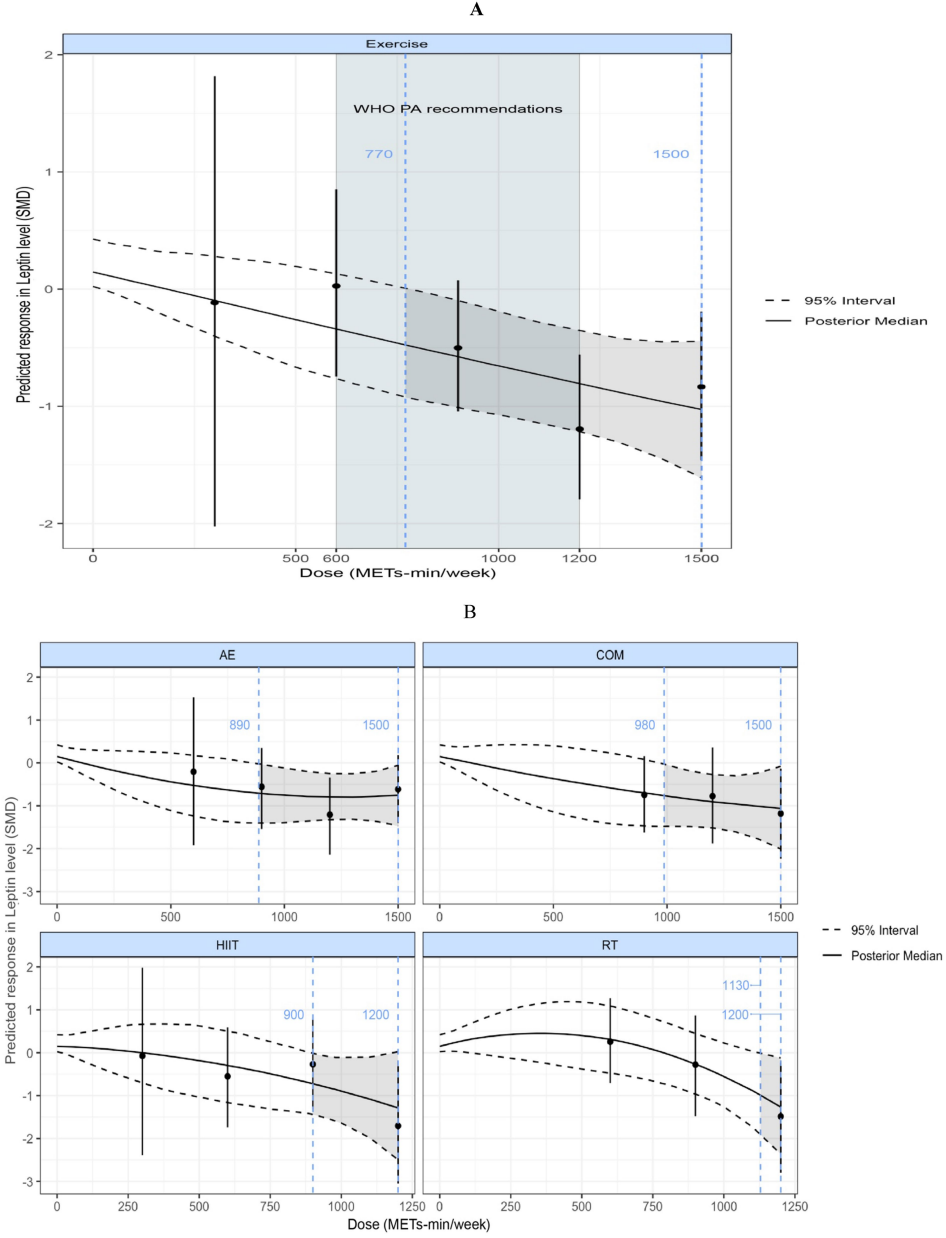
**(A)** Dose–response relationship between total weekly exercise volume and leptin levels in individuals with overweight or obesity; **(B)** Dose–response relationship between different types of weekly exercise and leptin levels in individuals with overweight or obesity. The blue dashed lines indicate the beginning and end of the significant 95% credible interval, and the blue numbers represent the corresponding dose values at these points (METs-min/week); the red dashed line and red numbers indicate the position and specific dose value at which the optimal dose–response occurs (METs-min/week). MET, metabolic equivalent of task; PA, physical activity; SMD, standardized mean difference; WHO, World Health Organization. AE, aerobic exercise; RT, resistance exercise; COM, AE combined RT; HIIT, high-intensity interval training.

Figure 7A illustrates a nonlinear, negatively correlated dose–response relationship between exercise volume and leptin levels. A significant effect began to appear at 770 METs-min/week (SMD = −0.49; 95% CrI: −0.94, −0.014; SD = 0.24), as the upper bound of the 95% CrI was less than 0. When the exercise volume exceeded 1,000 METs-min/week, the reduction in leptin levels accelerated (linear slope = 0.077 per 100 METs-min). At 1,200 METs-min/week, the predicted effect size was large [corresponding to the upper limit of WHO-recommended physical activity levels (29)] (SMD = −0.80; 95% CrI: −1.22, −0.35; SD = 0.21).

Figure 7B shows the dose–response curves of different exercise modalities on leptin. Nonlinear, negatively correlated dose–response relationships were observed for AE, COM, HIIT, and RT. RT required the highest minimum significant dose of 1,130 METs-min/week (SMD = −0.95; 95% CrI: −1.88, −0.004; SD = 0.47), followed by COM at 980 METs-min/week (SMD = −0.75; 95% CrI: −1.48, −0.015; SD = 0.37), HIIT at 900 METs-min/week (SMD = −0.76; 95% CrI: −1.51, −0.017; SD = 0.37), and AE at 890 METs-min/week (SMD = −0.72; 95% CrI: −1.39, −0.012; SD = 0.35).

### 3.7 Meta-regression

Given the significant heterogeneity observed between studies in the pairwise meta-analysis, it was necessary to conduct further meta-regression analyses to explore potential sources of heterogeneity and examine the relationships between various covariates and intervention effect sizes. The covariates investigated included age, sex, BMI change rate, and body fat percentage change rate (%BF change rate).

**Figure 8:**
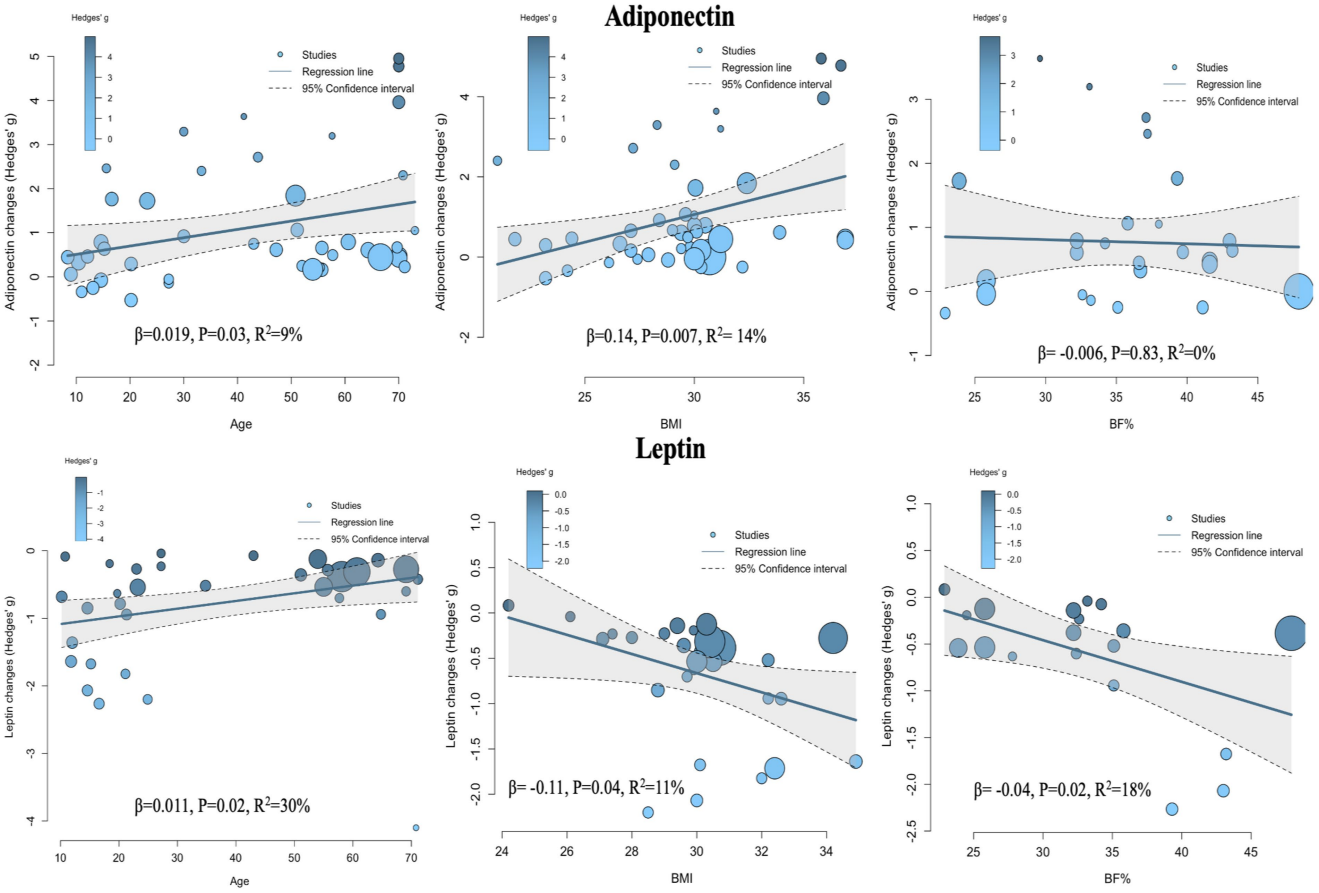
Meta-regression analysis of the influence of various regressors on effect size.

The meta-regression results for adiponectin (Figure 8, Adiponectin section; Table 2) showed that when age was used as a covariate, the effect size of exercise on adiponectin was significantly influenced, with a positive association—i.e., older age was associated with greater effect size (*β* = 0.019, *p* = 0.03, *R*^2^ = 9%). When BMI change rate was used as a covariate, a similarly significant positive association was observed (*β* = 0.14, *p* = 0.007, *R*^2^ = 14%). No statistically significant models were found when sex (*β* = −0.41, *p* = 0.3, *R*^2^ = 0%) and %BF change rate (*β* = −0.006, *p* = 0.83, *R*^2^ = 0%) were used as covariates.

The meta-regression results for leptin (Figure 8, Leptin section; Table 3) indicated that age was also a significant covariate, but in this case, a negative relationship was observed—i.e., as age increased, the effect size decreased (*β* = 0.011, *p* = 0.02, *R*^2^ = 30%). When BMI change rate and %BF change rate were used as covariates, both showed significant positive associations with effect size (*β* = −0.11, *p* = 0.04, *R*^2^ = 11%) and (*β* = −0.04, *p* = 0.02, *R*^2^ = 18%), respectively. No statistically significant association was found when sex was used as a covariate (*β* = −0.05, *p* = 0.85, *R*^2^ = 0%).

## 4 Discussion

### 4.1 Main findings

This study employed pairwise, network, dose–response meta-analyses, as well as meta-regression to comprehensively reveal the effects of different exercise modalities on the regulation of adipokines in individuals with overweight or obesity. Given the substantial heterogeneity in direct comparisons across studies in the pairwise meta-analysis, the results of the network meta-analysis (NMA) were primarily utilized.

NMA demonstrated that exercise interventions had a positive effect on both adipokines. Notably, when the two adipokines were analyzed separately, differences in the effects of the various exercise interventions were observed.

The NMA for Adiponectin showed that all four exercise interventions (HIIT, AE, COM, and RT) significantly improved adiponectin levels in individuals with overweight or obesity. No significant differences were observed between the intervention effects, and all produced large effect sizes (SMD > 0.70). The SUCRA ranking results indicated that HIIT had the highest probability of being the optimal intervention (68%), followed by RT (66%), AE (60%), and COM (56%). These findings suggest that all four exercise modalities can improve adiponectin levels, with relatively comparable effects. In the dose–response meta-analysis, a nonlinear, inverted U-shaped dose–response relationship was found between exercise volume and adiponectin. The maximum significant effect was observed at 880 METs-min/week, which is equivalent to 220 min of cycling (4 METs-min/week), 176 min of resistance training (5 METs-min/week), 176 min of combined aerobic and resistance training (5 METs-min/week), and 117 min of high-intensity exercise (7.5METs-min/week) (28). When the total exercise dose exceeded 1,430 METs-min/week, the effect became nonsignificant. Moreover, we found that the dose–response patterns differed by exercise modality: AE and HIIT exhibited an inverted U-shaped dose–response relationship with adiponectin; COM showed a nonlinear dose–response relationship; and RT presented a nonlinear, positive dose–response relationship with adiponectin. Notably, HIIT achieved the maximum significant effect at a relatively lower dose (610 METs-min/week). Finally, meta-regression analysis revealed that the intervention effect of exercise on adiponectin was positively correlated with age and BMI, while sex and %BF change rate did not affect the intervention effect.

The NMA for Leptin indicated that, except for RT, the other three interventions significantly reduced leptin levels in individuals with overweight or obesity. No significant differences in effect sizes were observed among the three interventions, and all produced large effect sizes (SMD > 0.70). The SUCRA ranking results showed that COM had the highest probability of being the optimal intervention (84%), followed by AE (69%), HIIT (68%), and RT (26%). In the dose–response meta-analysis, a nonlinear, negative dose–response relationship between exercise volume and leptin was observed, with the estimated minimum effective dose at 770 METs-min/week, corresponding to 193 min of cycling (4 METs-min/week), 154 min of resistance training (5 METs-min/week), 154 min of combined aerobic and resistance training (5 METs-min/week), and 103 min of high-intensity exercise (7.5METs-min/week) (28). When the exercise volume exceeded 1,000 METs-min/week, the reduction in leptin accelerated (linear slope = 0.077 per 100 METs-min). These findings suggest that additional benefits may be obtained from exercise even beyond the upper limit of WHO-recommended physical activity of 1,200 METs-min/week (29). Furthermore, we found that the dose–response patterns of the various exercise modalities with respect to leptin were all characterized by a nonlinear, negative relationship. RT required the highest minimum effective dose at 1,130 METs-min/week, whereas AE reached the lowest minimum effective dose at 890 METs-min/week. Finally, meta-regression analysis revealed that the intervention effect of exercise on leptin was negatively correlated with age, positively correlated with BMI and %BF change rate, while sex did not influence the intervention effect.

### 4.2 Mechanisms underlying exercise effects on adiponectin and leptin

Adiponectin: The present meta-analysis demonstrates that all major exercise modalities –HIIT, AE, COM, and RT–significantly elevate adiponectin levels in individuals with overweight and obesity (all with large effect sizes). This broad efficacy aligns with previous reports that physical exercise, even without other lifestyle changes, is associated with increased adiponectin in individuals with overweight and obesity (48–52). Mechanistically, exercise likely improves adipose tissue function and insulin sensitivity, thereby relieving obesity-related hypoadiponectinemia (13, 53). Aerobic-based modalities (including HIIT) induce caloric expenditure and fat loss, which can remove inhibitory effects of excess adiposity on adiponectin secretion (54, 55). Additionally, acute physiological responses to intense exercise – such as lactate accumulation, surges in catecholamines (e.g., adrenaline), glycogen depletion, and metabolic acidosis – have direct stimulatory effects on adiponectin release as observed in animal and human studies (56). This may explain why HIIT achieved maximal adiponectin gains at a relatively lower exercise dose in our dose–response analysis (≈610 MET-min/week). Resistance training, traditionally not thought to markedly influence adipokines, also yielded substantial adiponectin increases in our analysis. One potential mechanism is that RT increases lean muscle mass and resting energy expenditure, which in turn reduces fat mass and improves adipose tissue perfusion, facilitating adiponectin release into circulation (57, 58). There is evidence that resistance exercise can elevate adiponectin after sufficient training duration (e.g., >12–15 weeks) (59), and combining aerobic and resistance training may synergistically boost adiponectin more than aerobic training alone in metabolically at-risk individuals (50, 51, 60). Notably, our meta-regression found greater adiponectin responses in those with higher baseline BMI and older age, suggesting that individuals with more adipose tissue or age-related adiponectin declines have the most to gain from exercise-induced adiponectin improvements. In contrast, sex and the degree of body fat loss did not significantly modulate the adiponectin response, indicating that exercise can enhance adiponectin even in the absence of major weight reduction. This implies direct regulatory effects of exercise on adipose tissue endocrine function beyond just fat loss.

Leptin: Our findings indicate that aerobic, HIIT, and combined training produce large reductions in circulating leptin in individuals with overweight and obesity, whereas resistance training alone showed a more modest and non-significant effect on leptin. These patterns are consistent with prior studies reporting that endurance-type exercise is more effective than resistance exercise in lowering leptin levels (51, 61). The primary mechanism for leptin reduction with exercise is through achieving a negative energy balance and reducing adipose stores. Leptin, an “adipostat” hormone, is secreted proportional to fat mass under normal conditions (5, 62). Thus, sustained aerobic or high-intensity exercise that expends substantial calories leads to fat mass reduction and consequent lowering of leptin production (63). In our dose–response analysis, a weekly dose of ~770 MET-min of exercise (e.g., ~200–250 min of moderate activity) was the minimum threshold for significant leptin decreases, and greater doses were associated with progressively larger effects. This dose-dependent drop in leptin reflects the need for sufficient energy deficit to overcome leptin’s homeostatic maintenance of body fat. Moreover, exercise may acutely suppress leptin levels independently of fat loss – for example, short-term energy deficits (multi-day fasting or intense exercise) rapidly decrease circulating leptin even before observable fat loss (64). Exercise training can also improve leptin sensitivity at the cellular level: studies in animal models show aerobic training can down-regulate hypothalamic SOCS3 (suppressor of cytokine signaling) in the JAK/STAT pathway, reversing leptin resistance and lowering leptin concentrations for a given fat mass (59, 65–67). Improved leptin signaling means the body requires less leptin to regulate appetite and metabolism, which is a beneficial adaptation. The lack of a significant leptin decline with RT alone in our network meta-analysis likely owes to RT’s lower immediate energy expenditure and often minimal weight loss over short interventions. Some RT studies have reported no change in leptin unless accompanied by fat loss (68–70), though others show that when RT does induce a reduction in % body fat, it can lead to leptin decreases alongside muscle gain (57, 69, 71). Our meta-regression supports this: the percentage fat loss was positively correlated with the magnitude of leptin reduction, underscoring fat reduction as a key mediator. Nonetheless, RT may exert ancillary effects on leptin regulation via increased muscle-driven glucose uptake and acute exercise stress signals (lactate, catecholamines), which have been suggested to modestly lower leptin or enhance leptin sensitivity even without large fat changes (72). We also observed that younger individuals experienced slightly larger exercise-induced leptin declines than older adults, potentially because younger subjects achieve greater intensity or hormonal responses, whereas older adults may have blunted leptin dynamics or require longer intervention to elicit similar changes. Importantly, no sex differences were noted in leptin response to exercise in our analysis, in line with prior evidence that exercise reduces leptin in both men and women when equivalent fat loss is achieved (13). In summary, exercise – particularly modalities incorporating aerobic components – lowers leptin by reducing adiposity and improving energy-regulatory hormone function. Resistance training alone may need to be higher in volume or combined with aerobic work to significantly impact leptin, whereas any exercise form is effective in improving adiponectin. These mechanistic differences reinforce the value of a multimodal exercise approach for comprehensive adipokine modulation.

### 4.3 Clinical and public health implications

Significance of adipokine changes: The ability of exercise to increase adiponectin and decrease leptin carries important clinical implications for obesity management and metabolic health. Obesity is characterized by an adipokine profile of low adiponectin and high leptin (along with leptin resistance), which contributes to insulin resistance, systemic inflammation, and elevated cardiometabolic risk (5, 73). Adiponectin has anti-inflammatory and insulin-sensitizing properties; raising its level through exercise can improve glucose regulation and lipid metabolism (53, 73, 74). Indeed, low adiponectin predicts higher risk of type 2 diabetes and cardiovascular events, whereas higher adiponectin is protective (53, 74). Thus, the robust adiponectin increases observed with all exercise modalities in our study are encouraging for reducing obesity-related cardiometabolic complications. Higher adiponectin may mediate some of exercise’s beneficial effects on insulin sensitivity and endothelial function, potentially lowering the incidence of diabetes and atherosclerosis in the long term (2, 75, 76). Conversely, elevated leptin in obesity reflects leptin resistance and is associated with continued weight gain, dysregulated appetite, and cardiovascular strain (76). Reducing leptin levels via exercise (as seen with aerobic and combined training) likely indicates a reduction in fat mass and an improvement in leptin sensitivity, which could translate into better appetite control and energy balance. While a drop in leptin might theoretically increase hunger acutely, in the context of exercise-induced weight loss this is often offset by improved satiety signaling and other hormonal adaptations. Moreover, a lower leptin level for a given body fat content suggests restored sensitivity of the body’s energy-feedback mechanism (13). In sum, the adipokine changes from exercise are biomarkers of a healthier metabolic state – i.e., less visceral fat, improved adipose tissue function, and lower chronic inflammation (77, 78). These changes complement other benefits of exercise (improved fitness, blood pressure, lipid profile, etc.) and reinforce that exercise is a cornerstone therapy for obesity-related metabolic syndrome. Clinicians can consider monitoring adiponectin and leptin (or the adiponectin: leptin ratio) as emerging indicators of a patient’s response to an exercise intervention, as suggested by recent research (69).

### 4.4 Practical recommendations

Our findings provide practical guidance on exercise prescriptions for individuals with overweight and obesity, underscoring that multiple exercise modalities can be effective and should be tailored to the individual’s preferences, capabilities, and goals. Key insights for practice include:

Choose any sustainable modality: Since AE, HIIT, COM, and RT have all been shown to increase adiponectin levels (and all but RT alone can reduce leptin levels), patients have the flexibility to choose the type of exercise they prefer. This is crucial for enhancing adherence to exercise programs. The comparable efficacy among these exercise modalities (as indicated by our network meta-analysis showing no significant differences) suggests that the best exercise is the one an individual can consistently perform. For reducing leptin levels, it is advisable to include an aerobic component in the exercise regimen (such as steady-state endurance training or interval training). Adding resistance training will not diminish the benefits and may offer additional advantages in improving muscular strength and increasing lean body mass.

Dose and intensity matter: The dose–response patterns from our meta-analysis highlight that sufficient volume of exercise is required to significantly modulate adipokines. We found that about 880 MET-minutes per week is the dose associated with the largest adiponectin increase – roughly equivalent to 3–4 h of moderate exercise weekly (for example, ~250 min walking at 3.5 METs, or ~176 min resistance training at ~5 METs). Beyond this point, further increases in exercise volume yielded only marginal adiponectin gains (with a plateau and non-significant improvements beyond ~1,430 MET-min/week). This suggests there is an optimal moderate dose for boosting adiponectin, and exercising excessively may not continually raise adiponectin. In contrast, leptin levels showed a progressive decline with higher exercise doses, with a notable inflection once exceeding ~1,000 MET-min/week (approximately 250 min of moderate activity). This indicates that individuals with overweight and obesity may need to surpass the minimum physical activity guidelines to achieve substantial leptin reductions. For instance, while 150 min of moderate exercise per week (the basic public health recommendation) is a great start, increasing to 200–300 min per week (or including higher-intensity workouts to accumulate ~800–1,200 MET-min) can produce greater benefits in terms of leptin lowering and likely fat loss. Our findings support established weight-management guidelines that advocate higher volumes of exercise for individuals with obesity: the American College of Sports Medicine recommends at least 250 min/week of moderate exercise for significant weight loss, which corresponds to ~1,200–2000 MET-min/week (79). Similarly, our results showed continued leptin improvements even beyond the standard 300 min/week mark, affirming that “more is better” up to a point, as long as it is tolerable for the individual. Clinicians should encourage patients who are physically able to gradually increase their activity toward these higher volumes to maximize metabolic improvements. However, it is also crucial to personalize the plan – some individuals may achieve adequate results at lower volumes when combining exercise with dietary changes, whereas others may progress to higher doses under professional supervision for additional benefit (Tables 4, 5 present specific exercise recommendations based on different exercise types).

**Table 4:** Exercise recommendations for improving adiponectin levels in individuals with overweight and obesity.

Type of exercise	Minimum significant dose^1^ (METs-min/week)	Intensity	Energy expenditure^2^ (METs-min)	Minimum recommended Accumulation^3^ (min/week)	Minimum recommendations for exercise prescription^4^ (sessions × min/per week)
AE	780	Moderate	4.3 (code 17200)	~180	5 × ~ 40 6 × ~ 30
		Vigorous	7.5 (code 03016)	~105	3 × ~ 35 4 × ~ 30
COM	890	Moderate	4.3 (code 02035)	~210	5 × ~ 40 6 × ~ 35
		Vigorous	7.5 (mean of codes 01030, 02050)	~120	3 × ~ 40 4 × ~ 30
HIIT	610	Vigorous	7.5 (code 01015)	~85	2 × ~ 45 3 × ~ 30
RT	780	Moderate	5.0 (code 02052)	160	3 × ~ 55 4 × ~ 40
		Vigorous	6.0 (code 02050)	130	3 × ~ 45 4 × ~ 35

AE, Aerobic exercise; AE+RT, Aerobic combined with resistance training. HIIT, High-intensity interval training.

1 Values based on dose–response relationships derived from this study.

2 Intensity coding was extracted from the 2024 Compendium of Physical Activities (28).

3 Minimum weekly time of exercise.

4 The number of exercise sessions and exercise duration, excluding warm-up and cool-down periods.

**Table 5:** Exercise recommendations for reducing leptin levels in individuals with overweight or obesity.

Type of exercise	Minimum significant dose^1^ (METs-min/week)	Intensity	Energy expenditure^2^ (METs-min)	Minimum recommended accumulation^3^ (min/week)	Minimum recommendations for exercise prescription^4^ (sessions × min/per week)
AE	890	Moderate	4.3 (code 17200)	~210	5 × ~ 40 6 × ~ 35
		Vigorous	7.5 (code 03016)	~120	3 × ~ 40 4 × ~ 30
COM	980	Moderate	4.3 (code 02035)	~230	5 × ~ 50 6 × ~ 40
		Vigorous	7.5 (mean of codes 01030, 02050)	~130	3 × ~ 45 4 × ~ 35
HIIT	900	Vigorous	7.5 (code 01015)	~120	3 × ~ 40 4 × ~ 30
RT	1,130	Moderate	5.0 (code 02052)	~230	5 × ~ 50 6 × ~ 40
		Vigorous	6.0 (code 02050)	~190	4 × ~ 50 5 × ~ 40

AE, Aerobic exercise; AE+RT, Aerobic combined with resistance training. HIIT, High-intensity interval training.

1 Values based on dose–response relationships derived from this study.

2 Intensity coding was extracted from the 2024 Compendium of Physical Activities (28).

3 Minimum weekly time of exercise.

4 The number of exercise sessions and exercise duration, excluding warm-up and cool-down periods.

Individualized context: When prescribing exercise, consider the person’s baseline characteristics that might influence response. Our meta-regression suggests that those with higher BMI may see larger absolute drops in leptin (given more excess fat to lose) and potentially larger increases in adiponectin (perhaps because their baseline adiponectin is lower and more “correctable”). Thus, very individuals with obesity should be reassured that even modest exercise can start improving their adipokine profile, and continuing beyond initial weight-loss plateaus still yields internal benefits. Older individuals showed somewhat attenuated leptin responses, which implies they might require longer duration interventions or adjunctive dietary caloric restriction to achieve the same leptin reduction as a younger person. In practice, combining exercise with nutritional counseling in older adults could help overcome this and also combat age-related muscle loss while losing fat. On the other hand, older age was associated with greater gains in adiponectin in our analysis – a promising finding since older adults often have heightened cardiovascular risk. This could mean that exercise is particularly important in elderly overweight individuals to boost their adiponectin (and thereby vascular protection). In terms of sex, since men and women responded similarly, there is no need for sex-specific exercise prescriptions for adipokine benefit; both can follow the general principles above. Ultimately, an individualized exercise program–taking into account a person’s health status, schedule, and enjoyment – will maximize adherence and long-term success, which is crucial because sustained exercise is needed to maintain adipokine improvements.

### 4.5 Strengths and limitations

This study has several notable strengths. First, we employed an integrated approach combining pairwise, network, and state-of-the-art dose–response meta-analyses to examine the effects of exercise on adipokines in individuals with overweight or obesity. The use of these complementary methods allowed for a robust assessment of both the optimal exercise modality and dose. Second, the inclusion of a large sample of individuals with overweight or obesity, along with the detailed classification of exercise modalities, provided sufficient statistical power and enabled a broad range of comparisons. Furthermore, we conducted meta-regression analyses on several key variables to explore potential moderators of intervention effects, thereby enhancing the generalizability of our findings.

Nevertheless, several limitations should be acknowledged. First, variability in sample size and intervention duration across the included randomized controlled trials may have influenced the stability of the results. Second, inconsistencies in how exercise dose was measured and how intervention types were defined may have led to potential underestimation or overestimation of the true effects. Third, we observed substantial heterogeneity among studies in the pairwise meta-analysis, which may affect the reliability of those results. Additionally, according to the GRADE assessment, the overall certainty of evidence for most comparisons was rated as low to moderate, which may limit the robustness of the current findings. Furthermore, only English-language publications were included, which may have introduced language bias and potentially led to the omission of relevant data published in other languages. Future research should aim to address these limitations and further validate and extend our results, particularly regarding the identification of the optimal exercise dose and frequency for improving adipokine profiles.

## 5 Conclusion

In this comprehensive meta-analysis, we confirmed the effectiveness of exercise in regulating adipokines among individuals with overweight or obesity. Notably, different exercise modalities exerted distinct effects on specific adipokines. A significant dose–response relationship was observed between exercise intensity and duration and the magnitude of the intervention effects. Moreover, changes in age and body composition (BMI and body fat percentage) were significantly associated with the observed intervention outcomes. Our findings, supported by low to moderate quality evidence, indicate that COM, HIIT, AE are all effective in improving adipokine profiles in this population, with no significant differences in efficacy among them. RT demonstrated a significant benefit in increasing adiponectin levels but did not show a significant effect in reducing leptin levels. Improving inflammatory profiles in individuals with overweight or obesity is of considerable clinical importance for the prevention of obesity- and inflammation-related comorbidities such as type 2 diabetes, metabolic syndrome, and cardiovascular disease. Future studies should further investigate the synergistic effects of exercise combined with dietary interventions on adipokine regulation, and focus on developing individualized exercise prescriptions to enhance overall health outcomes in this population.

## Data Availability

The original contributions presented in the study are included in the article/Supplementary material, further inquiries can be directed to the corresponding author.
