# Supplementary material for: Unveiling the perfect workout: exercise modalities and dosages to ameliorate adipokine dysregulation in individuals with overweight and obesity: a systematic review with pairwise, network, and dose–response meta-analyses
Source: Front Nutr. 2025 Aug 12;12:1653449. doi: 10.3389/fnut.2025.1653449 (PMC12381787; doi:10.3389/fnut.2025.1653449)
Supplement: Supplementary file 1 [file Data_Sheet_1.PDF]

Unveiling the Perfect Workout: Exercise Modalities and Dosages to Ameliorate Adipokine Dysregulation in individuals with overweight and obesity: A Systematic Review with Pairwise, Network, and Dose–Response Meta-Analyses

Hai Wang[1], Hao Wang[1], Enyan Zhang[1] and Xiaodong Liu[1]

Supplemental materials

**Appendix 1** Search terms	2
**Appendix 2** The classifications of exercise training	3
**Appendix 3** Characteristics of the included studies	4-9
**Appendix 4** Risk of Bias Assessment Based on the Cochrane Handbook	10-11
**Appendix 5** Details of pairwise meta-analyses	12
**Appendix 6** Funnel plots and Egger's test	13-14
**Appendix 7** Model fit summaries for included studies	15
**Appendix 8** MCMC Convergence Diagnostics	16-17
**Appendix 9** MCMC Sampling Trajectories and Posterior Distributions	18-20
**Appendix 10** Node-split for all studies	21-22
**Appendix 11** SUCRA table for all studies	23
**Appendix 12** GRADE Evidence Quality Assessment for adiponectin and Leptin	24-27
**Appendix 13** Key Assumptions of Dose-Response Network Meta-Analysis	28-35
**Appendix 14** Model Selection for Dose-Response Analysis	36-42
**Appendix 15** Dose-response relationships	43-51

Appendix 1 Search terms

Exercise [MeSH Terms] OR training OR aerobic exercise OR aerobic training OR moderate intensity continuous training OR resistance training OR resistance exercise OR strength training OR strength exercise OR combined training OR combined exercise OR concurrent training OR high intensity interval training OR sprint interval training OR walking OR physical activity AND Obesity [MeSH Terms] OR obese OR overweight AND Inflammation [MeSH descriptor] OR Inflammat* OR interleukin OR Adiponectin OR Leptin AND Randomized controlled trial OR randomized OR placebo

Appendix 2 The classifications of exercise training

Type	Definition
CON	No exercise
AE (Aerobic Exercise)	Aerobic exercise refers to a form of physical activity performed at a moderate-to-low intensity over an extended duration under conditions of sufficient oxygen supply. Its primary objective is to improve cardiorespiratory endurance and overall metabolic health. Common forms of aerobic exercise include brisk walking, jogging, cycling, swimming, and step machine training.
RT (Resistance Training)	Resistance training, also known as strength training, involves applying external resistance—such as free weights, exercise machines, resistance bands, or one's own body weight—to the muscles. The goal is to enhance muscle strength, endurance, and mass.
HIIT (High-Intensity Interval Training)	High-intensity interval training (HIIT) is a training modality that alternates between short bouts of high-intensity exercise and periods of low-intensity recovery or rest. The high-intensity phases are typically performed at near-maximal efforts (e.g., 85% to 95% of an individual's maximum heart rate) for durations ranging from 20 seconds to several minutes, while the recovery phases are intended to reduce the heart rate and facilitate physiological recovery.
COM(AE combined with RT)	This training modality integrates the benefits of aerobic exercise and resistance training within a single regimen, aiming to simultaneously enhance cardiorespiratory fitness and muscular strength.

Appendix 3 Characteristics of the included studies

Study	Country	N,Sex (female/male)	Age (mean[SD])	BMI (mean[SD])	Fat%(mean[SD])	Complication	Exercise category	Intervention duration (weeks)	Frequency	Time /session	Exact dose	Dose	Residual dose	Outcomes
Armamento2020[1]	USA	40 (26/14)	70 (4.0)	35.9 (4.4)	NR		AE	24	3	60	720	600	120	Adiponectin
		40 (25/15)	70 (5.0)	36.7 (5.8)	NR		RT				720	600	120	
		40 (24/16)	70 (5.0)	35.8 (4.5)	NR		AE+RT				1080	1200	120	
		40 (28/12)	70 (5.0)	36.7 (5.0)	NR		CON							
Abbenhardt2013[2]	USA	53 F	58.1 (5.0)	30.7 (3.7)	47.9 (4.1)		AE	52	5	45	1687.5	1500	187.5	Leptin, Adiponectin
		42 F	57.4 (4.4)	30.7 (3.9)	47.8 (4.5)		CON							
Aghajani2022[3]	Iran	12 F	33.3 (1.9)	18-23	NR		AE	8	3	60	432	300	132	Adiponectin
		12 F	35.1 (1.5)	18-23	NR		CON							
Akbarpour2013[4]	Iran	30 M	23.2 (2.5)	30.05 (2.26)	23.89 (2.73)	coronary heart disease	AE	12	3	30	810	900	90	Leptin, Adiponectin
		30 M	22.7 (2.7)	30.60 (2.45)	24.58 (2.61)		CON							
Almenning2015[5]	Norway	8 F	27.2 (5.5)	26.1 (6.5)	33.2 (9.6)	Polycystic Ovary Syndrome	HIIT	10	3	30	765	900	135	Leptin, Adiponectin
		8 F	27.2 (5.5)	27.4 (6.9)	32.6 (10.4)		RT			30	765	900	135	
		9 F	27.2 (5.5)	26.5 (5.0)	34.2 (6.9)		CON							
Bagheri2020[6]	Iran	15 M	43.8 (3.4)	27.2 (0.5)	37.1 (2.9)		AE	8	3	60	900	900	0	Adiponectin
		15 M	44.0 (3.5)	27.8 (0.3)	39.7 (2.4)		CON							
Bagheri2024[7]	Iran	15 M	30 (3)	28.4 (1.4)	NR		RT	12	3	50	1200	1200	0	Adiponectin
		15 M	31 (4)	28.7 (1.7)	NR		CON							
Baitul2016[8]	Indonesia	12 F	46.7 (1.3)	31.3 (1.0)	31.6 (1.9)		AE	8	2	40	360	300	60	Adiponectin
		12 F	46.8 (1.4)	32.5 (1.6)	31.8 (1.6)		CON							
Balducci2010[9]	Italy	20 (8/12)	64.3 (8.1)	29.4 (1.1)	32.2 (2.4)	T2DM	AE	52	2	60	936	900	36	Leptin, Adiponectin
		22 (8/14)	60.6 (9.3)	30.5 (0.9)	32.2 (1.8)		AE+RT				936	900	36	

		20 (9/11)	61.1 (7.1)	30.9 (1.1)	33.2 (2.3)		CON							
Bharath2018[10]	USA	20 F	14.6 (1.0)	30.0 (2.2)	43.0 (2.3)		AE+RT	12	5	50	1425	1500	75	Leptin, Adiponectin
		20 F	14.8 (1.0)	30.0 (1.2)	43.4 (2.2)		CON							
Bonfante2022[11]	Brazil	17 (10/7)	51.1 (3.9)	29.6 (3.6)	35.8 (5.2)	T2DM	AE+RT	16	3	75	945	900	45	Leptin, Adiponectin
		17 (8/9)	52.4 (4.4)	29.4 (3.3)	34.4 (6.9)		CON							
Bouchonville2013a[12]	USA	26 (16/10)	70.0 (4.0)	36.9 (5.4)	41.6 (9.4)		AE+RT	24	3	60	945	900	45	Adiponectin
		27 (18/9)	69.0 (4.0)	37.3 (4.7)	43.8 (9.9)		CON							
Bouchonville2013b[12]	USA	26 (16/10)	70.0 (4.0)	36.9 (5.4)	41.6 (9.4)		AE+RT	52	3	60	945	900	45	Adiponectin
		27 (18/9)	69.0 (4.0)	37.3 (4.7)	43.8 (9.9)		CON							
Campos2017[13]	Brazil	39 (NR)	16 (1.7)	35.6 (4.1)	40.6 (7.1)		AE	52	3	60	1260	1200	60	Adiponectin
		63 (NR)	16 (1.7)	37.2 (5.0)	40.6 (7.1)		AE+RT			60	945	900	45	
Chae2010[14]	Korea	19 (NR)	10.4 (3.1)	26.6 (0.8)	36.7 (1.4)		AE+RT	12	2	90	585	600	15	Adiponectin
		19 (NR)	10.6 (3.8)	26.2 (1.0)	35.5 (1.0)		CON							
Chuensiri2018[15]	Thailand	11 M	11.0 (0.3)	24.2 (1.0)	22.9 (1.6)		HIIT	12	3	20	900	900	0	Leptin, Adiponectin
		11 M	10.6 (0.3)	26.1 (1.0)	25.3 (1.6)		CON							
Coker2009[16]	USA	6 (3/3)	73.0 (2.0)	30.0 (1.0)	38.0 (3.0)		AE	12	(4-5)	30	900	900	0	Adiponectin
		6 (3/3)	67.0 (3.0)	31.0 (1.0)	40.0 (2.0)		CON							
Damaso2014[17]	Brazil	55(NR)	16.89 (1.81)	35.7 (4.3)	41.4 (7.4)		AE	52	3	60	1440	1500	60	Leptin, Adiponectin
		61(NR)	16.89 (1.81)	36.7 (4.9)	46.7 (5.1)		AE+RT				855	900	45	
El-Kader2016[18]	Saudi Arabia	50 (16/34)	50.8 (5.3)	32.4 (2.5)	NR	NASH	AE	12	3	30	630	900	70	Leptin, Adiponectin
		50 (14/36)	51.1 (5.9)	31.8 (2.9)	NR		CON							
Elia2014[19]	Brazil	24(17/7)	15-19	35.1 (3.9)	41.8 (6.6)		AE	24	3	60	1350	1500	150	Leptin
		24(15/9)	15-19	35.1 (4.7)	45.6 (6.0)		AE+RT				1125	1200	75	
Fatouros2005a[20]	Greece	14 M	71.1 (3.6)	30.1 (3.5)	NR		RT	24	3	60	720	600	120	Leptin, Adiponectin
		10 M	69.8 (5.1)	28.7 (2.1)	NR		CON							

Fatouros2005b[20]	Greece	12 M	69.7 (3.8)	29.0 (2.8)	NR		RT	24	3	60	900	900	0	Leptin, Adiponectin
		10 M	69.8 (5.1)	28.7 (2.1)	NR		CON							
Fatouros2005c[20]	Greece	14 M	70.8 (2.8)	29.1 (4.0)	NR		RT	24	3	60	1170	1200	30	Leptin, Adiponectin
		10 M	69.8 (5.1)	28.7 (2.1)	NR		CON							
Frank2005[21]	USA	87 F	60.7 (6.7)	30.4 (4.1)	47.4 (4.8)		AE	12	5	45	1080	1200	120	Leptin
		85 F	60.6 (6.8)	30.5 (3.7)	47.3 (4.6)		CON							
Hara2005a[22]	Japan	7 M	19.7 (1.3)	29.9 (1.8)	27.8 (3.0)		AE	8	3	50	975	900	25	Leptin, Adiponectin
		7 M	19.4 (1.0)	33.5 (5.6)	29.3 (3.8)		CON							
Hara2005b[22]	Japan	7 M	18.4 (0.5)	29.9 (3.8)	24.5 (3.6)		AE+RT	20	3	80	1680	1500	180	Leptin, Adiponectin
		7 M	19.4 (1.0)	33.5 (5.6)	29.3 (3.8)		CON							
Horvath2024[23]	Hungary	20 (16/4)	59 (17.04)	43.91 (37.44)	51.05 (14.67)		AE+RT	6	5	90	1800	1500	300	Adiponectin
		20 (13/7)	56.61 (20.74)	41.57 (13.92)	47.34 (20.22)		AE+RT				1350	1200	150	
Johannsen2012[24]	Spain	92 F	56.6 (6.5)	31.2 (3.5)	NR		AE	24	4	75	1650	1500	150	Adiponectin
		86 F	57.1 (5.7)	32.1 (3.9)	NR		CON							
Kadoglou2007[25]	Greece	30 (17/13)	59.3 (4.8)	32.1 (3.2)	38.5 (7.1)	T2DM	AE	24	4	45	1350	1500	150	Adiponectin
		30 (18/12)	63.8 (7.0)	32.0 (3.4)	40.1 (6.9)		CON							
Karacabey2009[26]	Turkey	20 M	11.8 (0.5)	34.9 (4.1)	NR		AE	12	3	45	1080	1200	120	Leptin
		20 M	11.2 (0.8)	35.5 (3.2)	NR		CON							
Kelly2007[27]	USA	9 (5/4)	10.8 (0.7)	32.7 (2.6)	45.9 (2.1)		AE	8	4	50	1400	1500	100	Leptin, Adiponectin
		10 (6/4)	11.0 (0.7)	30.5(2.3)	44.6 (2.5)		CON							
Khademosharie2023[28]	Iran	15 F	35.4 (6.9)	28.99 (1.73)	39.71 (4.24)		HIIT	8	3	30	900	900	0	Leptin
		10 F	35.4 (6.9)	28.97 (3.44)	38.89 (3.02)		CON							
Kim2019[29]	Korea	10 M	69.1 (0.9)	26.2 (0.5)	32.4 (1.4)		AE+RT	12	3	100	1400	1500	100	Leptin
		10 M	68.5 (0.9)	26.0 (0.4)	32.7 (1.8)		CON							

Kim2014[30]	Korea	29 M	24.9 (2.8)	28.5 (2.4)	27.4 (2.4)	T2DM	AE	8	4	50	1800	1500	300	Leptin
		10 M	26.6 (2.8)	28.2 (2.4)	26.6 (3.0)		CON							
KU2010[31]	Korea	13 F	55.7 (6.2)	27.1 (2.3)	NR	T2DM	RT	12	5	60	1050	1200	150	Leptin, Adiponectin
		15 F	55.7 (7.0)	27.1 (2.4)	NR		AE				1290	1200	90	
		16 F	57.8 (8.1)	27.4 (2.8)	NR		CON							
Li2022[32]	China	18 F	12.0 (1.0)	24.6 (3.4)	NR		AE	16	4	50	1400	1500	100	Leptin
		18 F	11.6 (0.3)	24.1 (3.1)	NR		CON							
Lopes2016[33]	Brazil	17 F	14.6 (1.2)	28.8 (3.6)	NR		AE+RT	12	3	60	1215	1200	15	Leptin, Adiponectin
		16 F	14.4 (1.2)	29.4 (3.1)	NR		CON							
Marcell2005[34]	USA	17 (NR)	47.2 (9.2)	33.9 (4.9)	39.7 (8.3)		AE	16	5	30	525	600	75	Adiponectin
		14 (NR)	44.1 (9.5)	35.3 (3.7)	43.7 (6.4)		CON							
Miller2004[35]	USA	79 (60/19)	69.1 (6.5)	34.2 (4.8)	NR	knee osteoarthritis	AE+RT	24	3	50	930	900	30	Leptin
		76 (51/25)	68.7 (6.2)	34.3 (5.0)	NR		CON							
Moghadasi2012[36]	Iran	8 M	41.18 (6.1)	31.0 (2.1)	29.6 (3.1)		AE	12	4	45	1386	1500	114	Adiponectin
		8 M	41.18 (6.1)	32.0 (5.3)	31.4 (5.5)		CON							
Murphy2009[37]	USA	23 (NR)	7—12	27.9 (4.8)	NR		AE	12	5	30	750	600	150	Adiponectin
		12 (NR)	7—12	31.8 (5.0)	NR		CON							
Nunes2018[38]	Brazil	13 F	62.9 (8.8)	30.6 (3.9)	44.5 (3.1)		AE+RT	12	3	60	819	900	81	Leptin, Adiponectin
		13 F	62.3 (6.9)	31.4 (6.6)	45.5 (5.3)		HIIT			30	630	600	30	
Oh2017[39]	Japan	19 M	51.2 (1.9)	27.2 (0.9)	NR	NAFLD	RT	12	2	40	480	600	120	Leptin, Adiponectin
		20 M	48.6 (1.8)	28.4 (0.9)	NR		HIIT				300	300	0	
		13 M	48.2 (2.3)	28.8 (1.1)	NR		AE				480	600	120	
Park2012[40]	Korea	15 (8/7)	12.1 (0.1)	24.4 (0.4)	NR		AE+RT	12	3	60	963	900	63	Adiponectin
		14 (7/7)	12.2 (0.1)	24.3 (0.3)	NR		CON							
Phillips2012[41]	USA	11 F	64.8 (2.4)	32.2 (3.3)	35.1 (2.7)		RT	12	3	50	525	600	75	Leptin, Adiponectin

		12 F	66.4 (2.8)	33.7 (4.4)	36.4 (3.3)		CON							
Racil2015[42]	Tunisia	23 F	16.6 (0.9)	NR	39.3 (1.7)		HIIT	12	3	15	495	600	105	Leptin, Adiponectin
		19 F	16.9 (1.0)	NR	36.7 (2.0)		CON							
Racil2013[43]	Tunisia	11 F	15.6 (0.7)	NR	37.2 (1.2)		HIIT	12	3	15	495	600	105	Adiponectin
		12 F	15.9 (1.2)	NR	35.8 (1.5)		CON							
Rajabi2022[44]	Iran	8 F	57.6 (6.8)	31.2 (1.5)	33.1 (2.2)	T2DM	AE	8	3	40	720	600	120	Adiponectin
		8 F	56.9 (5.1)	34.0 (3.4)	34.6 (3.1)		CON							
Rezaeeshirazi2021[45]	Iran	13 M	21.1 (2.4)	32.0 (1.3)	NR		AE	8	4	45	1440	1500	60	Leptin
		14 M	21.3 (1.9)	32.6 (1.7)	NR		RT			30	720	600	120	
		15 M	22.5 (2.0)	32.5 (1.5)	NR		CON							
Salus2022[46]	Estonia	14 M	13.1 (1.3)	30.3 (3.2)	41.1 (1.3)		HIIT	12	3	20	660	600	60	Leptin, Adiponectin
		14 M	13.7 (1.6)	32.6 (5.9)	39.6 (1.9)		CON							
Sasimontonkul2024[47]	Thailand	10 F	33-55	> 23	34.20 (3.21)		HIIT	16	3	40	1200	1200	0	Leptin, Adiponectin
		11 F	33-55	> 23	35.13 (3.31)		CON							
sengun2024[48]		12 F	18-28	25-29.9	NR		AE	8	5	60	1800	1500	300	Leptin
		12 F	18-28	25-29.9	NR		CON							
Shokri2021[49]	Iran	15 F	8.4 (0.8)	21.8 (1.5)	36.6 (9.7)		AE+RT	12	3	50	847.5	900	52.5	Adiponectin
		15 F	8.3 (0.6)	21.8 (1.7)	36.3 (9.6)		CON							
Silva2024[50]	Portugal	18(14/4)	55.15 (5.72)	26.31 (3.51)	NR		AE+RT	16	3	60	1350	1200	150	Leptin, Adiponectin
		18(15/3)	52.24 (7.84)	28.18 (4.17)	NR		CON							
Soltani2023[51]	Iran	13 F	20.4 (1.5)	31 (4.1)	39.6 (2.8)		AE+RT	10	4	28	921.6	900	21.6	Adiponectin
		11 F	20.3 (1.4)	29.7 (3.5)	39.3 (2.2)		HIIT				902.4	900	2.4	
Suder2024[52]	Poland	16 M	34.8 (6.0)	32.2 (4.3)	35.1 (3.9)		AE+RT	6	3	50	1080	1200	120	Leptin
		12 M	34.1 (5.5)	31.7 (3.7)	36.3 (4.1)		CON							
Tan2025[53]	UK	10 F	57.7 (4.8)	29.7 (3.5)	NR		HIIT	8	3	20	780	900	120	Leptin, Adiponectin
		10 F	54.7 (3.3)	31.5 (10.67)	NR		CON							

Tjønna2008[54]	Norway	11 (7/4)	52.0 (10.6)	29.8 (5.5)	NR		AE	16	3	50	900	900	0	Adiponectin
		8 (4/4)	55.3 (13.2)	29.4 (4.9)	NR		HIIT			30	616	600	16	
		9 (4/5)	49.6 (9.0)	32.1 (3.3)	NR		CON							
Trapp2008[55]	Australia	15 F	20.2 (2.0)	23.2 (2.0)	NR		HIIT	15	3	30	900	900	0	Leptin, Adiponectin
		15 F	20.2 (2.0)	23.2 (2.0)	NR		AE			40	840	900	60	
		15 F	20.2 (2.0)	23.2 (2.0)	NR		CON							
Vella2017[56]	USA	8 (6/2)	23.1 (6.6)	29.9 (3.3)	35.2 (6.8)		HIIT	8	4	20	592	600	8	Leptin, Adiponectin
		9 (4/5)	28.9 (8.1)	33.1 (6.0)	35.3 (7.2)		AE			30	660	600	60	
Venojarvi2012[57]	Finland	36 M	54.0 (6.1)	30.3 (3.2)	25.8 (4.5)	metabolic syndrome	RT	12	3	50	900	900	0	Leptin, Adiponectin
		39 M	55.0 (6.2)	30.0 (3.4)	25.8 (5.9)		AE				1050	1200	150	
		40 M	54.0 (7.2)	28.6 (3.0)	23.6 (5.0)		CON							
Wong2018[58]	USA	15 F	15.2 (4.6)	30.1 (8.5)	43.2 (8.9)		AE+RT	12	3	50	757.5	900	142.5	Leptin, Adiponectin
		15 F	15.3 (4.3)	30.2 (4.6)	43.3 (8.5)		CON							
Wright2024[59]	USA	58 M	66.6 (7.04)	31.2 (3.4)	NR		AE	24	3	50	1350	1500	150	Adiponectin
		59 M	67.0 (6)	31.8 (4.8)	NR		CON							
Yetgin2018[60]	Turkey	8 M	17.0 (0.7)	32.2 (3.2)	33.1 (3.4)		AE	24	3	60	1080	1200	120	Leptin
		8 M	16.6 (1.0)	32.5 (3.1)	33.0 (3.4)		RT				1080	1200	120	
zadeh2022[61]	Iran	17 M	52.2 (4.8)	31.8 (1.3)	36.4 (4.5)	T2DM	HIIT	12	4	30	1080	1200	120	Leptin
		17 M	51.6 (5.0)	31.6 (1.6)	34.2 (3.9)		RT			30	660	600	60	
		18 M	52.8 (3.1)	31.2 (1.1)	35.5 (3.1)		AE			30	1080	1200	120	
		16 M	53.2 (3.9)	32.2 (2.0)	35.2 (4.2)		AE+RT			60	1600	1500	100	

Appendix 4 Risk of Bias Assessment Based on the Cochrane Handbook

Table 1. Risk of Bias Assessment Based on the Cochrane Handbook

Study	Allocation generation	Concealment of allocation	Blinding of outcome assessment	incomplete outcome data	Selective reporting	Other bias	Risk category
Armamento2020	L	L	L	L	L	L	L
Abbenhardt2013	L	U	U	L	L	L	L
Aghajani2022	L	L	L	U	L	L	L
Akbarpour2013	L	L	U	L	L	L	L
Almenning2015	L	H	L	H	L	H	H
Bagheri2020	L	L	U	U	L	L	L
Bagheri2024	L	L	U	U	L	L	L
Baitul2016	U	U	U	U	U	L	M
Balducci2010	L	L	U	L	L	L	L
Bharath2018	L	L	U	U	L	L	L
Bonfante2022	U	U	U	U	L	L	M
Bouchonville2013	L	L	L	L	L	L	L
Campos2017	U	U	U	L	L	H	H
Chae2010	U	U	U	L	L	L	L
Chuensiri2018	L	L	U	H	L	L	M
Coker2009	U	U	U	U	L	H	H
Damaso2014	L	U	U	L	L	L	L
El-Kader2016	L	L	L	L	L	L	L
Elia2014	L	U	U	H	L	L	M
Fatouros2005	U	U	U	U	L	L	M
Frank2005	U	U	L	L	L	H	M
Hara2005	L	U	U	L	L	H	M
Horvath2024	L	L	L	U	L	L	L
Johannsen2012	L	L	L	L	L	L	L
Kadoglou2007	U	U	U	L	L	L	L
Karacabey2009	U	U	U	U	L	L	M
Kelly2007	U	U	U	L	L	H	H
Khademosharie2023	L	L	U	U	L	L	L
Kim2019	L	L	U	L	L	L	L
Kim2014	U	U	U	L	L	L	L
KU2010	U	U	U	L	L	L	L
Li2022	U	U	U	L	L	L	L
Lopes2016	U	U	U	L	L	L	L
Marcell2005	L	U	U	L	L	U	L
Miller2004	U	U	U	L	L	L	L
Moghadasi2012	U	U	U	L	L	H	H
Murphy2009	U	U	U	L	L	U	M
Nunes2018	L	L	U	U	L	L	L

Oh2017	L	L	U	H	L	L	M
Park2012	U	U	U	L	L	L	L
Phillips2012	U	U	U	U	L	L	M
Racil2015	U	U	U	L	L	L	L
Racil2013	U	U	U	U	L	L	M
Rajabi2022	U	U	U	U	L	H	H
Rezaeeshirazi2021	U	U	U	L	L	L	L
Salus2022	H	H	U	U	L	L	H
Sasimontonkul2024	L	L	U	L	L	U	L
sengun2024	L	U	U	U	U	L	M
Shokri2021	L	L	U	L	L	L	L
Silva2024	L	L	L	L	L	L	L
Soltani2023	L	L	U	U	L	L	L
Suder2024	L	L	U	U	L	L	L
Tan2025	L	L	L	L	L	L	L
Tjønna2008	L	U	L	L	L	L	L
Trapp2008	U	U	U	L	L	L	L
Vella2017	U	U	U	L	L	H	H
Venojarvi2012	U	U	U	L	L	L	L
Wong2018	U	U	U	U	L	L	M
Wright2024	L	L	U	U	L	L	L
Yetgin2018	U	U	U	U	L	H	H
zadeh2022	L	L	U	L	L	L	L

Pairwise meta-analyses and publication bias

Appendix 5 Details of pairwise meta-analyses

1、 Adiponectin

Table 2. Details of pairwise meta-analyses

Comparison	Number of studies	SMD	95%CrI	I^2
COM vs CON	12	**0.85**	**(0.24,1.5)**	78.37%
RT vs CON	9	**0.99**	**(0.26,1.7)**	87.66%
AE vs CON	21	**0.92**	**(0.47,1.4)**	92.85%
HIIT vs CON	9	0.56	(-0.18,1.3)	75.73%

Note: AE: aerobic exercise, RT: resistance exercise, COM: AE combined RT, HIIT: high-intensity interval training, CON: control, Bolded numbers indicate the presence of significance, SMD: Standardized Mean Difference, CrI: credible interval.

2、Leptin

Table 3. Details of pairwise meta-analyses

Comparison	Number of studies	SMD	95%CrI	I^2
COM vs CON	10	**-0.80**	**(-1.4,-0.22)**	70.28%
HIIT vs CON	7	-0.46	(-1.2,0.24)	81.66%
AE vs CON	15	**-0.91**	**(-1.4,-0.40)**	79.82%
RT vs CON	8	**-0.77**	**(-1.4,-0.13)**	75.75%

Note: AE: aerobic exercise, RT: resistance exercise, COM: AE combined RT, HIIT: high-intensity interval training, CON: control, Bolded numbers indicate the presence of significance, SMD: Standardized Mean Difference, CrI: credible interval.

Appendix 6 Funnel plots and Egger's test

1、adiponectin

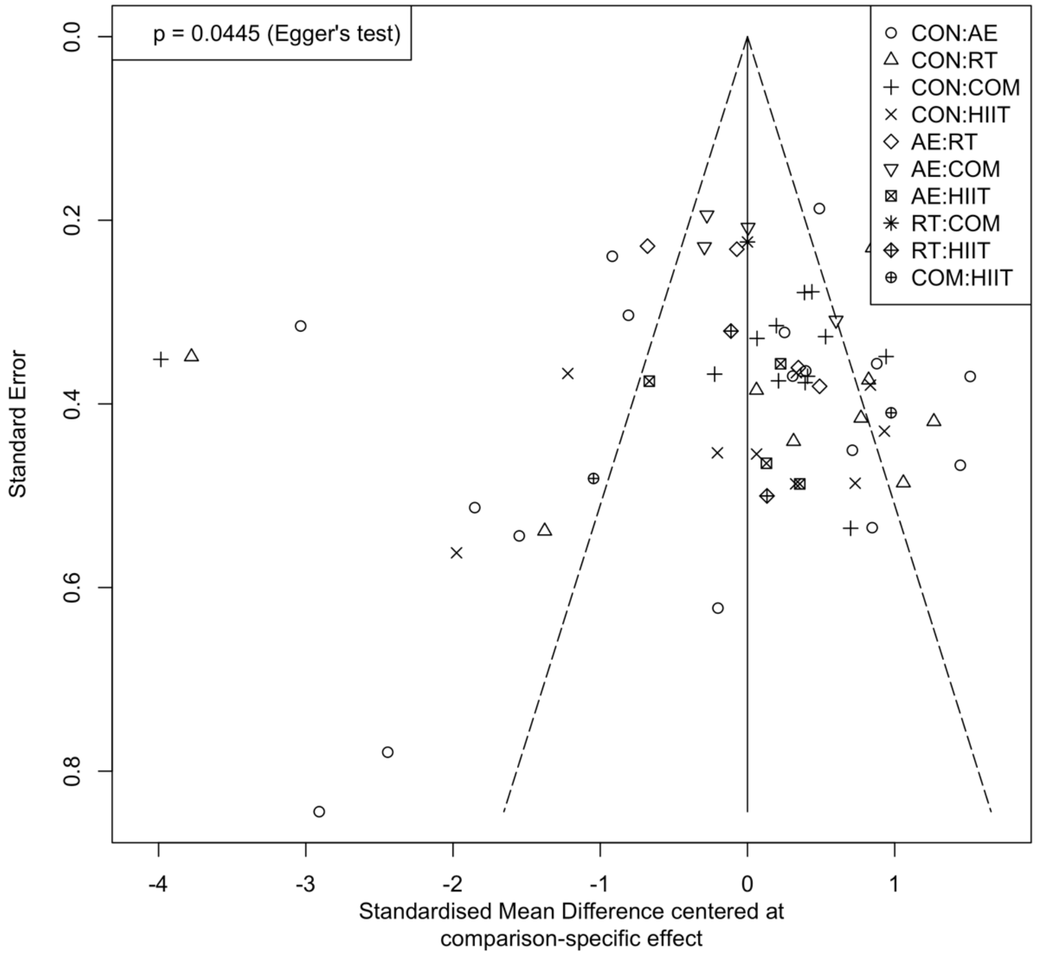

Figure 1. Funnel plot.

Note: AE: aerobic exercise, RT: resistance exercise, COM: AE combined RT, HIIT: high-intensity interval training, CON: control

2、Leptin

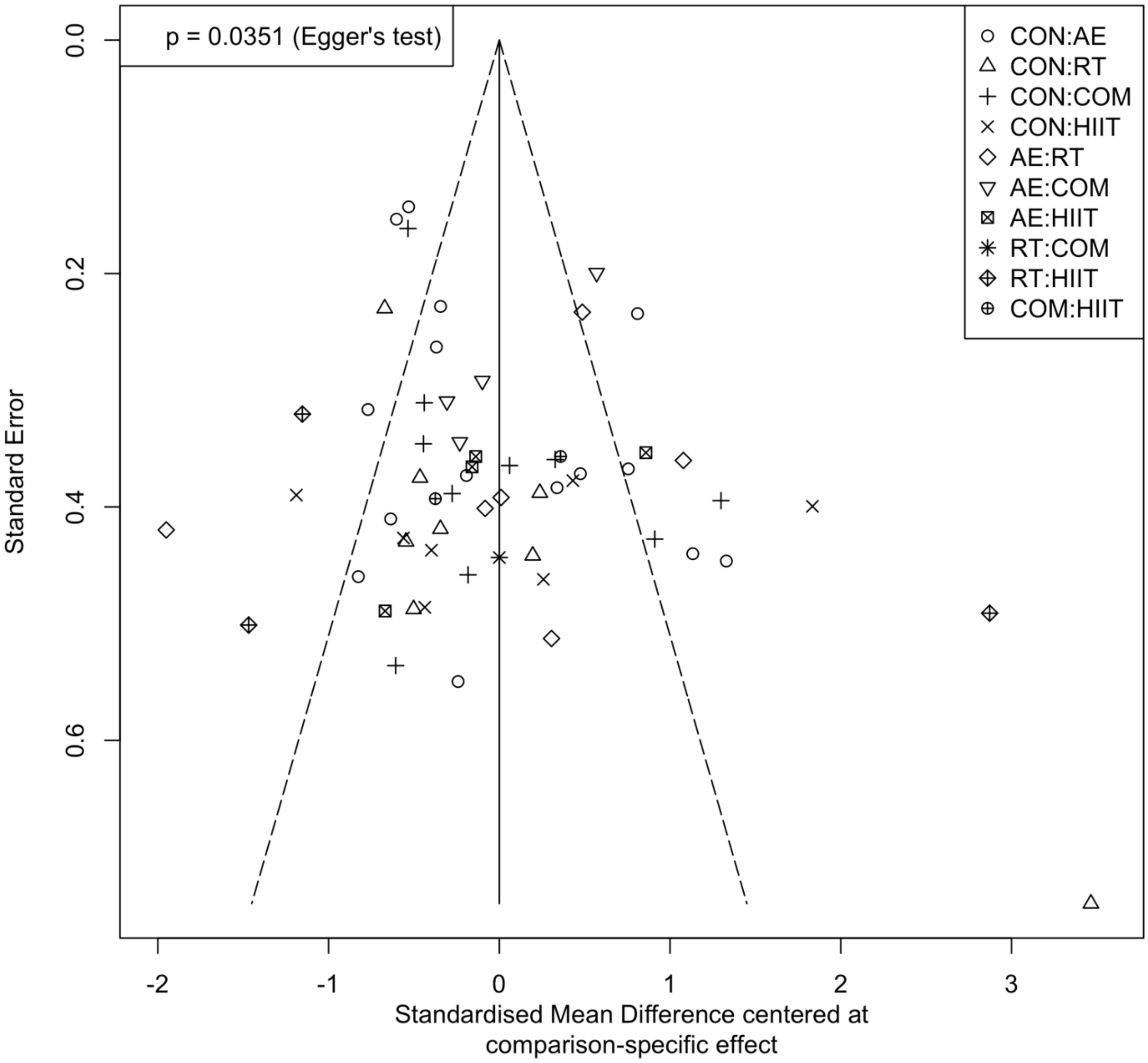

Figure 2. Funnel plot.

Note: AE: aerobic exercise, RT: resistance exercise, COM: AE combined RT, HIIT: high-intensity interval training, CON: control

Bayesian network meta-analysis

Appendix 7 Model fit summaries for included studies

1、 Adiponectin

Table 4. Model fit summaries for included studies.

Model	DIC	pD	Residual Deviance	SD (95%CrI)
FE Model	421.31	3.98	417.33	-
RE Model	111.80	50.80	61	1.02(0.81,1.29)
RE UME	112.14	51.47	60.67	1.04(0.82,1.33)

Note: CrI, credible interval. Abbreviations: DIC, deviance information criterion; FE, fixed effects; pD, number of effective parameters; RE, random effects; UME, unrelated mean effects.

2、Leptin

Table 5. Model fit summaries for included studies.

Model	DIC	pD	Residual Deviance	SD (95%CrI)
FE Model	257.81	4.01	253.80	-
RE Model	97.11	41.83	55.28	0.83(0.62,1.09)
RE UME	96.29	42.09	54.20	0.80(0.59,1.06)

Note: CrI, credible interval. Abbreviations: DIC, deviance information criterion; FE, fixed effects; pD, number of effective parameters; RE, random effects; UME, unrelated mean effects.

Appendix 8 MCMC Convergence Diagnostics

MCMC Convergence Diagnostic Plots for Each Parameter of Adiponectin and Leptin

Figure 3 and Figure 4 display the MCMC convergence diagnostic plots for each parameter of Adiponectin and Leptin, respectively. Each subplot corresponds to the variation of the shrink factor for parameters d.AE.COM, d.AE.CON, d.AE.HIIT, d.AE.RT, and sd.d with respect to the iteration number. The horizontal axis represents the final iteration number of the Markov chain (ranging from 10,000 to 50,000), while the vertical axis represents the shrink factor (on a logarithmic scale). The orange solid line denotes the median of the shrink factor, and the blue dashed line indicates the 97.5th percentile of the shrink factor. The vertical dashed line (at iteration 10,000) marks the boundary between the burn-in period and the formal sampling phase. It can be observed that the shrink factors for all parameters gradually approach 1 in the later iterations, indicating that the MCMC chain is well mixed after the burn-in period, stable estimates for the model parameters have been obtained, and convergence is satisfactory.

(1)、Adiponectin

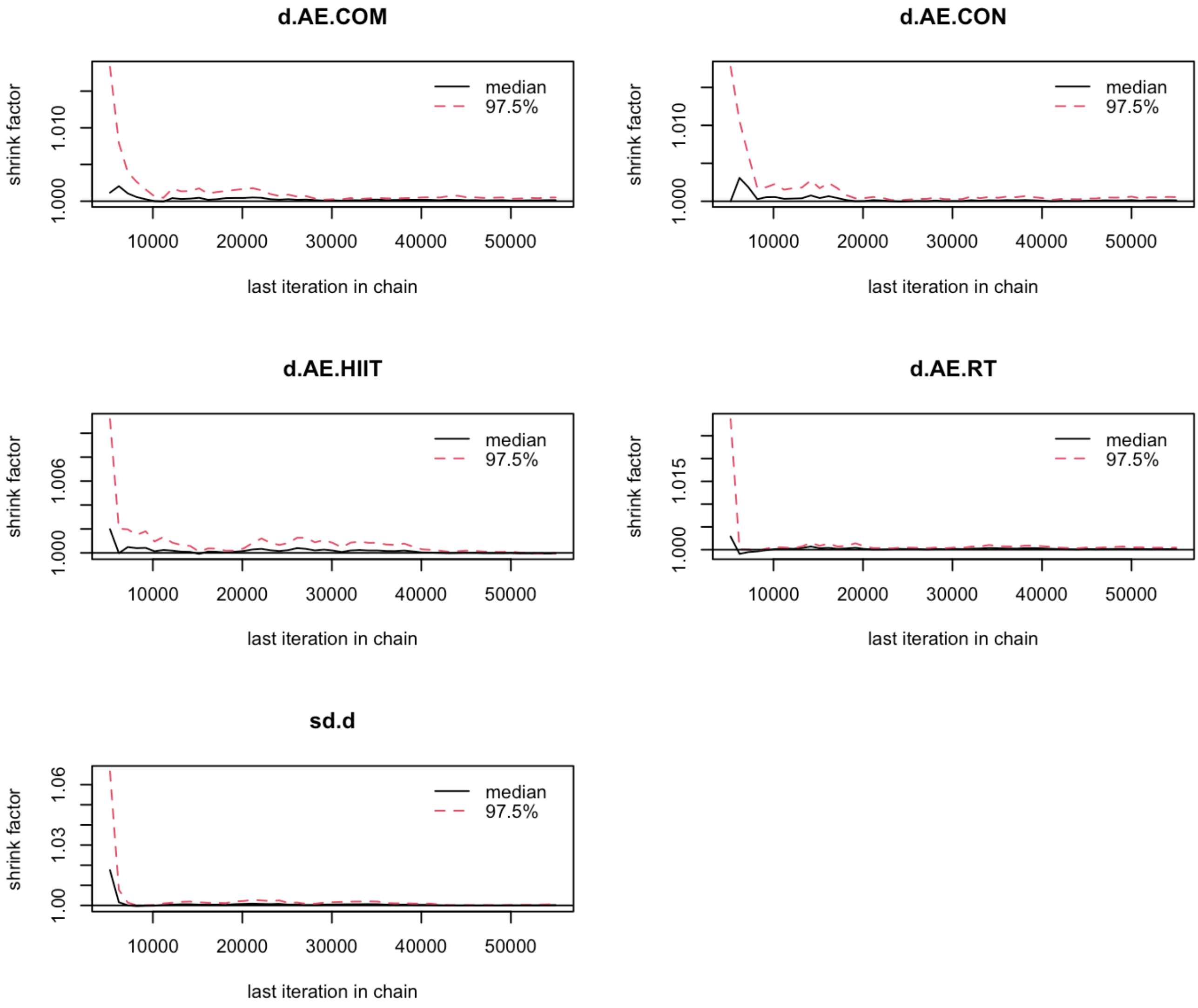

Figure 3 MCMC convergence diagnostic plots for each parameter of Adiponectin

Note: AE: aerobic exercise, RT: resistance exercise, COM: AE combined RT, HIIT: high-intensity interval training, CON: control

(2)、Leptin

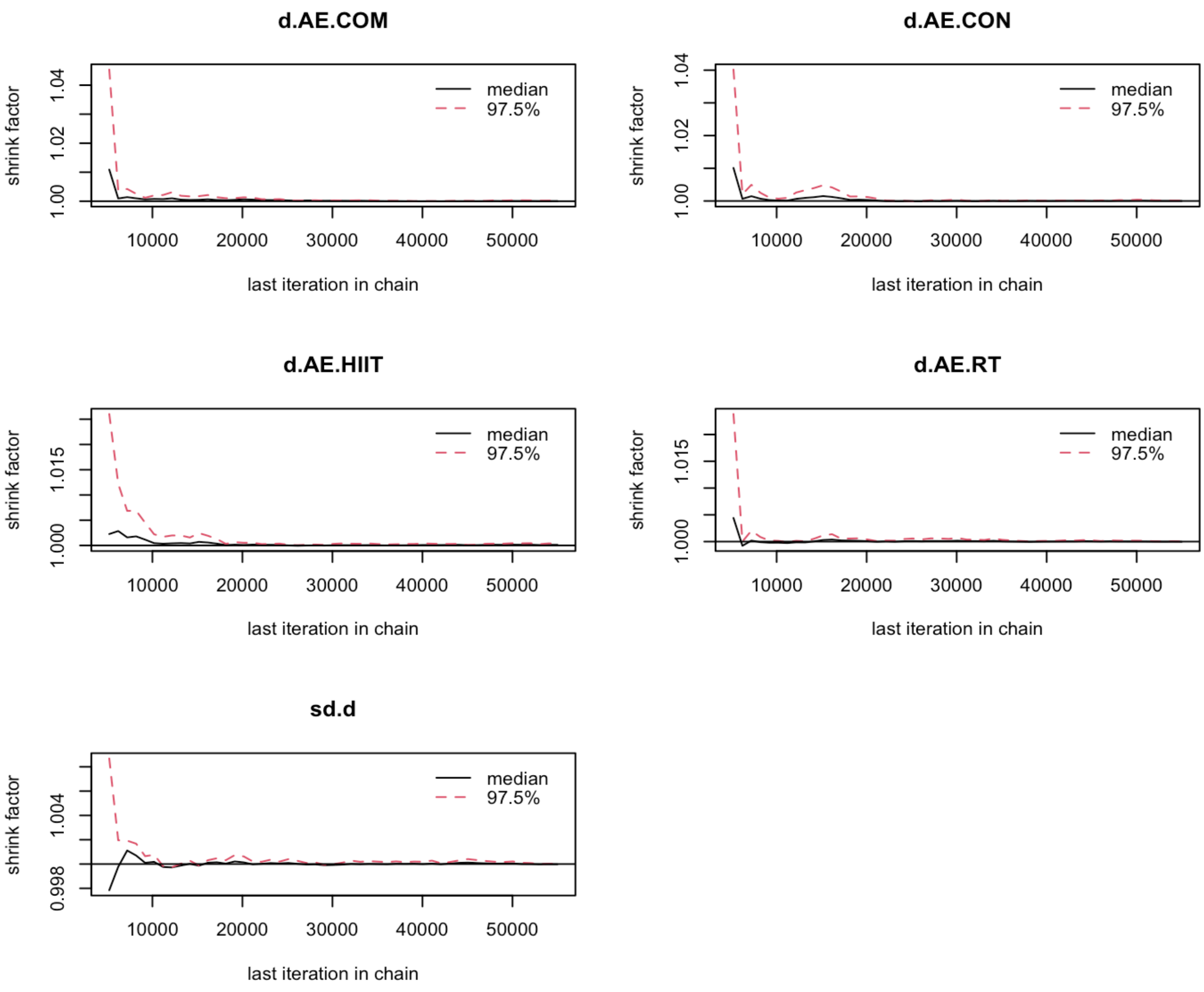

Figure 4 MCMC convergence diagnostic plots for each parameter of Leptin

Note: AE: aerobic exercise, RT: resistance exercise, COM: AE combined RT, HIIT: high-intensity interval training, CON: control

Appendix 9 MCMC Sampling Trajectories and Posterior Distributions

MCMC Sampling Trajectories and Posterior Distributions for the Parameters of Adiponectin and Leptin

Figure 5 and Figure 6 respectively display the MCMC sampling trajectories (left column) and posterior distributions (right column) for the parameters d.AE.COM, d.AE.CON, d.AE.HIIT, d.AE.RT, and sd.d of Adiponectin and Leptin. The horizontal axis of the sampling trajectories represents the iteration number (ranging from 10,000 to 50,000), while the vertical axis represents the parameter values; different colored trajectories represent independent Markov chains, which are used to assess inter-chain mixing and stability. The horizontal axis of the posterior distribution density plots represents the parameter values, while the vertical axis represents the density, with the bandwidth used in kernel density estimation and the sample size (N = 12,500) indicated.

It can be observed from the sampling trajectories that, after the burn-in period, all parameters exhibit no obvious trends or drifts and fluctuate randomly around a stable region, indicating that the MCMC chains have converged and are well mixed. The posterior distributions are predominantly unimodal, relatively symmetric, and concentrated, suggesting that the parameter estimates are stable and exhibit low uncertainty. Taken together, the results shown in these figures support the reliability and convergence of the model for the parameters of Adiponectin and Leptin.

(1)、Adiponectin

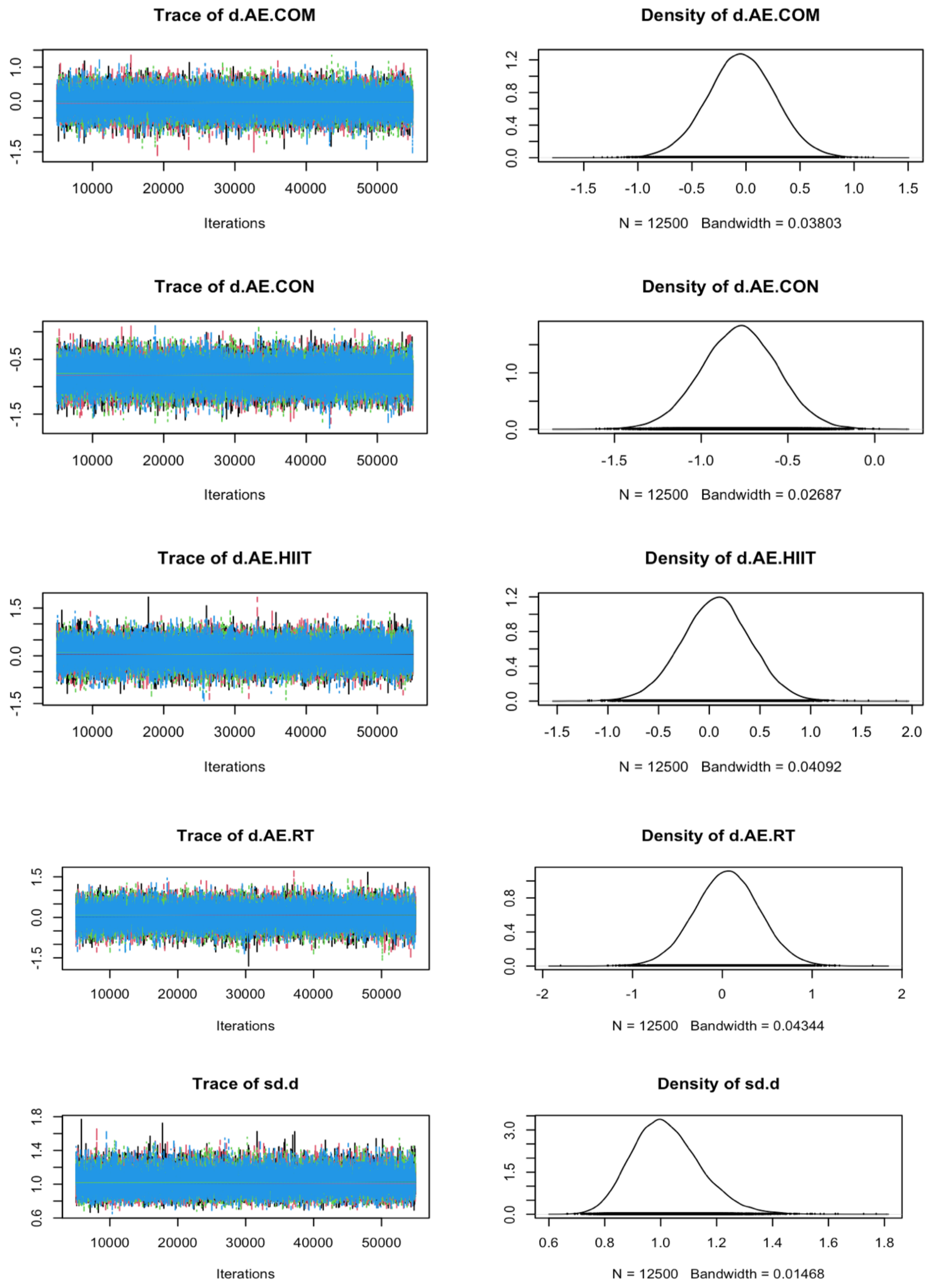

Figure 5 MCMC Sampling Trajectories and Posterior Distributions for the Parameters of Adiponectin

AE: aerobic exercise, RT: resistance exercise, COM: AE combined RT, HIIT: high-intensity interval training, CON: control

(2)、Leptin

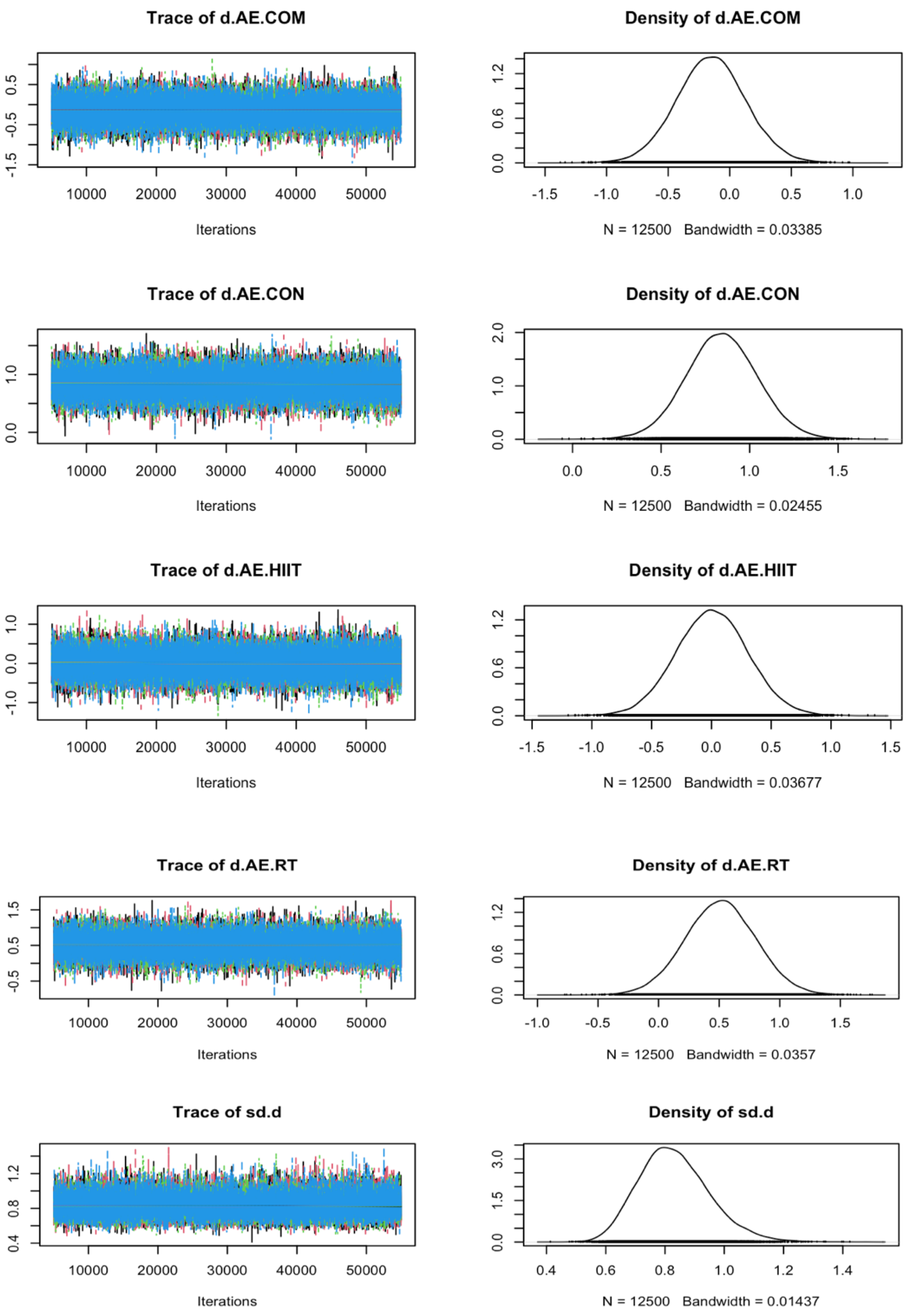

Figure 5 MCMC Sampling Trajectories and Posterior Distributions for the Parameters of Leptin
AE: aerobic exercise, RT: resistance exercise, COM: AE combined RT, HIIT: high-intensity interval training, CON: control

Appendix 10 Node-split for all studies

1、Adiponectin

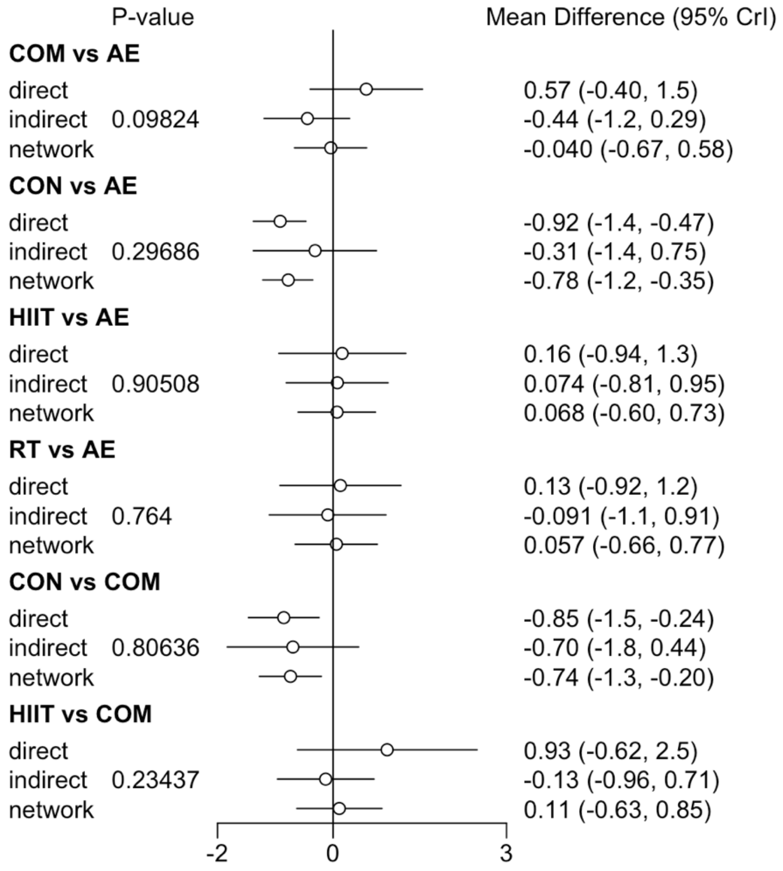

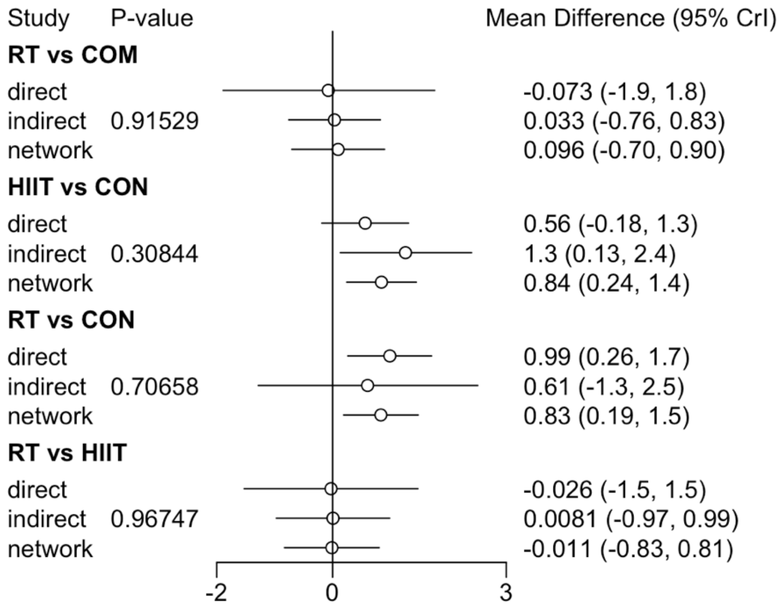

Figure 6 Node-split for all studies

Note: CrI, credible interval,AE: aerobic exercise, RT: resistance exercise, COM: AE combined RT, HIIT: high-intensity interval training, CON: control

2、Leptin

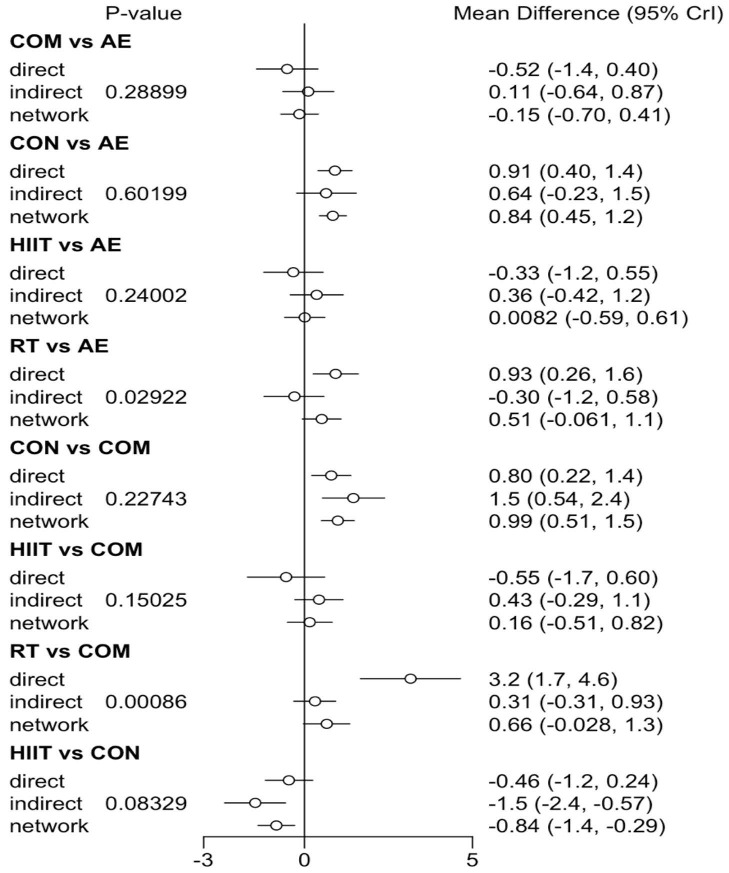

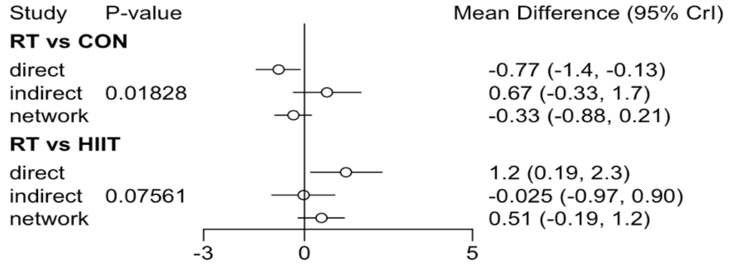

Figure 7 Node-split for all studies

Note: CrI, credible interval,AE: aerobic exercise, RT: resistance exercise, COM: AE combined RT, HIIT: high-intensity interval training, CON: control

Appendix 11 SUCRA table for all studies

1、Adiponectin

Table 6. SUCRA table for all studies

Treatment	Rank 1	Rank 2	Rank 3	Rank 4	Rank 5	SUCRA
AE	0.1713	0.29146	0.31396	0.22298	0.0003	60%
COM	0.17626	0.22216	0.26708	0.33036	0.00414	56%
CON	0.000	0.000	0.00032	0.013	0.98668	0.34%
HIIT	0.32842	0.25518	0.21322	0.20004	0.00314	68%
RT	0.32402	0.2312	0.20542	0.23362	0.00574	66%

Note: AE: aerobic exercise, RT: resistance exercise, COM: AE combined RT, HIIT: high-intensity interval training, CON: control

2、Leptin

Table 7. SUCRA table for all studies

Treatment	Rank 1	Rank 2	Rank 3	Rank 4	Rank 5	SUCRA
AE	0.1917	0.40342	0.3777	0.0271	0.00008	69%
COM	0.5534	0.27308	0.1532	0.02028	0.00004	84%
CON	0	0	0.00058	0.11162	0.8878	2.80%
HIIT	0.24906	0.30202	0.38502	0.0623	0.0016	68%
RT	0.00584	0.02148	0.0835	0.7787	0.11048	25%

Note: AE: aerobic exercise, RT: resistance exercise, COM: AE combined RT, HIIT: high-intensity interval training, CON: control

Appendix 12 GRADE Evidence Quality Assessment for adiponectin and Leptin

1. Summary of study limitations of the included studies.

The colours of the line indicate the summative ROB assessment of each comparison based on ROB assessment of each included studies (low ROB comparison [green], moderate ROB comparison [yellow] and high ROB comparison [red]).

(1) Adiponectin

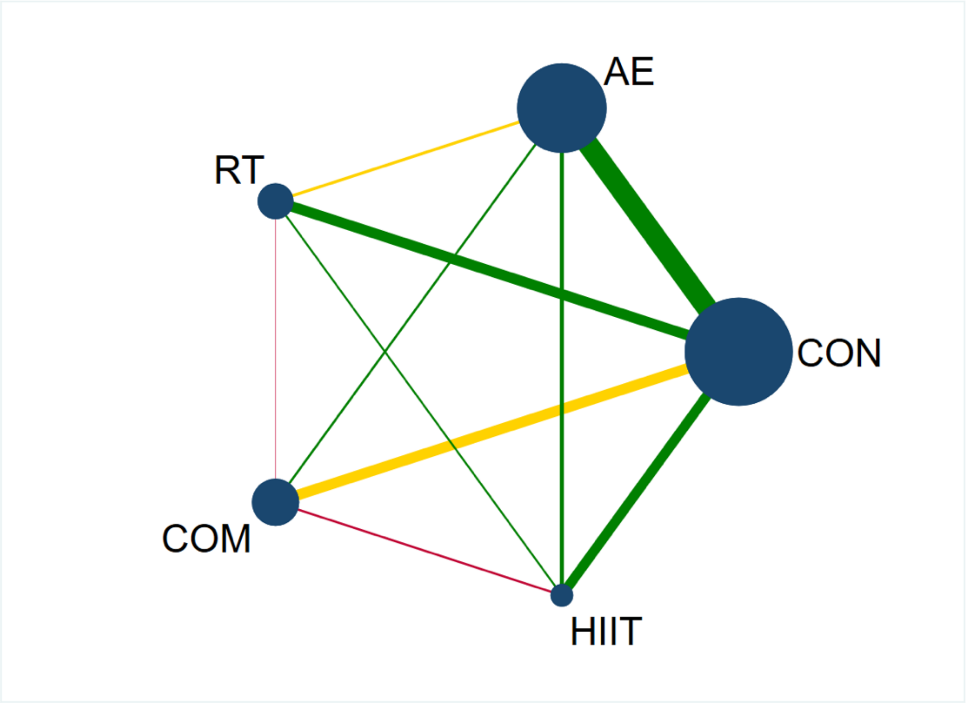

(2) Leptin

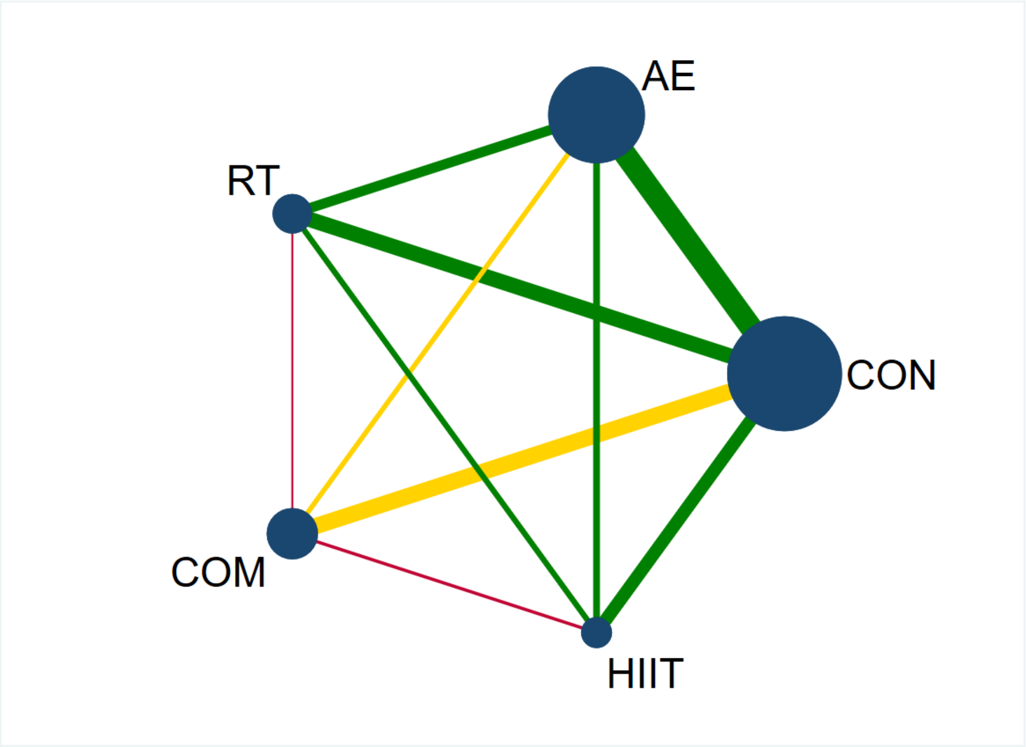

2. Contribution of ROB comparisons to each network estimate.

Based on the above assessment of ROB for each comparison and the contributions of direct and indirect comparisons to all network estimates, the following bar graphs show the percentage of low, moderate and high ROB contributions for each network estimate.

Note: A-CON, B-AE, C-RT, D-COM, E-HIIT

(1) Adiponectin

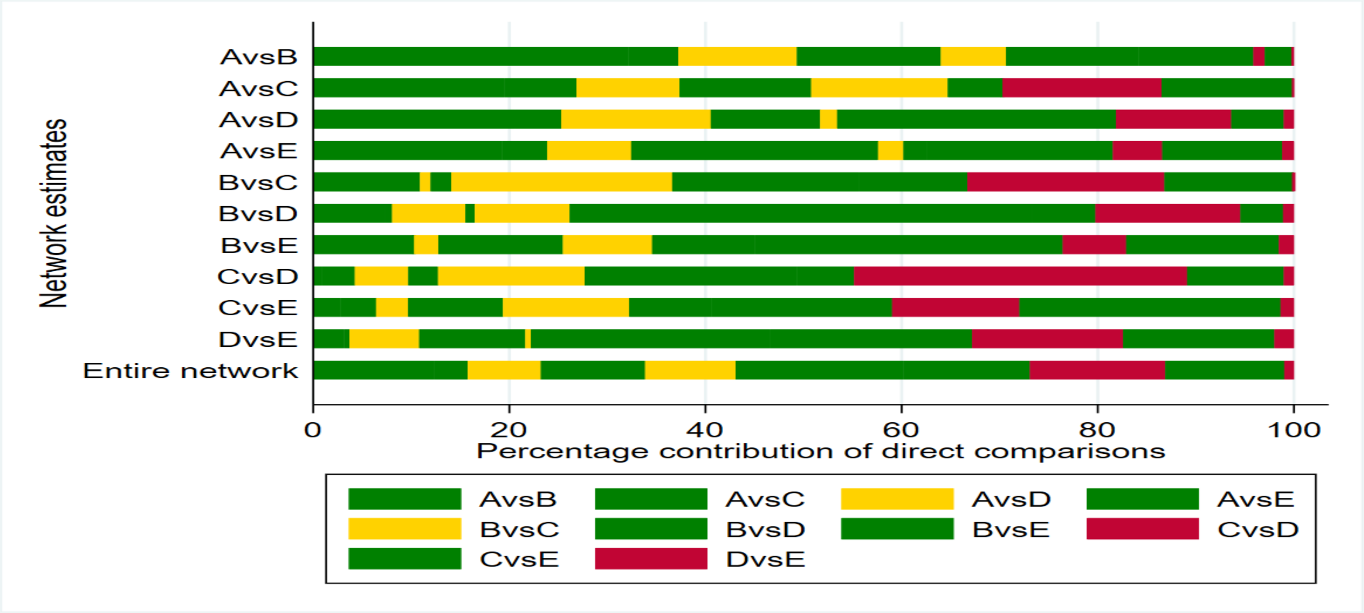

(2) Leptin

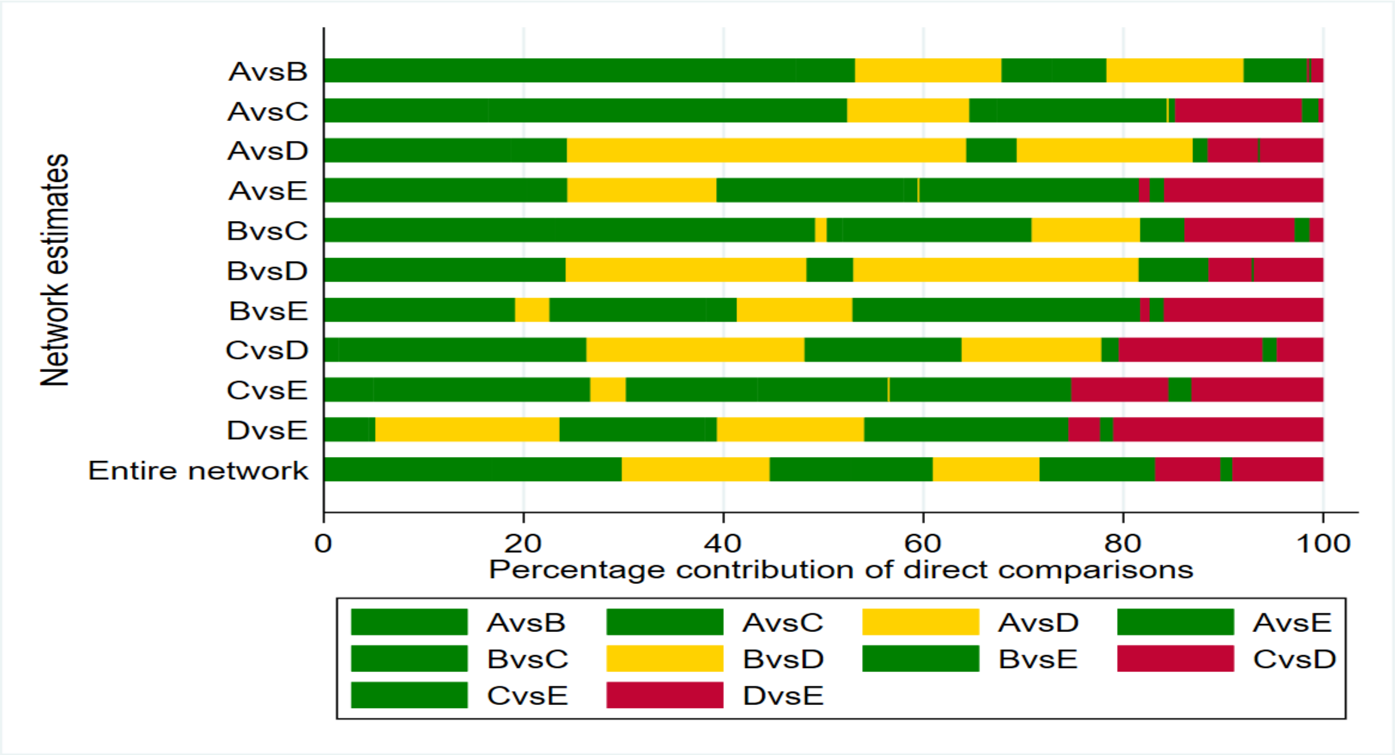

3. Table of reasons for downgrading.

Based on all the above information, we GRADE each network estimate according to the following criteria.

(1) Study limitations

Low risk (green)/unclear risk (yellow)/high risk (red) of each direct comparison was given a weight of 0/1/-2, respectively. And then the risk value of each comparison and SUCRA probability sorting result were calculated according to the contribution to ROB comparisons to each network estimate. It would be downgraded by one level when the risk value is between -1.5 and -0.6 (indicated by “Study limitations”) and two level when the risk value is less than -1.6 (indicated by “Study limitations2”).

(2) Indirectness

In the network plot, singly-connected nodes were slightly downgraded in terms of indirectness because assessing transitivity for these nodes is unclear. To put it simply, if the results of two comparisons are obtained solely through indirect comparisons rather than direct ones, the reliability of the outcome will be downgraded by one level.

(3) Inconsistency

In this study, we evaluated heterogeneity and inconsistency. The heterogeneity between each pair of direct comparisons was assessed using the I^2 statistic from pairwise comparisons (see Appendix 3). When inconsistency was observed, it was categorized as high heterogeneity (labeled as “heterogeneous”). Inconsistency between direct and indirect comparisons was evaluated using MCMC sampling trajectories, posterior distributions, and node-splitting analyses (see Appendices 7 and 8; labeled as “inconsistency”). The stability of the SUCRA probability ranking was determined based on overall inconsistency (see Appendix 7).

(4) Imprecision

Imprecision was evaluated by appropriate sample sizes, a sample size below 400 would be downgraded by one level. The total sample size in this study was over 400, so this project was not downgraded. The Imprecision of the ranking results is evaluated according to the gap between the ranking values. If the gap between the ranking values of each treatment is small, which indicating a poor stability, the intervention would be downgraded by one level.

(5) Publication bias

We assessed potential publication bias by examining the symmetry of pairwise comparisons in the comparison-adjusted funnel plot and performing Egger’s test. The comparison-adjusted funnel plot (Appendix 4) indicated the presence of overall publication bias for both adiponectin and leptin outcomes.

Below are the GRADE assessments for adiponectin and leptin.

(1) Adiponectin

Comparison	Nature of the evidence	Confidence	Downgrading due to
CON vs AE	Mixed	**Low**	**Heterogeneity, Publication bias**
CON vs RT		**Moderate**	**Heterogeneity**
CON vs COM		**Low**	**Heterogeneity, Publication bias**
CON vs HIIT		**Moderate**	**Heterogeneity**
AE vs RT		**Low**	**Study limitations, Publication bias**
AE vs COM		**Moderate**	**Inconsistency**
AE vs HIIT		**Moderate**	**Publication bias**
RT vs COM		**Moderate**	**Study limitations**
RT vs HIIT		**High**	
COM vs HIIT		**Moderate**	**Inconsistency**

**Ranking of treatments**		**Moderate**	**Heterogeneity**

(2) Leptin

Comparison	Nature of the evidence	Confidence	Downgrading due to
CON vs AE		**Moderate**	**Heterogeneity**
CON vs RT		**Low**	**Inconsistency, Publication bias**
CON vs COM		**Very low**	**Study limitations, Heterogeneity, Inconsistency**
CON vs HIIT		**Moderate**	**Heterogeneity**
AE vs RT		**Moderate**	**Publication bias**
AE vs COM	**Mixed**	**Low**	**Study limitations, Inconsistency**
AE vs HIIT		**Moderate**	**Publication bias**
RT vs COM		**Low**	**Study limitations, Publication bias**
RT vs HIIT		**Low**	**Inconsistency, Publication bias**
COM vs HIIT		**Moderate**	**Publication bias**
**Ranking of treatments**		**Moderate**	**Heterogeneity**

Dose–Response NMAs

Appendix 13 Key Assumptions of Dose-Response Network Meta-Analysis

一、Network Connectivity

Connectivity is a key assumption in NMA that, if considered insufficient (i.e., due to lack of direct comparisons), may lead to low statistical power and misleading results[62]. Our study assessed network connectivity at the motor and dose levels and did not find any evidence of network unconnectedness.

1、Adiponectin (Figure 1 and Figure 2)

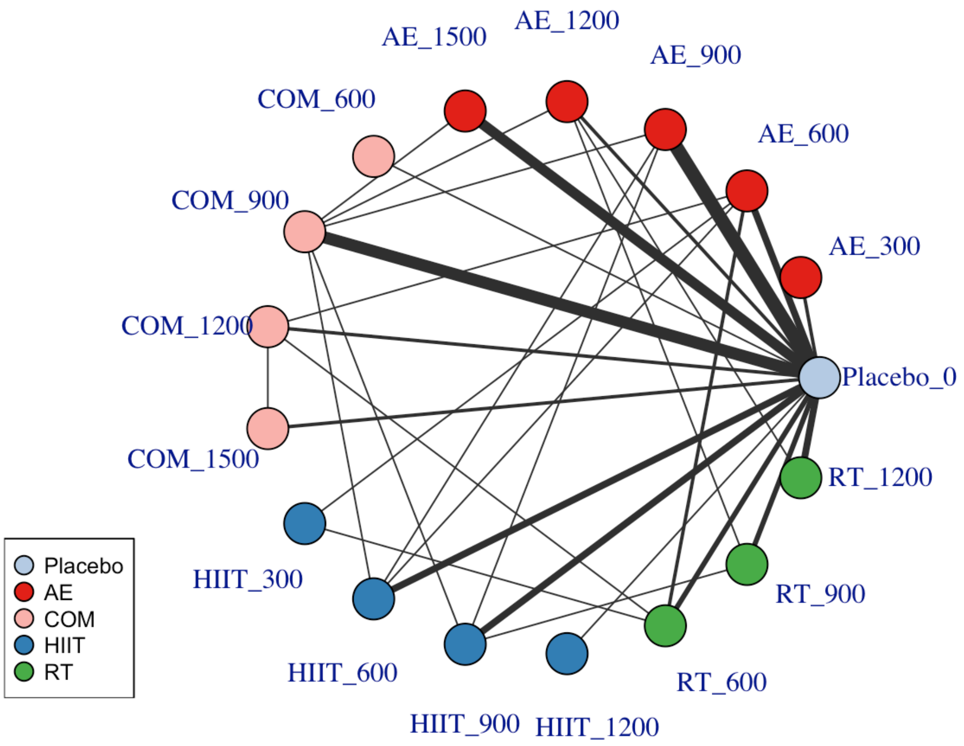

Figure 1 Treatment-level network. The first value indicates the specific intervention and the second one is the corresponding dose of that intervention. AE: Aerobic exercise; RT: Resistance training; COM: Aerobic combined with resistance training; HIIT: High-intensity interval training; Placebo: control group.

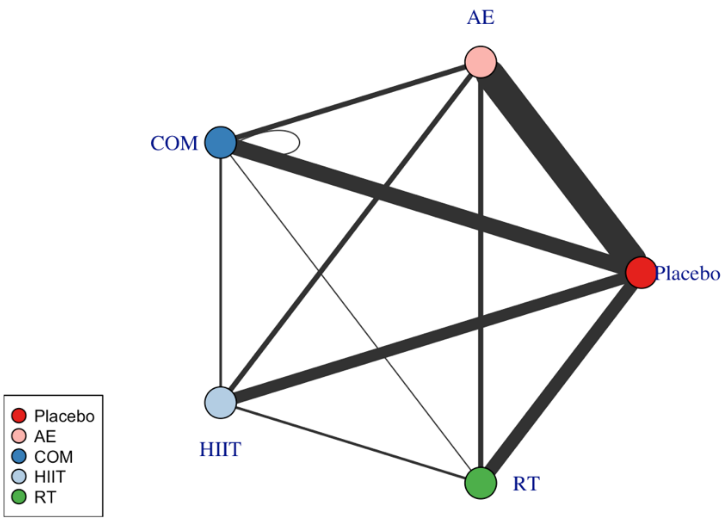

Figure 2 AE: Aerobic exercise; RT: Resistance training; COM: Aerobic combined with resistance training; HIIT: High- intensity interval training; Placebo: control group.

2、Leptin (Figure 3 and Figure 4)

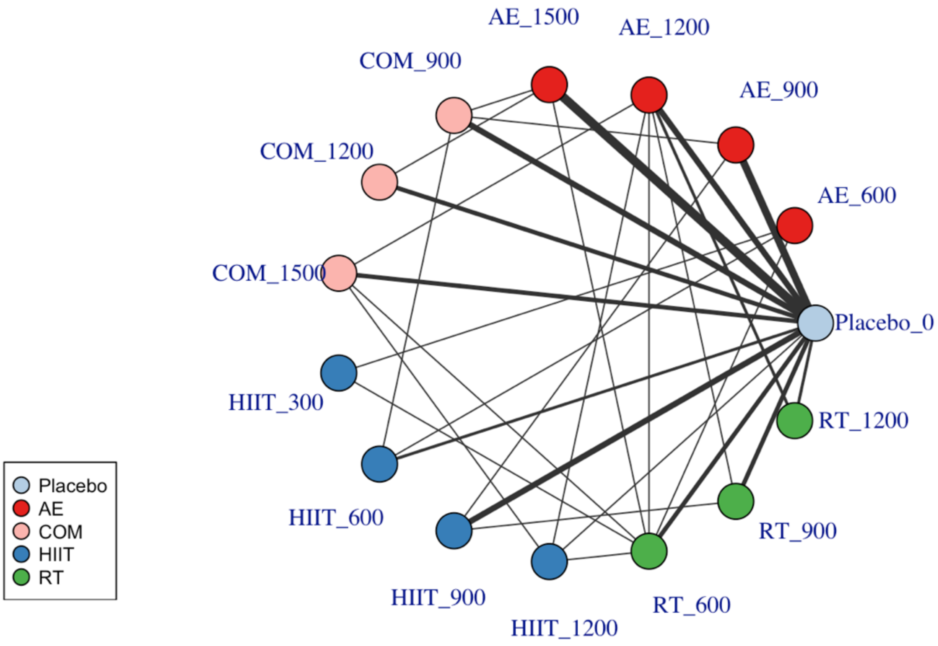

Figure 3 Treatment-level network. The first value indicates the specific intervention and the second one is the corresponding dose of that intervention. AE: Aerobic exercise; RT: Resistance training; COM: Aerobic combined with resistance training; HIIT: High- intensity interval training; Placebo: control group.

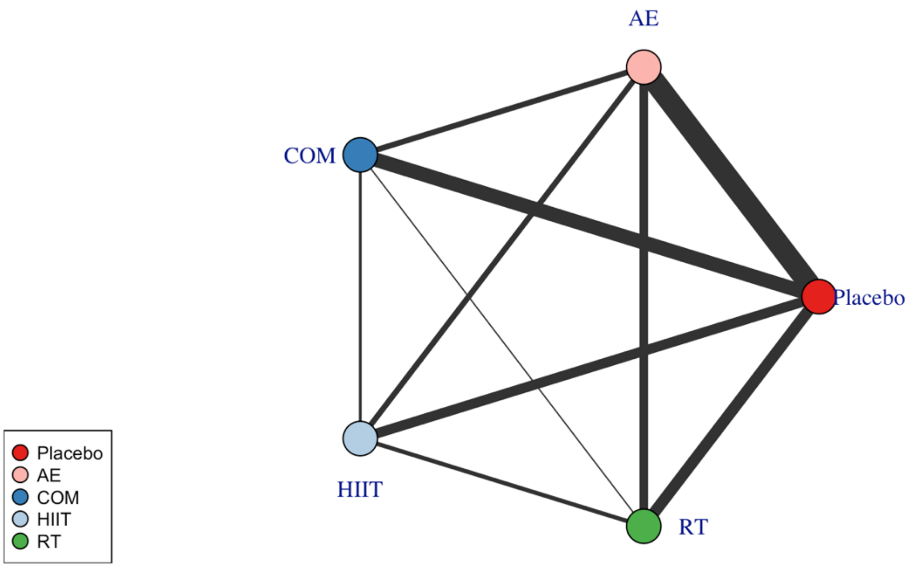

Figure 4 AE: Aerobic exercise; RT: Resistance training; COM: Aerobic combined with resistance training; HIIT: High- intensity interval training; Placebo: control group.

二、Data consistency

We performed a consistency analysis of the data by comparing the consistency of the network (i.e., network effect size) with the unrelated mean effects (UME) model (i.e., pairwise effect sizes). In practice, we checked whether the bias, the number of estimated parameters in the network, and the Deviance Information Criterion (DIC) metric were similar for both models, indicating a good fit[63]. The comparison of these parameters showed a good agreement between the different models.

1、Adiponectin (Table 1)

Table 1 Consistent and UME models fit comparison

Model	pD	Deviance	DIC	SD
Consistent	119	264.74	376.7	1.44
UME	113.1	263.82	375.8	1.61

2、Leptin (Table 2)

Table 2 Consistent and UME models fit comparison

**Model**	**pD**	**Deviance**	**DIC**	**SD**
Consistent	90.8	263.46	349.7	1.02
UME	90.2	262.97	347.1	0.93

三、Network transitivity

NMAs are based on the assumption of indirect/mixed comparisons, which implies that estimates of treatment effects from direct and indirect evidence are consistent but with the usual variation of meta-analyses under a random effects model[64]. This assumption is equivalent to heterogeneity in a "standard" meta-analysis[65]. Following a previous proposal[66], anomalies were assessed at a deeper network level (i.e., at the treatment level). We assessed the span by the MBNMA node-splitting method. This method splits the contribution of a specific treatment contrast into direct and indirect evidence and compares them[67]. Similar effects indicate good span. Table 3 and Figure 5, Table 4 and Figure 6 presents the results for transitivity in this meta-analysis.

1、Adiponectin (Table 3 and Figure 5)

Table 3 Node-splitting analysis of consistency

Comparison	p-value	Median	2.5%	97.50%
COM_1500 vs COM_1200	0.171			
-> direct		0.125	-3.182	3.546
-> indirect		0.219	-0.027	0.478
-> MBNMA		0.222	-0.019	0.461
HIIT_900 vs COM_900	0.564			
-> direct		0.056	-3.429	3.436
-> indirect		0.277	-0.976	1.535
-> MBNMA		0.226	-1.014	1.439
HIIT_600 vs COM_900	0.235			
-> direct		1.997	-1.356	5.322
-> indirect		-0.232	-1.199	0.728
-> MBNMA		-0.068	-1.046	0.844
COM_900 vs AE_1500	0.374			
-> direct		0.935	-2.329	4.183
-> indirect		-0.625	-1.807	0.5
-> MBNMA		-0.441	-1.601	0.607
COM_900 vs AE_1200	0.428			
-> direct		0.546	-2.790	3.966
-> indirect		-0.289	-1.265	0.696
-> MBNMA		-0.222	-1.239	0.705
RT_600 vs AE_600	0.519			
-> direct		0.333	-1.607	2.347
-> indirect		0.509	-0.191	1.18
-> MBNMA		0.556	-0.116	1.229
HIIT_600 vs AE_600	0.389			
-> direct		-0.204	-3.527	3.031
-> indirect		0.169	-0.627	0.952
-> MBNMA		0.148	-0.652	0.936
HIIT_300 vs AE_600	0.280			
-> direct		-0.046	-3.394	3.322
-> indirect		-0.150	-0.638	0.329
-> MBNMA		-0.147	-0.644	0.366
RT_1200 vs Placebo_0	0.384			

-> direct		2.533	1.075	3.969
-> indirect		1.020	-0.811	2.853
-> MBNMA		2.010	0.882	3.201
RT_900 vs Placebo_0	0.262			
-> direct		0.248	-1.532	1.984
-> indirect		1.795	0.806	2.8
-> MBNMA		1.507	0.661	2.401
HIIT_1200 vs Placebo_0	0.611			
-> direct		0.784	-2.773	4.197
-> indirect		1.250	-0.266	2.828
-> MBNMA		1.188	-0.277	2.605
COM_600 vs Placebo_0	0.283			
-> direct		0.317	-2.961	3.671
-> indirect		0.440	-0.064	0.944
-> MBNMA		0.443	-0.039	0.921
AE_900 vs Placebo_0	0.576			
-> direct		1.009	-0.165	2.21
-> indirect		0.578	-0.061	1.191
-> MBNMA		0.671	0.110	1.237
AE_300 vs Placebo_0	0.000			
-> direct		4.834	2.739	6.921
-> indirect		0.193	0.037	0.353
-> MBNMA		0.224	0.037	0.412

Note: CrI, credible interval; AE: Aerobic exercise; RT: Resistance training; COM: Aerobic combined with resistance training; HIIT: High- intensity interval training; Placebo: control group.

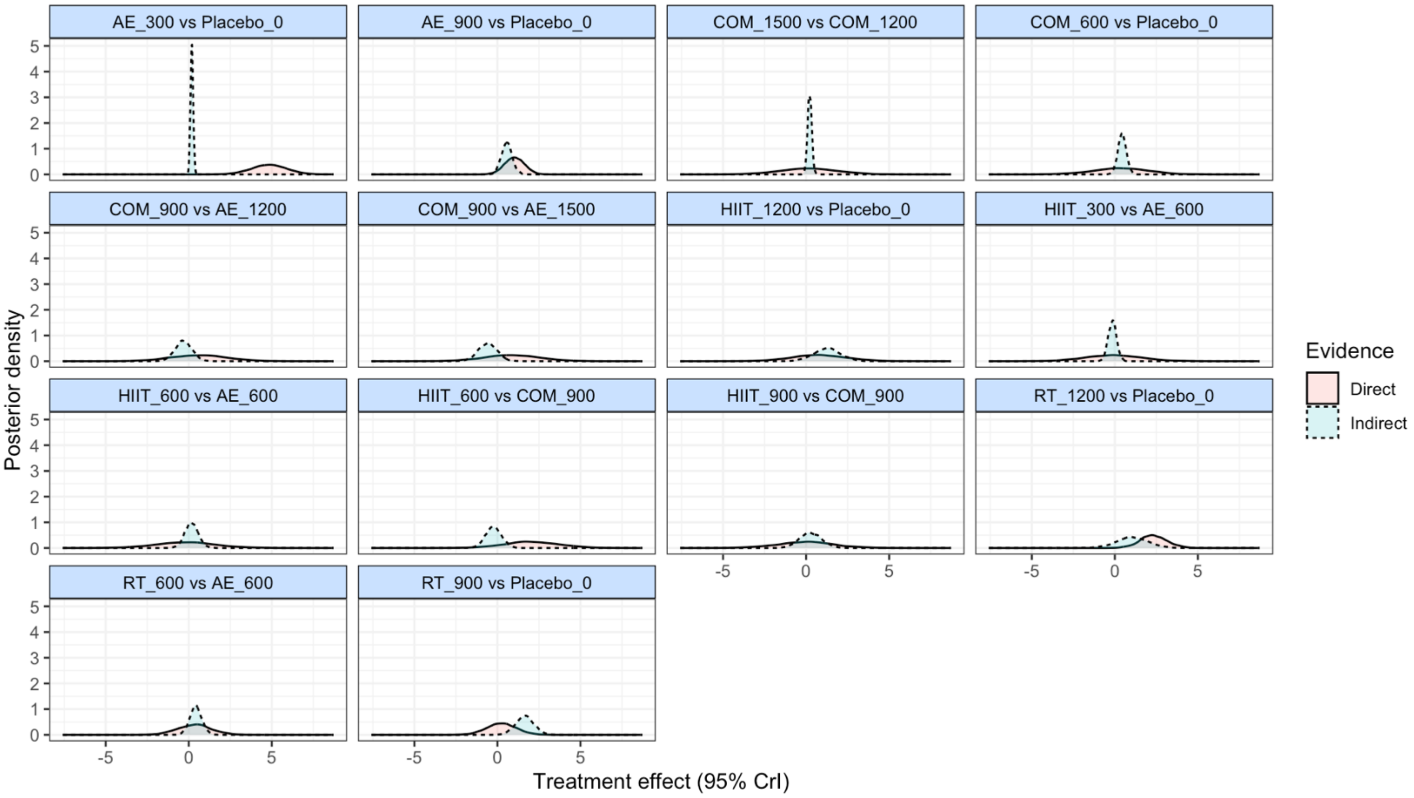

Figure 5 Node-splitting analysis (density plot). The value of title is the corresponding dose of that agent. CrI, credible interval. AE: Aerobic exercise; RT: Resistance training; COM: Aerobic combined with resistance training; HIIT: High-

intensity interval training; Placebo: control group

2、Leptin (Table 4 and Figure 6)

Table 4 Node-splitting analysis of consistency

Comparison	p-value	Median	2.5%	97.50%
HIIT_600 vs COM_900	0.415			
-> direct		-0.184	-2.216	2.003
-> indirect		0.191	-0.384	0.754
-> MBNMA		0.181	-0.378	0.747
COM_1200 vs AE_1500	0.508			
-> direct		-0.422	-2.486	1.709
-> indirect		0.080	-0.662	0.837
-> MBNMA		0.010	-0.695	0.68
COM_900 vs AE_1500	0.202			
-> direct		-1.086	-3.046	0.843
-> indirect		0.425	-0.206	1.054
-> MBNMA		0.276	-0.334	0.884
RT_1200 vs AE_1200	0.392			
-> direct		0.827	-0.554	2.217
-> indirect		-0.192	-1.211	0.777
-> MBNMA		0.104	-0.707	0.913
RT_600 vs AE_1200	0.009			
-> direct		2.970	1.434	4.519
-> indirect		0.305	-0.154	0.772
-> MBNMA		0.493	-0.020	0.99
HIIT_1200 vs AE_1200	0.378			
-> direct		-1.326	-3.134	0.506
-> indirect		-0.076	-1.114	0.936
-> MBNMA		-0.390	-1.288	0.476
COM_1500 vs AE_1200	0.559			
-> direct		-0.320	-2.444	1.827
-> indirect		-0.507	-1.299	0.31
-> MBNMA		-0.482	-1.225	0.233
RT_600 vs AE_600	0.405			
-> direct		-0.067	-1.905	1.756
-> indirect		0.056	-0.368	0.489
-> MBNMA		0.052	-0.353	0.457
HIIT_300 vs AE_600	0.258			
-> direct		-0.195	-2.205	1.815
-> indirect		0.129	-0.157	0.419
-> MBNMA		0.120	-0.155	0.394
RT_900 vs Placebo_0	0.634			
-> direct		-0.236	-1.317	0.871
-> indirect		-0.644	-1.393	0.117
-> MBNMA		-0.573	-1.156	0.05
HIIT_900 vs Placebo_0	0.356			

-> direct		-0.439	-1.455	0.556
-> indirect		-1.314	-2.118	-0.473
-> MBNMA		-0.946	-1.571	-0.322
AE_900 vs Placebo_0	0.555			
-> direct		-0.782	-1.657	0.119
-> indirect		-0.644	-0.988	-0.285
-> MBNMA		-0.655	-0.975	-0.332

Note: CrI, credible interval; AE: Aerobic exercise; RT: Resistance training; COM: Aerobic combined with resistance training; HIIT: High- intensity interval training; Placebo: control group.

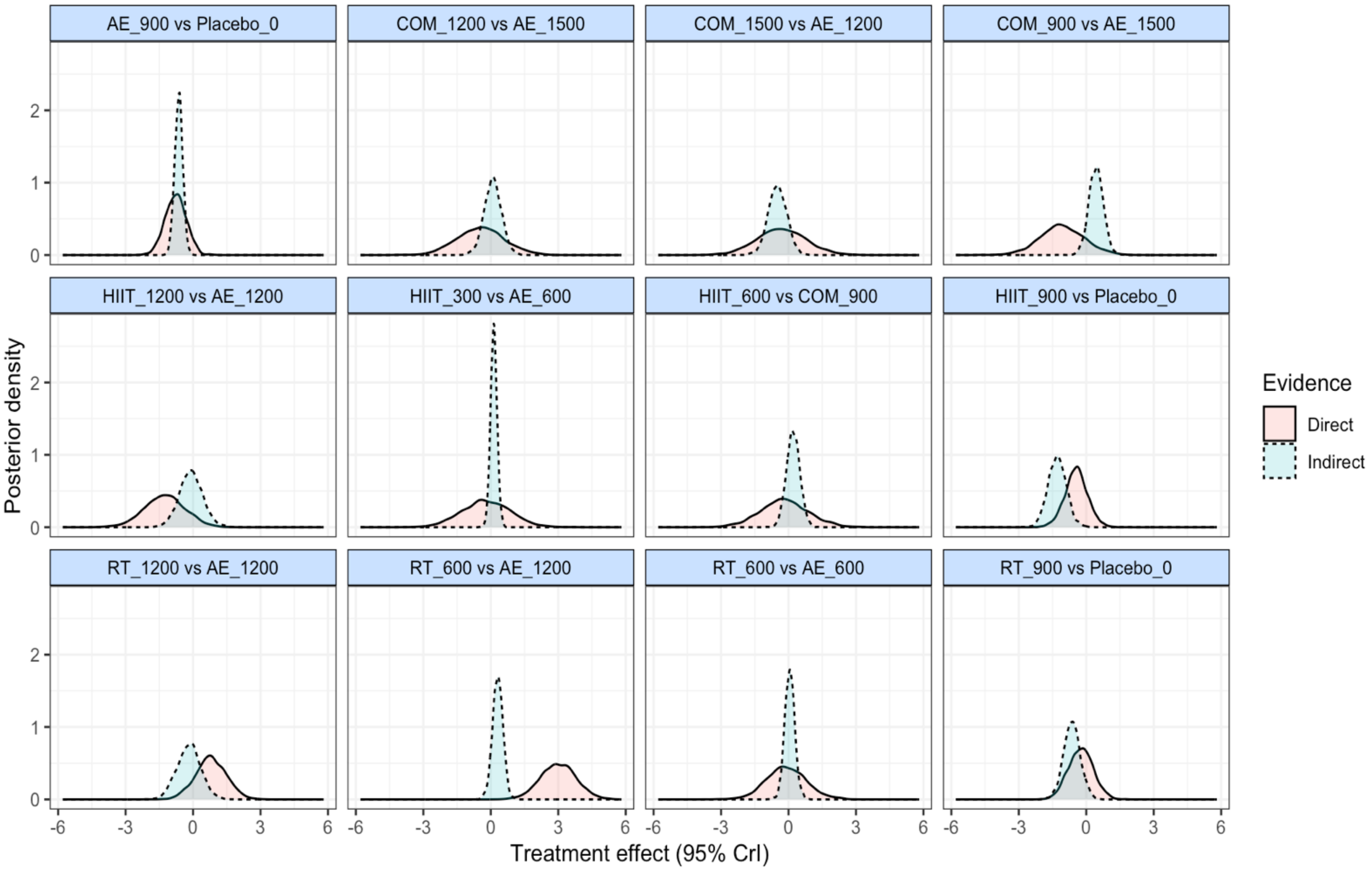

Figure 6 Node-splitting analysis (density plot). The value of title is the corresponding dose of that agent. CrI, credible interval. AE: Aerobic exercise; RT: Resistance training; COM: Aerobic combined with resistance training; HIIT: High-intensity interval training; Placebo: control group

Appendix 14 Model Selection for Dose-Response Analysis

1 Non-linear functions and models fit comparison

A meta-analysis (i.e., a "split" NMA) of the different doses of physical activity as separate and unrelated treatments were performed. This step helps determine which function is more appropriate for the data and should be used in a model-based network meta-analysis (MBNMA)[68]. **Figure 7 and Figure 8, Figure 9 and Figure 10** show the different responses of each dose to overall and different types of exercise, respectively (SMD).

(1) Adiponectin (Figure 7 and Figure 8)

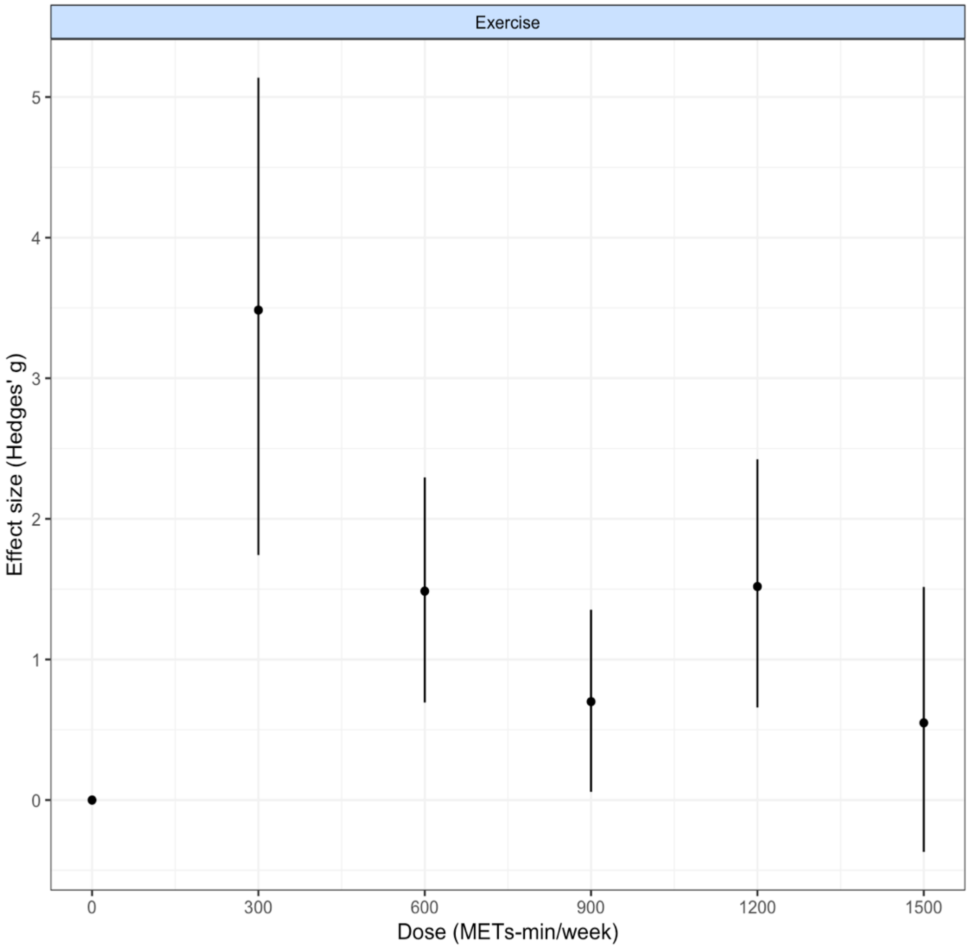

Figure 7 “Split” NMA of overall exercise.

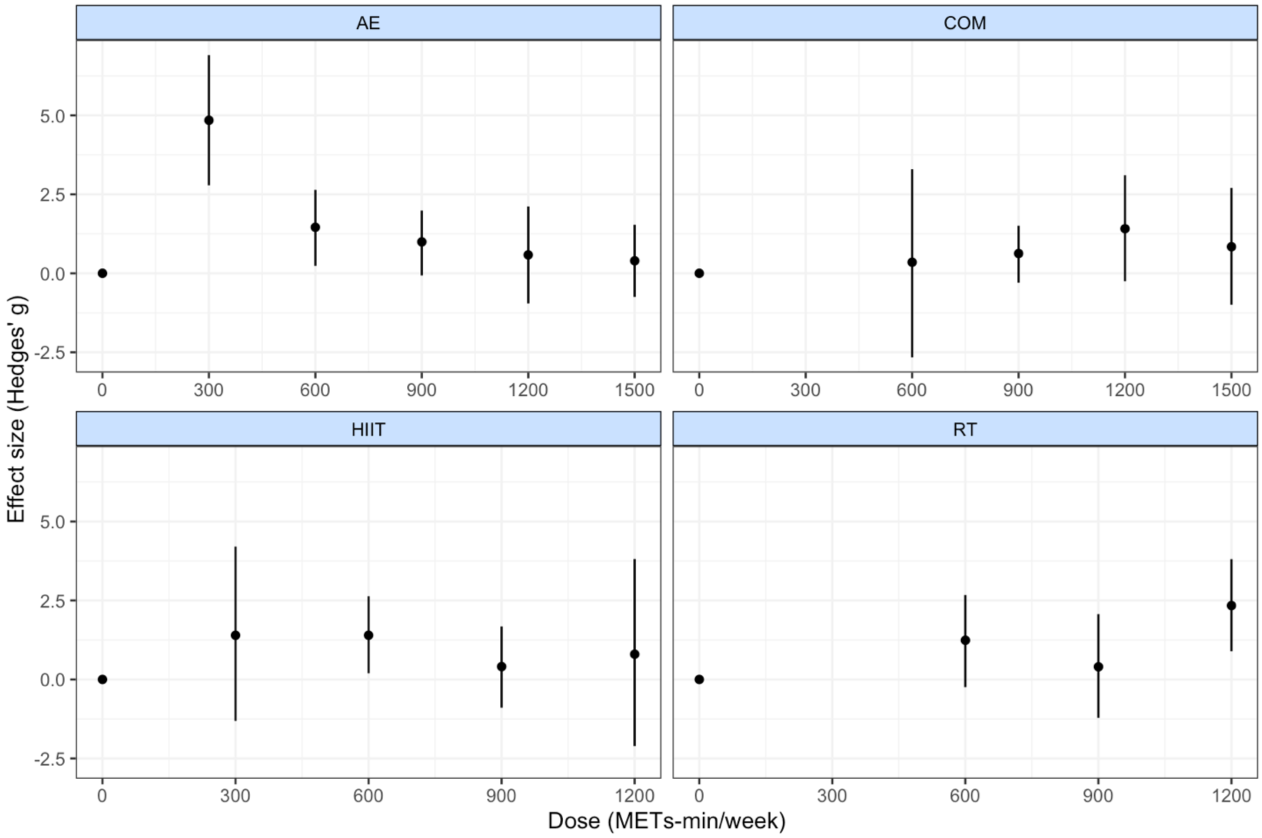

Figure 8 “Split” NMA of different exercise agents. AE: Aerobic exercise; RT: Resistance training; COM: Aerobic combined with resistance training; HIIT: High- intensity interval training

(2) Leptin (Figure 9 and Figure 10)

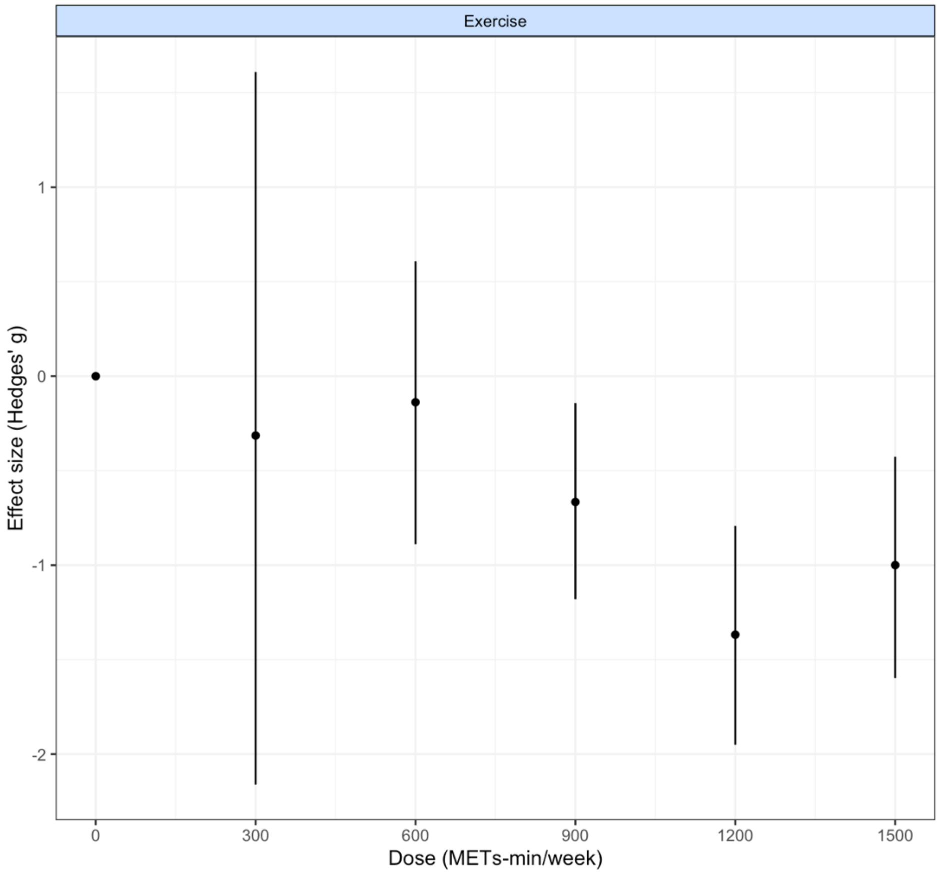

Figure 9 "Split" NMA of overall exercise

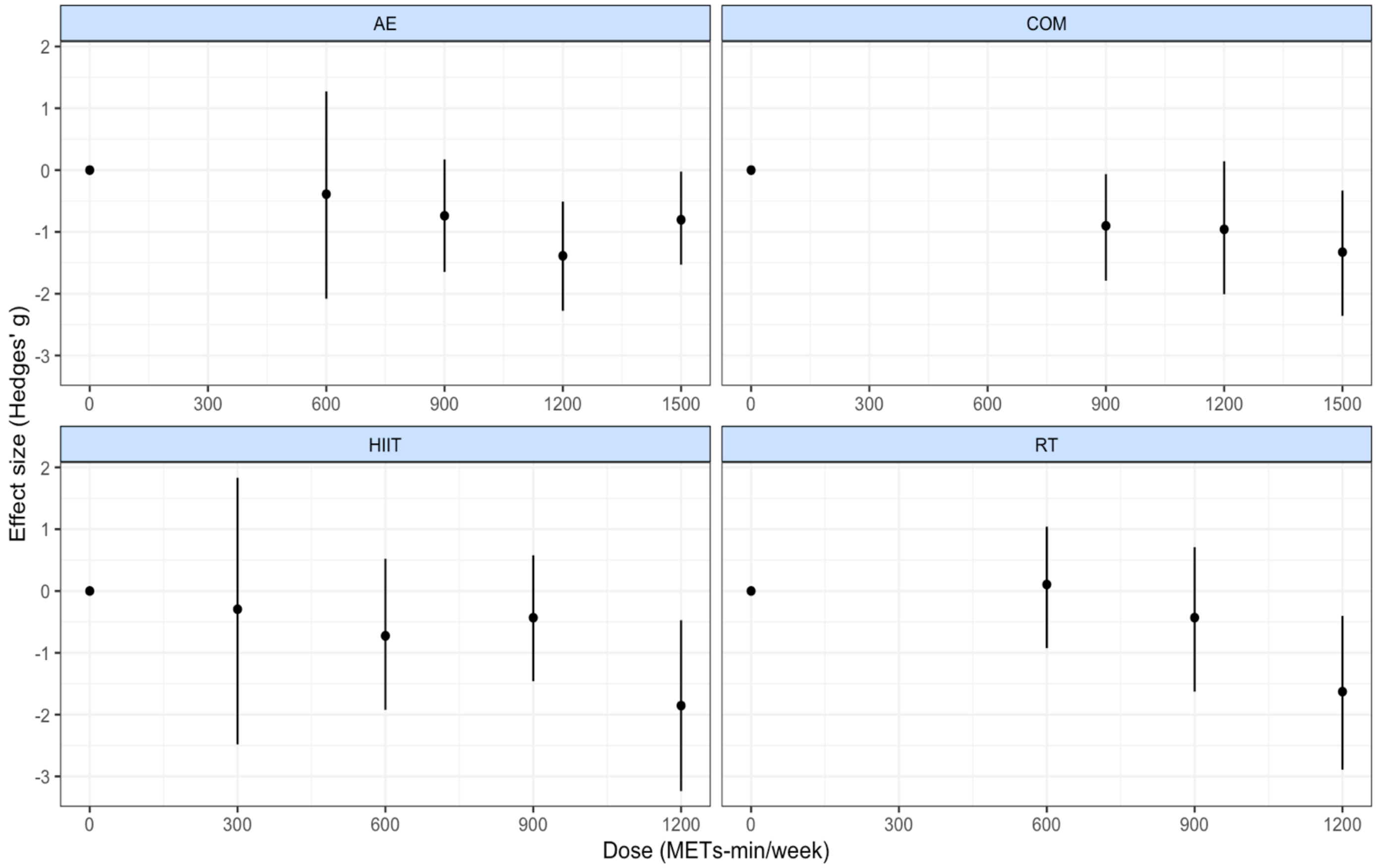

Figure 10 "Split" NMA of different exercise agents. AE: Aerobic exercise; RT: Resistance training; COM:

Aerobic combined with resistance training; HIIT: High- intensity interval training

2 Models selection

For our data, quadratic model shows the best fit and were therefore used in subsequent analyses.

(1) Adiponectin (Table 5)

Table 5 Models fit comparison

Model	DIC	SD	Deviance	Residual deviance	pD
Emax (common treatment effects)	1574.2	NA	1517.06	860.8	60.9
Emax (random treatment effects)	375.4	1.42 (1.4, 1.81)	263.85	115	110.7
Restricted cubic spline (common treatment effects; 3 knots)	1133.4	NA	1066.50	861.7	72
Restricted cubic spline (random treatment effects; 3 knots)	375.5	1.43 (1.15, 1.81)	263.73	115.3	112.2
Quadratic (2nd degree polynomial, common treatment effects)	1434.4	NA	1374.31	861.1	60.9
Quadratic (2nd degree polynomial, random treatment effects)	374.9	1.43 (1.14, 1.82)	263.63	114.5	114.7
Non-parametric monotonically up (common treatment effects)	1518.6	NA	1458.96	NA	74.8

(2) Leptin (Table 6)

Table 6 Models fit comparison

Model	DIC	SD	Deviance	Residual deviance	pD
Emax (common treatment effects)	679.7	NA	635.06	393.6	52.3
Emax (random treatment effects)	348.3	1 (0.779, 1.32)	263.10	90.6	88.9
Restricted cubic spline (common treatment effects; 3 knots)	620.6	NA	567.85	393.5	57.3
Restricted cubic spline (random treatment effects; 3 knots)	348.4	1.01 (0.771, 1.34)	263.09	89.9	93.6
Quadratic (2nd degree polynomial, common treatment effects)	1434.4	NA	1374.31	861.1	60.9
Quadratic (2nd degree polynomial, random treatment effects)	348	1 (0.774, 1.35)	262.74	90.5	90.9
Non-parametric monotonically up (common treatment effects)	1003.3	NA	961.95	NA	64.3

In addition to the model fit index, a deviation plot showing the contribution of each data point to the residuals can also help to confirm the robustness of the model selection. The contribution of each data point to the posterior mean bias should be around 1, which indicates a good model fit[69]. Deviation plots for overall **(Figure 11 and Figure 13)** treatment effects **(Figure 12 and Figure 14)** confirm the robustness of our model selection (i.e., deviations <1.5).

(1) Adiponectin (Figure 11 and Figure 12)

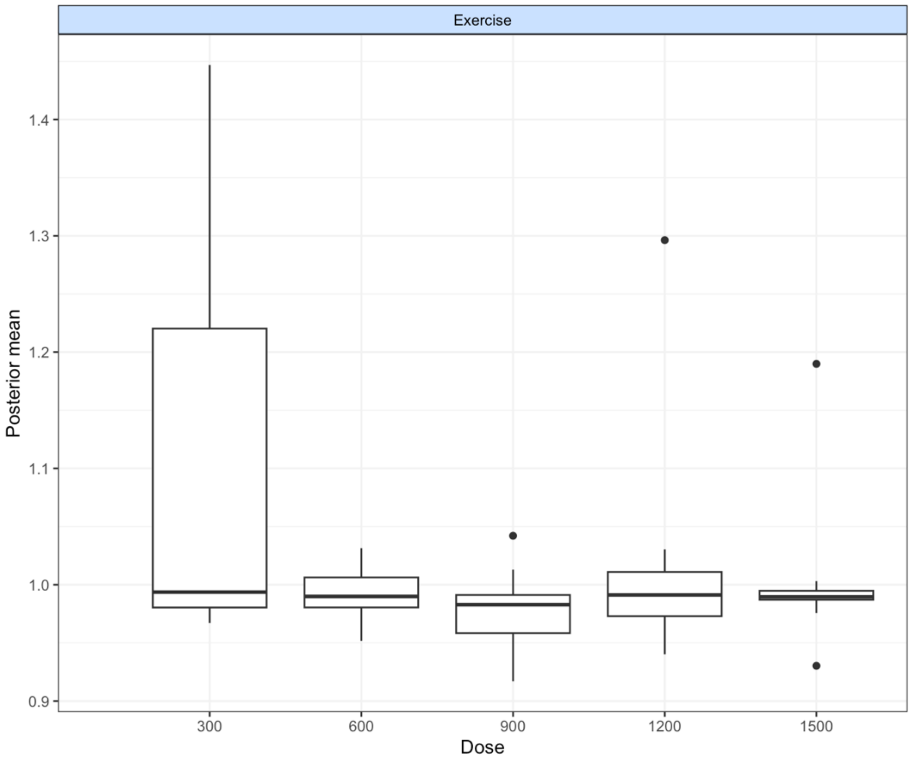

Figure 11 Deviance plot at overall exercise

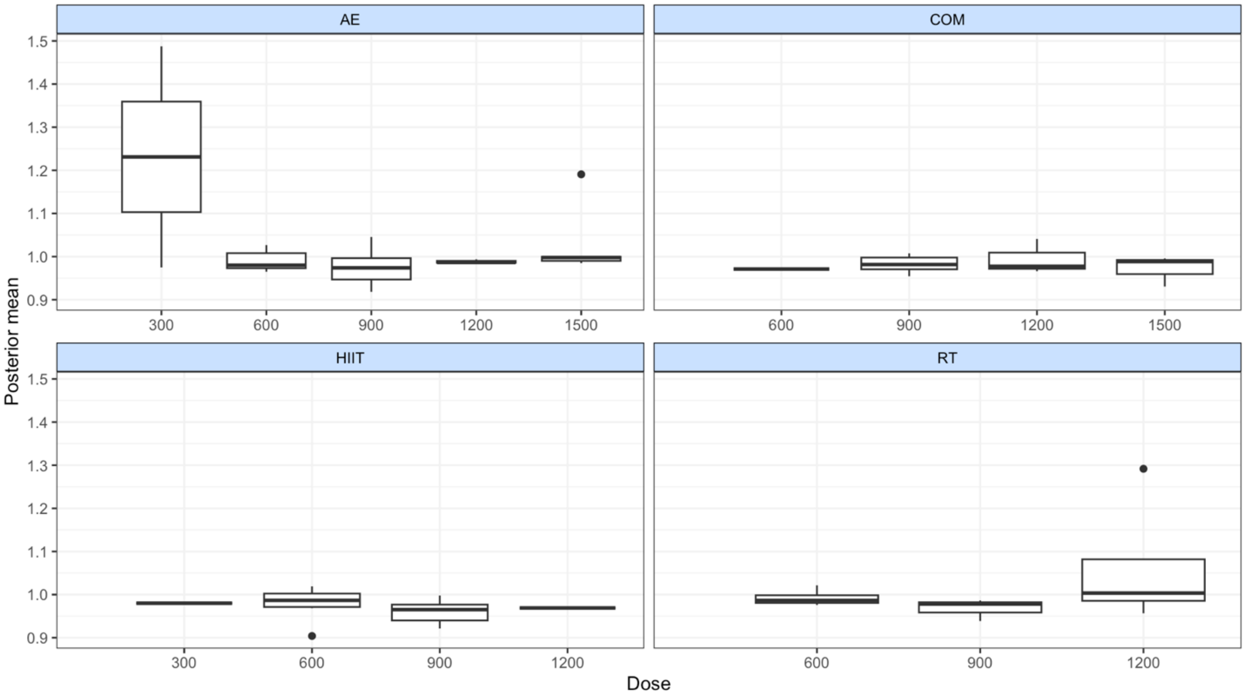

Figure 12 Deviance plots at treatment-level. AE: Aerobic exercise; RT: Resistance training; COM: Aerobic combined with resistance training; HIIT: High- intensity interval training

(2) Leptin (Figure 13 and Figure 14)

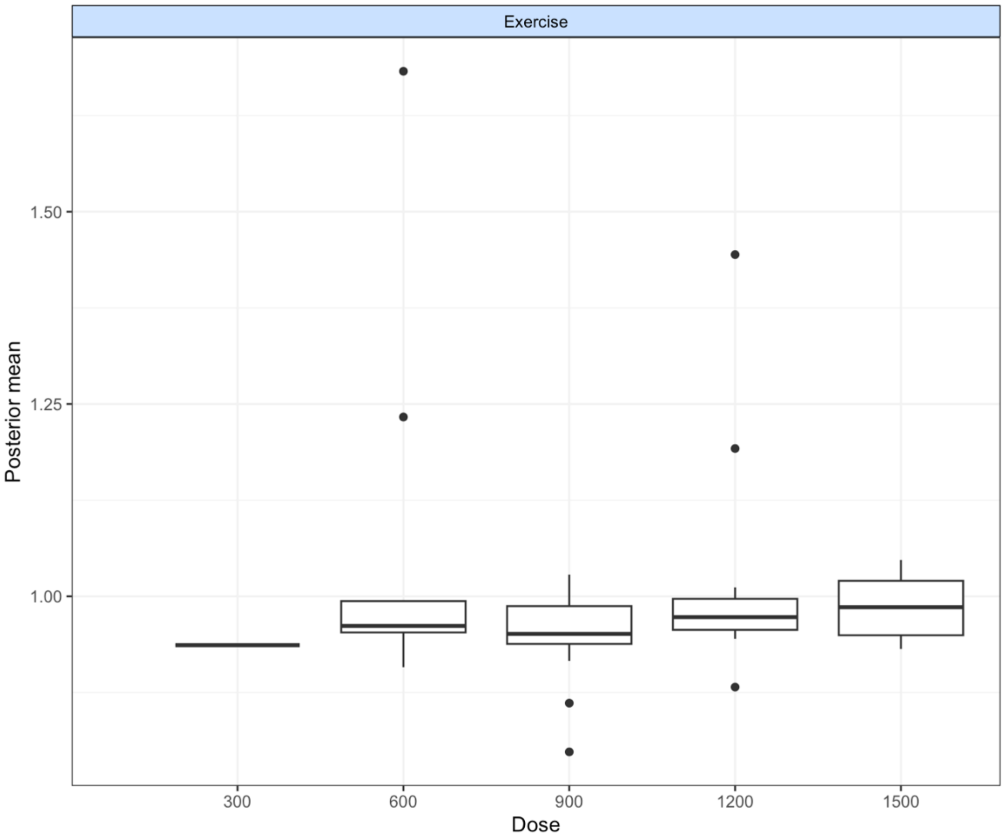

Figure 13 Deviance plot at overall exercise

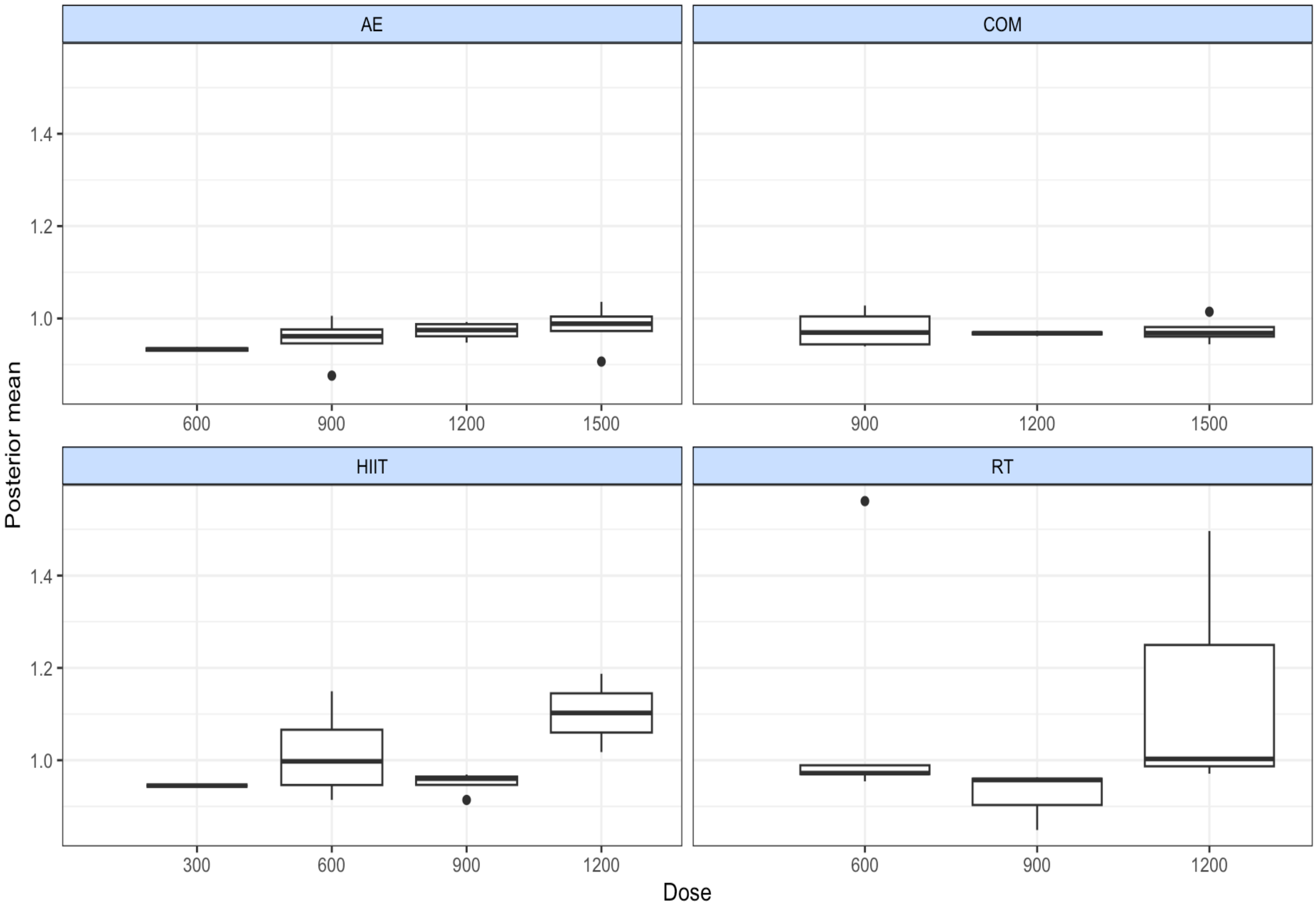

Figure 14 Deviance plots at treatment-level. AE: Aerobic exercise; RT: Resistance training; COM: Aerobic

combined with resistance training; HIIT: High- intensity interval training

Appendix 15 Dose-response relationships

1 The dose-response relationship between exercise dose and circulating levels of adiponectin and leptin

(1) Adiponectin (Figure 15 and Figure 16)

A

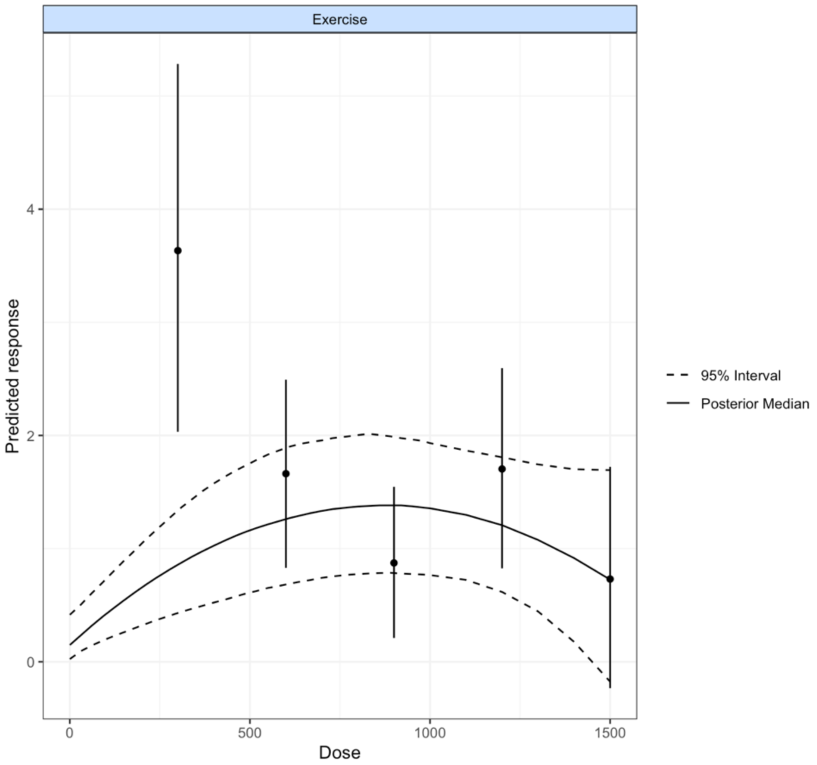

B

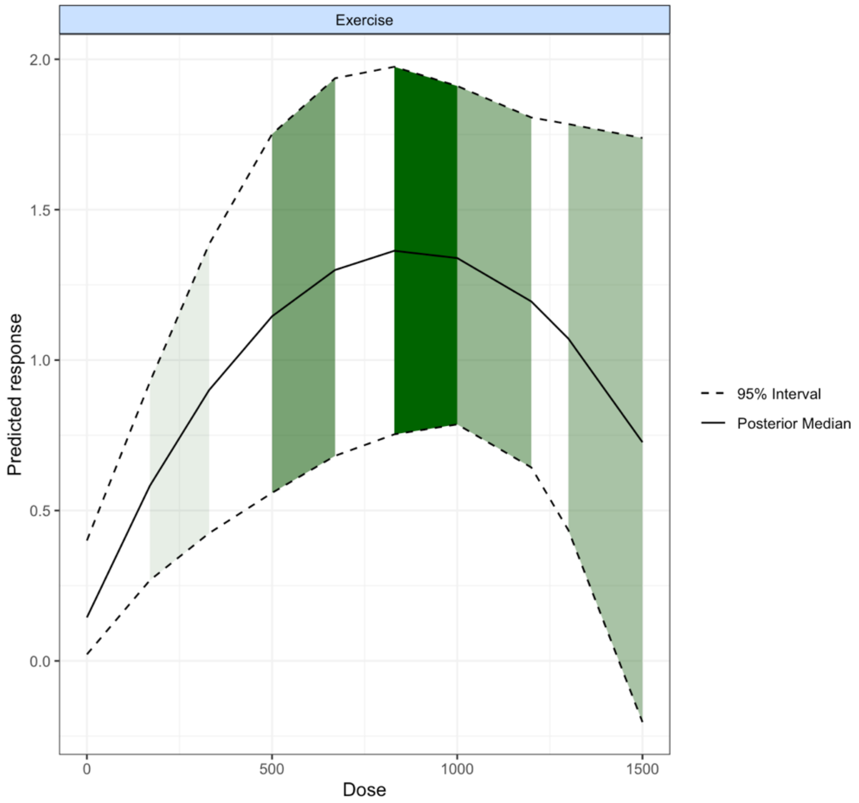

Figure 15 Dose-response relationship between exercise dose and Adiponectin. Figure 15A represents the dose-response + node-split, and the shaded area in Figure 15B represents the original study dataset; the darker the color, the larger the amount of data.

A

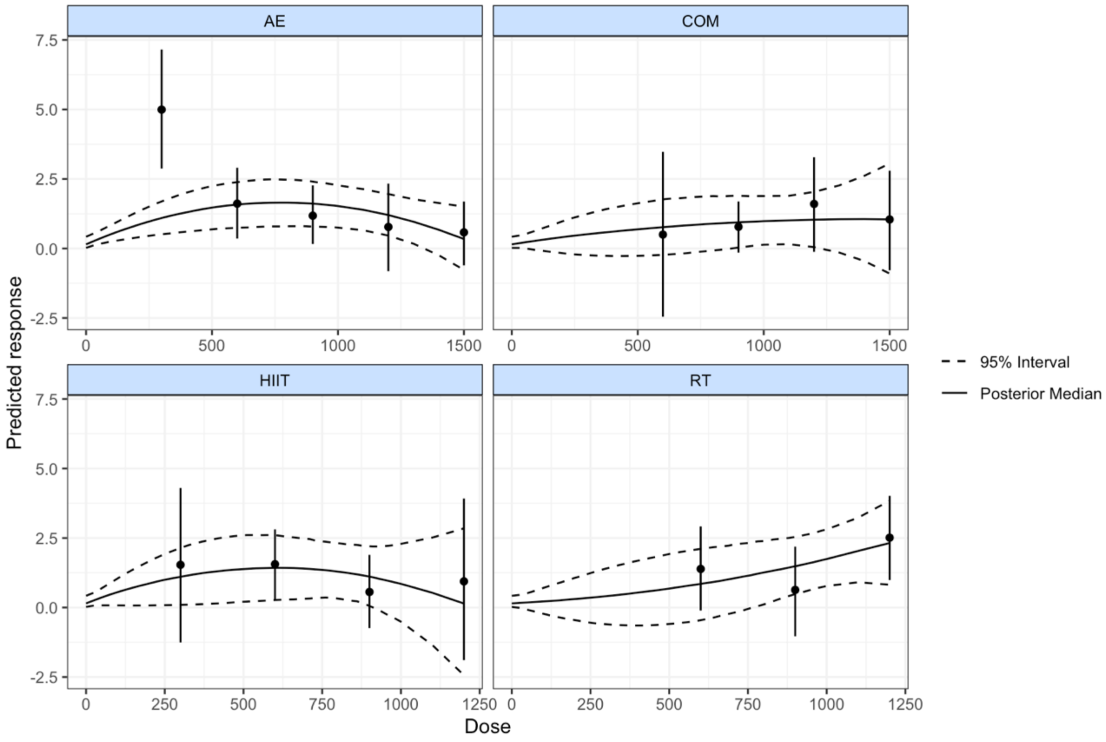

B

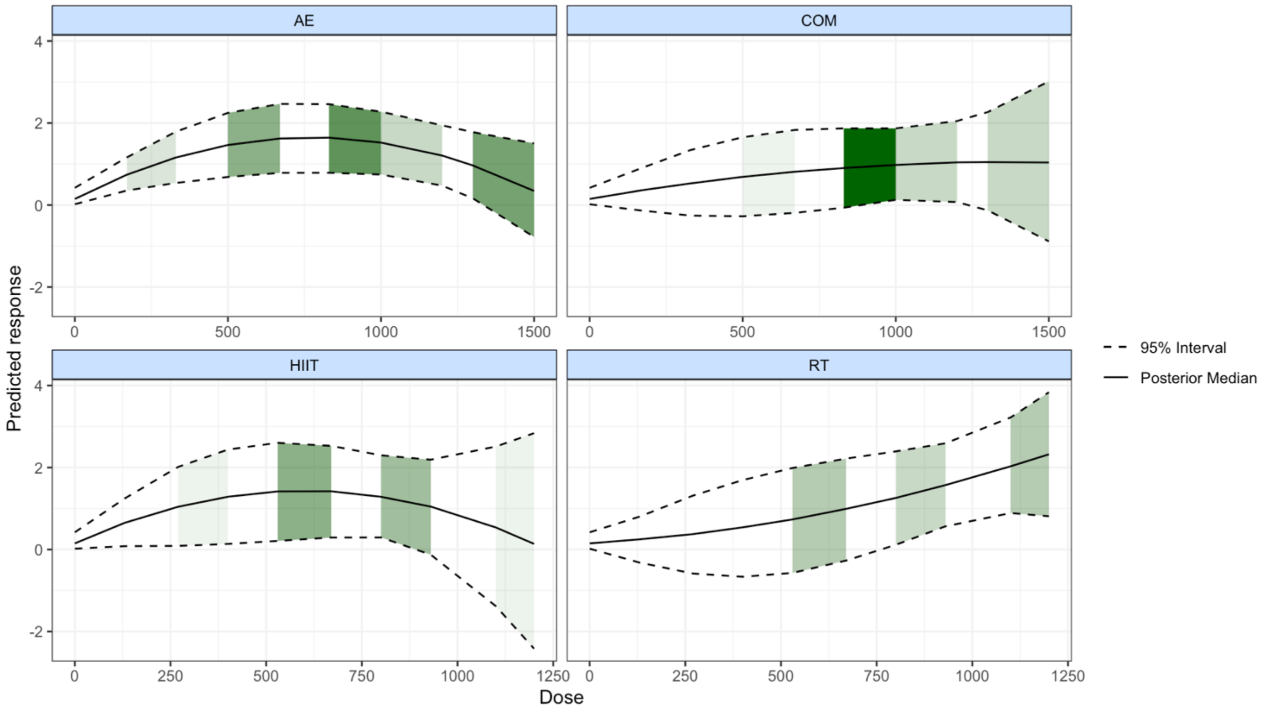

Figure 16 Dose-response relationship at agent level. Figure 16A represents the dose-response + node-split, and the shaded area in Figure 16B represents the original study dataset, the darker the color, the larger the amount of data. AE: Aerobic exercise; RT: Resistance training; COM: Aerobic combined with resistance training; HIIT: High- intensity interval training

(2) Leptin (Figure 17 and Figure 18)

A

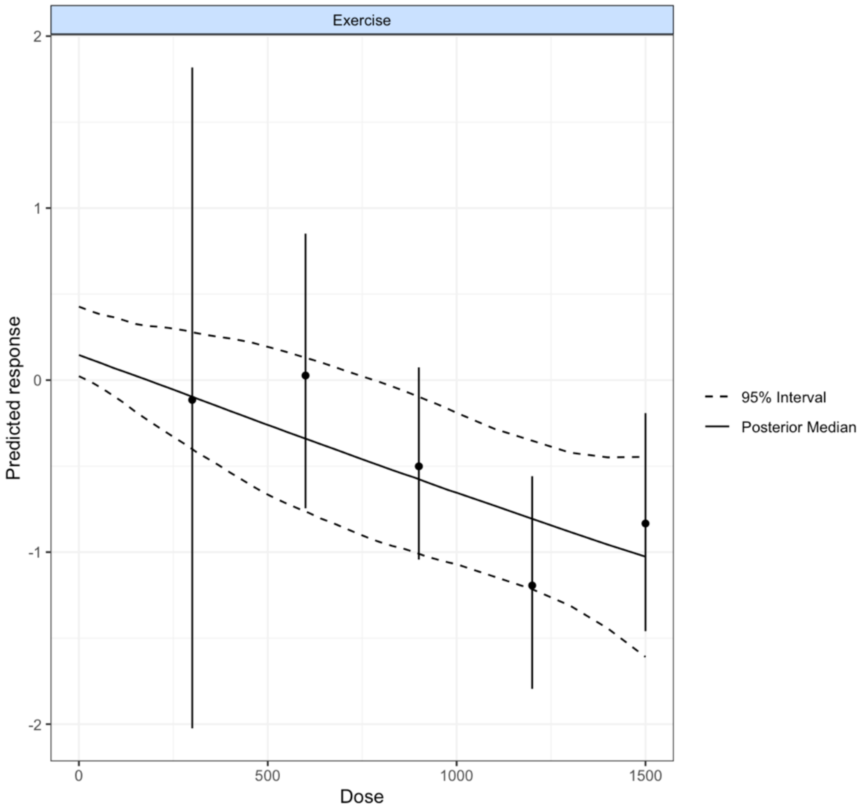

B

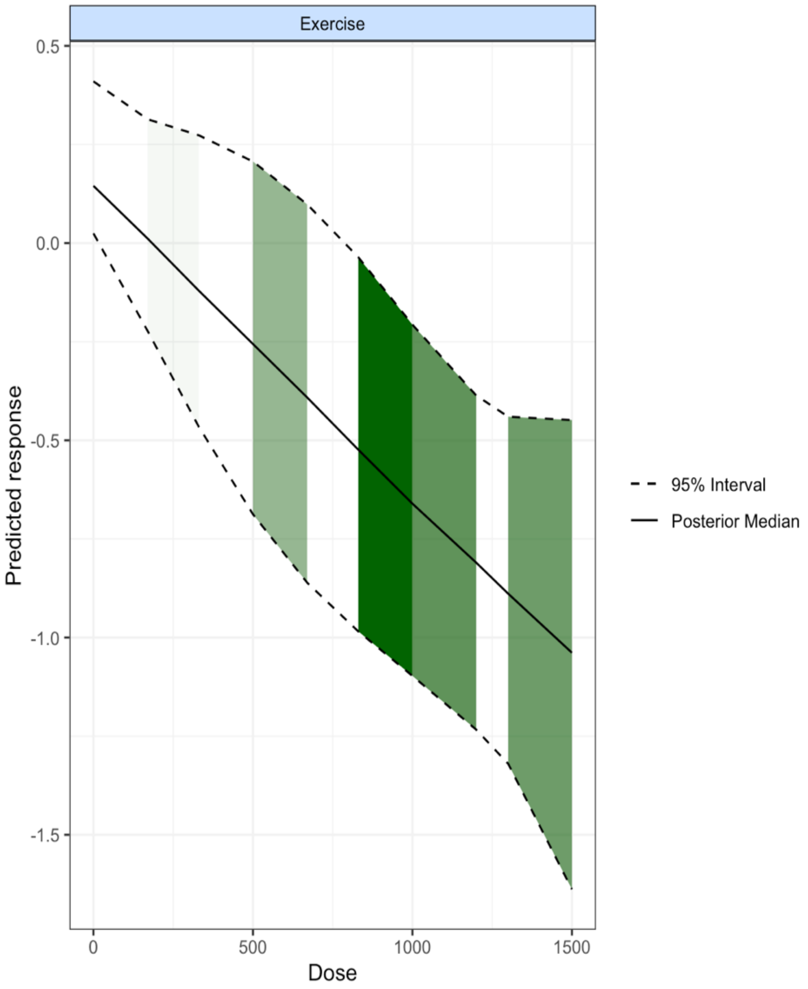

Figure 17 Dose-response relationship between exercise dose and Leptin. Figure 17A represents the dose-response + node-split, and the shaded area in Figure 17B represents the original study dataset; the darker the color, the larger the amount of data.

A

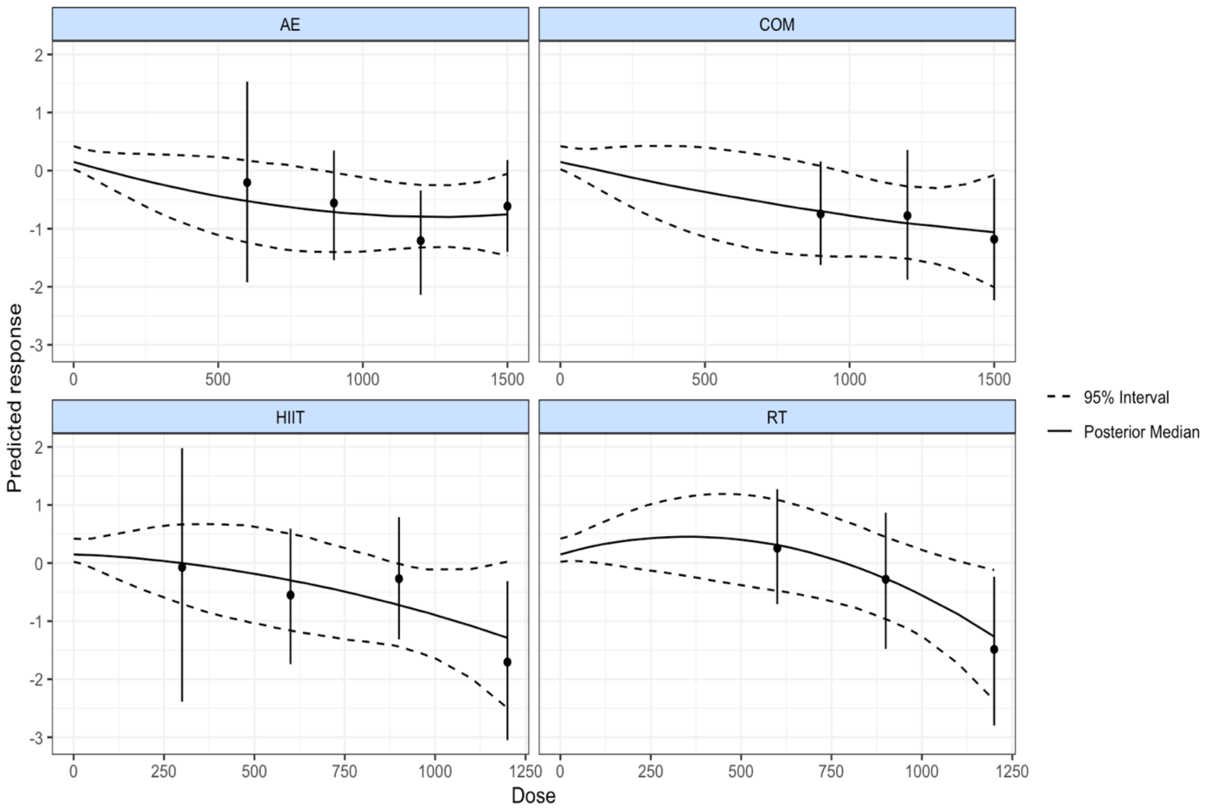

B

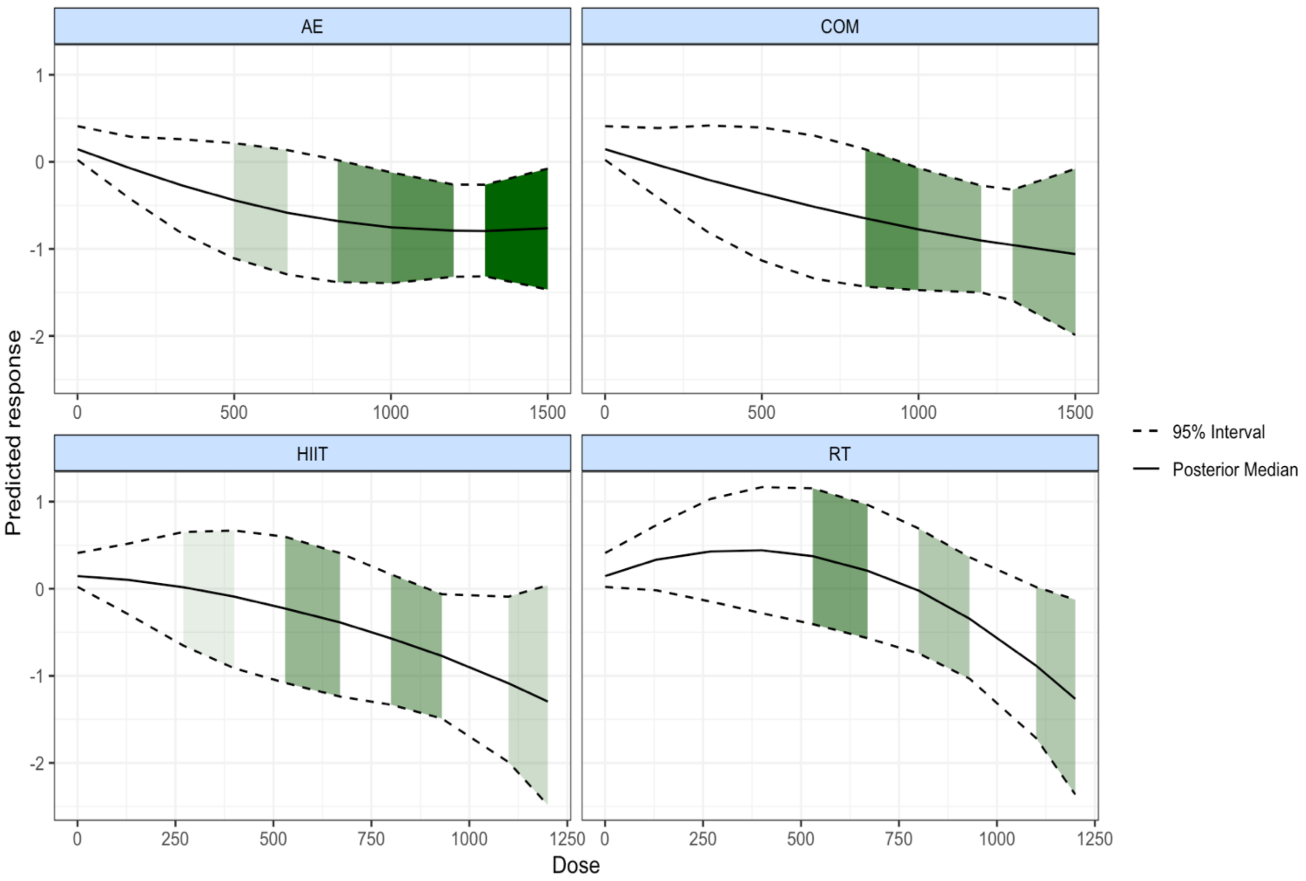

Figure 18 Dose-response relationship at agent level. Figure 18A represents the dose-response + node-split, and the shaded area in Figure 18B represents the original study dataset, the darker the color, the larger the amount of data. AE: Aerobic exercise; RT: Resistance training; COM: Aerobic combined with resistance training;

HIIT: High- intensity interval training

2 Ranking of effectiveness of different exercise

Figure 19 and Figure 20, Table 7 and Table 8 shows the probability of each intervention to be ranked from best to worst (estimated after up to 10000 iterations).

(1) Adiponectin (Figure 19 and Table 7)

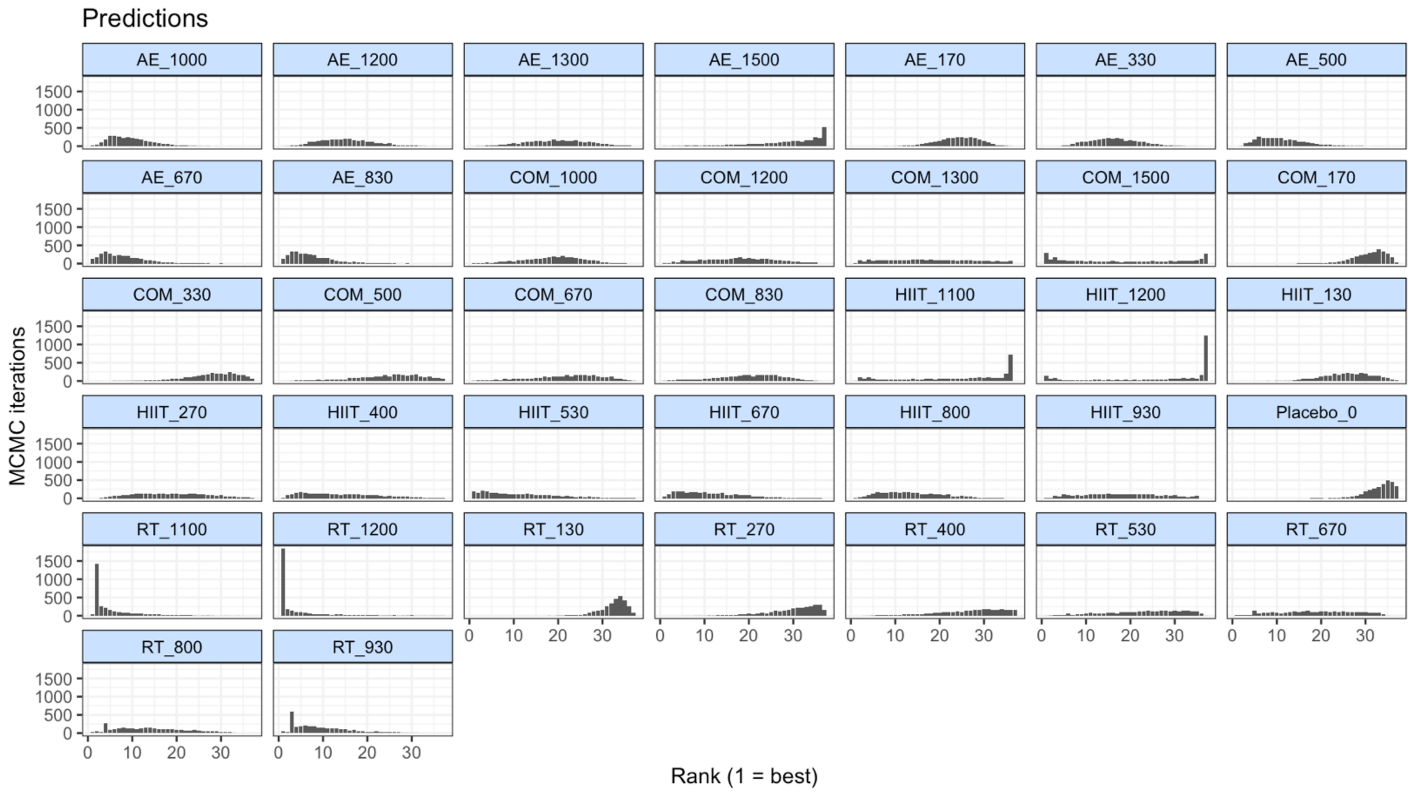

Figure 19 Effectiveness ranking by different exercise. The number that follows the exercise intervention indicates the dose of exercise (METs-min/week). AE: Aerobic exercise; RT: Resistance training; COM: Aerobic combined with resistance training; HIIT: High- intensity interval training

Table 7 Predictions ranking (from best to worst)

Treatment	Mean	Median	2.5%	97.50%
RT_1200	2.54	1	1.00	15
RT_1100	3.92	2	2.00	15
AE_830	7.38	6	1.00	19
AE_670	8.10	7	1.98	20
RT_930	8.48	7	3.00	23
AE_1000	9.69	9	3.00	21
AE_500	11.45	10	4.00	24
HIIT_670	11.71	11	2.00	28
HIIT_530	11.79	10	1.00	30
RT_800	13.92	13	3.00	30
HIIT_800	13.96	13	4.00	28
HIIT_400	14.51	13	3.00	33
AE_1200	15.14	15	5.00	28
AE_330	16.64	16	7.00	28
COM_1300	17.95	17	3.00	35
COM_1500	18.00	17	1.00	37
COM_1200	18.14	18	5.00	33
HIIT_930	18.25	18	4.00	34
COM_1000	18.86	19	6.00	30
HIIT_270	19.08	19	6.00	34
RT_670	19.19	19	5.00	33
AE_1300	19.64	19	7.00	33
COM_830	20.10	21	5.00	32
COM_670	22.01	23	5.00	35
RT_530	23.89	25	6.00	35
AE_170	24.16	24 1	4.00	33
HIIT_1100	24.48	28	3.00	36
COM_500	24.89	26	8.00	37
HIIT_130	26.04	26 1	6.00	35
HIIT_1200	27.27	34	1.00	37
RT_400	27.65	29 1	0.98	37
COM_330	28.01	29 1	5.00	36
AE_1500	28.69	30 1	0.00	37
RT_270	30.71	32 1	7.00	37
COM_170	30.86	31 2	3.00	36
RT_130	32.69	33 2	5.00	37
Placebo_0	33.19	34 2	6.00	37

(2) Leptin (Figure 20 and Table 8)

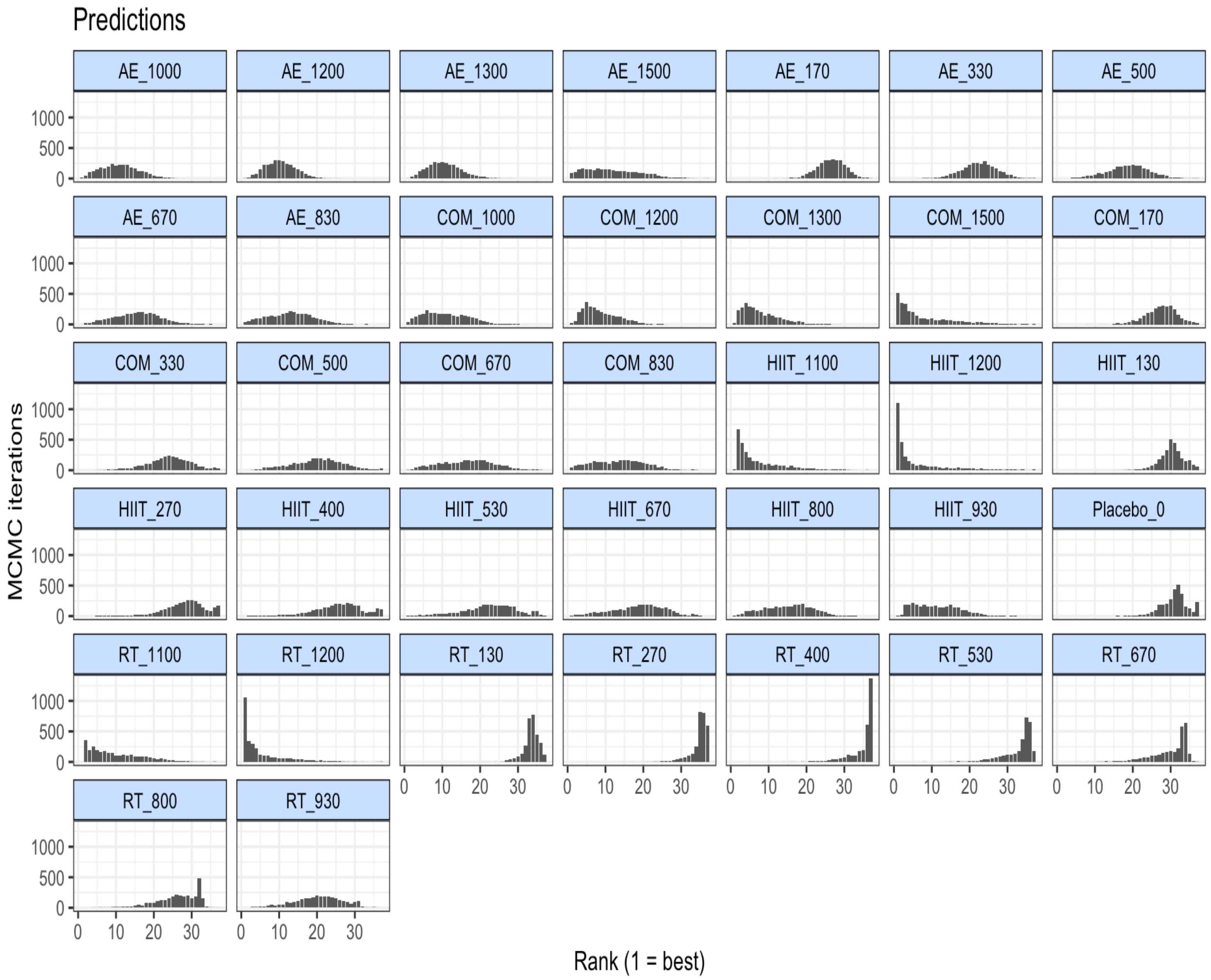

Figure 20 Effectiveness ranking by different exercise. The number that follows the exercise intervention indicates the dose of exercise (METs-min/week). AE: Aerobic exercise; RT: Resistance training; COM: Aerobic combined with resistance training; HIIT: High- intensity interval training

Table 8 Predictions ranking (from best to worst)

Treatment	Mean	Median	2.5%	97.50%
RT_1200	5.73	3	1.00	24
HIIT_1200	6.07	2	1.00	28
HIIT_1100	7.40	5	2.00	24
COM_1300	7.89	7	2.00	20
COM_1500	7.96	5	1.00	27
COM_1200	8.65	8	3.00	19
RT_1100	10.23	9	2.00	26
AE_1200	10.39	10	3.00	18
AE_1300	10.52	10	3.00	20
COM_1000	10.79	10	2.00	23
AE_1000	11.07	11	3.00	21
HIIT_930	11.13	11	3.00	23
AE_1500	11.81	11	1.00	27
AE_830	12.96	13	2.00	24
COM_830	13.51	13	2.00	27
HIIT_800	15.05	15	4.00	27
AE_670	15.71	16	4.00	27
COM_670	16.63	17	3.98	30
HIIT_670	18.80	20	4.00	32.03
AE_500	19.06	19	8.00	30
RT_930	20.12	20	6.00	31
COM_500	20.20	21	6.00	34
HIIT_530	22.60	23	7.00	35
AE_330	22.74	23	13.00	32
COM_330	23.98	24	11.00	36
HIIT_400	25.81	26	11.00	37
RT_800	26.11	27	13.00	33
AE_170	26.70	27	19.00	34
COM_170	27.57	28	19.00	36
HIIT_270	28.49	29	17.00	37
RT_670	29.98	32	17.00	35
HIIT_130	30.36	30	24.00	36
Placebo_0	31.21	31	24.00	37
RT_530	32.81	35	20.98	37
RT_130	33.53	34	28.00	37
RT_400	34.69	36	24.00	37
RT_270	34.75	35	27.00	37

Reference:

1. Armamento-Villareal, R., Aguirre, L., Waters, D. L., Napoli, N., Qualls, C., & Villareal, D. T. (2020). Effect of aerobic or resistance exercise, or both, on bone mineral density and bone metabolism in obese older adults while dieting: A randomized controlled trial. *Journal of Bone and Mineral Research*, *35*(3), 430–439. https://doi.org/10.1002/jbmr.3905
2. Abbenhardt, C., McTiernan, A., Alfano, C. M., Wener, M. H., Campbell, K. L., Duggan, C., Foster-Schubert, K. E., Kong, A., Toriola, A. T., Potter, J. D., Mason, C., Xiao, L., Blackburn, G. L., Bain, C., & Ulrich, C. M. (2013). Effects of individual and combined dietary weight loss and exercise interventions in postmenopausal women on adiponectin and leptin levels. *Journal of Internal Medicine*, *274*(2), 163–175. https://doi.org/10.1111/joim.12062
3. Aghajani, M., Rahmati-Ahmadabad, S., Zamani, F., Ghanbari, B., & Azarbayjani, M.-A. (2022). The effects of high-intensity interval training and orlistat on selected adipokines and cytokines in obese women. *German Journal of Exercise and Sport Research*, *52*(1), 87–96. https://doi.org/10.1007/s12662-021-00749-z
4. Akbarpour, M. (2013). The effect of aerobic training on serum adiponectin and leptin levels and inflammatory markers of coronary heart disease in obese men. *Biology of Sport*, *30*(1), 21–27. https://doi.org/10.5604/20831862.1029817
5. Almenning, I., Rieber-Mohn, A., Lundgren, K. M., Shetelig Løvvik, T., Garnæs, K. K., & Moholdt, T. (2015). Effects of high intensity interval training and strength training on metabolic, cardiovascular and hormonal outcomes in women with polycystic ovary syndrome: A pilot study. *PLOS One*, *10*(9), e0138793. https://doi.org/10.1371/journal.pone.0138793
6. Bagheri, R., Rashidlamir, A., Ashtary-Larky, D., Wong, A., Grubbs, B., Motevalli, M. S., Baker, J. S., Laher, I., & Zouhal, H. (2020). Effects of green tea extract supplementation and endurance training on irisin, pro-inflammatory cytokines, and adiponectin concentrations in overweight middle-aged men. *European Journal of Applied Physiology*, *120*(4), 915–923. https://doi.org/10.1007/s00421-020-04332-6
7. Bagheri, N., Bagheri, R., Mesinovic, J., Ghobadi, H., Scott, D., Kargarfard, M., & Dutheil, F. (2025). Effects of resistance training on muscular adaptations and inflammatory markers in overweight and obese men. *Medicine & Science in Sports & Exercise*, *57*(3), 600–612. https://doi.org/10.1249/MSS.0000000000003592
8. Baitul, M. S., Susanto, H., Kushartanti, W., Soegiyanto, & Rahayu, S. (2017). Beneficial health effect of aquarobics (role of adiponectin on women with obesity). *IOP Conference Series: Materials Science and Engineering*, *180*, 12170. https://doi.org/10.1088/1757-899X/180/1/012170
9. Balducci, S., Zanuso, S., Nicolucci, A., Fernando, F., Cavallo, S., Cardelli, P., Fallucca, S., Alessi, E., Letizia, C., Jimenez, A., Fallucca, F., & Pugliese, G. (2010). Anti-inflammatory effect of exercise training in subjects with type 2 diabetes and the metabolic syndrome is dependent on exercise modalities and independent of weight loss. *Nutrition, Metabolism and Cardiovascular Diseases*, *20*(8), 608–617. https://doi.org/10.1016/j.numecd.2009.04.015
10. Bharath, L. P., Choi, W. W., Cho, J., Skobodzinski, A. A., Wong, A., Sweeney, T. E., & Park, S.-Y. (2018). Combined resistance and aerobic exercise training reduces insulin resistance and central adiposity in adolescent girls who are obese: Randomized clinical trial. *European Journal of Applied Physiology*,

118(8), 1653–1660. https://doi.org/10.1007/s00421-018-3898-8

11. Bonfante, I. L. P., Monfort-Pires, M., Duft, R. G., Da Silva Mateus, K. C., De Lima Júnior, J. C., Dos Santos Trombeta, J. C., Finardi, E. A. R., Brunelli, D. T., Morari, J., De Lima, J. A. B., Bellotto, M. L., De Araújo, T. M. F., Ramos, C. D., Chacon-Mikahil, M. P. T., Velloso, L. A., & Cavaglieri, C. R. (2022). Combined training increases thermogenic fat activity in patients with overweight and type 2 diabetes. *International Journal of Obesity*, *46*(6), 1145–1154. https://doi.org/10.1038/s41366-022-01086-3
12. Bouchonville, M., Armamento-Villareal, R., Shah, K., Napoli, N., Sinacore, D. R., Qualls, C., & Villareal, D. T. (2014). Weight loss, exercise or both and cardiometabolic risk factors in obese older adults: Results of a randomized controlled trial. *International Journal of Obesity*, *38*(3), 423–431. https://doi.org/10.1038/ijo.2013.122
13. Da Silveira Campos, R. M., Landi Masquio, D. C., Campos Corgosinho, F., De Lima Sanches, P., De Piano, A., Carnier, J., Leão Da Silva, P., Grotti Clemente, A. P., De Castro Ferreira Vicente, S. E., Oyama, L. M., Da Penha Oller Do Nascimento, C. M., Tock, L., Tufik, S., De Mello, M. T., & Dâmaso, A. R. (2017). Homeostasis model assessment-adiponectin: The role of different types of physical exercise in obese adolescents. *Journal of Sports Medicine and Physical Fitness*, *57*(6). https://doi.org/10.23736/S0022-4707.16.06235-6
14. Chae, H.-W., Kwon, Y.-N., Rhie, Y.-J., Kim, H.-S., Kim, Y.-S., Paik, I.-Y., Suh, S.-H., & Kim, D.-H. (2010). Effects of a structured exercise program on insulin resistance, inflammatory markers and physical fitness in obese korean children. *Journal of Pediatric Endocrinology and Metabolism*, *23*(10). https://doi.org/10.1515/jpem.2010.168
15. Chuensiri, N., Suksom, D., & Tanaka, H. (2018). Effects of high-intensity intermittent training on vascular function in obese preadolescent boys. *Childhood Obesity*, *14*(1), 41–49. https://doi.org/10.1089/chi.2017.0024
16. Coker, R. H., Williams, R. H., Kortebein, P. M., Sullivan, D. H., & Evans, W. J. (2009). Influence of exercise intensity on abdominal fat and adiponectin in elderly adults. *Metabolic Syndrome and Related Disorders*, *7*(4), 363–368. https://doi.org/10.1089/met.2008.0060
17. Dâmaso, A. R., Campos, R. M. D. S., Caranti, D. A., De Piano, A., Fisberg, M., Foschini, D., Sanches, P. D. L., Tock, L., Lederman, H. M., Tufik, S., & De Mello, M. Tú. (2014). Aerobic plus resistance training was more effective in improving the visceral adiposity, metabolic profile and inflammatory markers than aerobic training in obese adolescents. *Journal of Sports Sciences*, 1–11. https://doi.org/10.1080/02640414.2014.900692
18. El-Kader, S. M. A., Al-Shreef, F. M., & Al-Jiffri, O. H. (2016). Biochemical parameters response to weight loss in patients with non-alcoholic steatohepatitis. *African Health Sciences*, *16*(1), 242. https://doi.org/10.4314/ahs.v16i1.32
19. Ackel-D'Elia, C., Carnier, J., Bueno, C., Campos, R. M., Sanches, P., Clemente, A. P., Tufik, S., De Mello, M., & Dâmaso, A. (2013). Effects of different physical exercises on leptin concentration in obese adolescents. *International Journal of Sports Medicine*, *35*(2), 164–171. https://doi.org/10.1055/s-0033-1345128
20. Fatouros, I. G., Tournis, S., Leontsini, D., Jamurtas, A. Z., Sxina, M., Thomakos, P., Manousaki, M., Douroudos, I., Taxildaris, K., & Mitrakou, A. (2005). Leptin and adiponectin responses in overweight inactive elderly following resistance training and detraining are intensity related. *Journal of Clinical*

Endocrinology and Metabolism, *90*(11), 5970–5977. https://doi.org/10.1210/jc.2005-0261
21. Frank, L. L., Sorensen, B. E., Yasui, Y., Tworoger, S. S., Schwartz, R. S., Ulrich, C. M., Irwin, M. L., Rudolph, R. E., Rajan, K. B., Stanczyk, F., Bowen, D., Weigle, D. S., Potter, J. D., & McTiernan, A. (2005). Effects of exercise on metabolic risk variables in overweight postmenopausal women: A randomized clinical trial. *Obesity Research*, *13*(3), 615–625. https://doi.org/10.1038/oby.2005.66
22. Hara, T., Fujiwara, H., Nakao, H., Mimura, T., Yoshikawa, T., & Fujimoto, S. (2005). Body composition is related to increase in plasma adiponectin levels rather than training in young obese men. *European Journal of Applied Physiology*, *94*(5–6), 520–526. https://doi.org/10.1007/s00421-005-1374-8
23. Horváth, J., Seres, I., Paragh, G., Fülöp, P., & Jenei, Z. (2024). Effect of low- and moderate-intensity aerobic training on body composition cardiorespiratory functions, biochemical risk factors and adipokines in morbid obesity. *Nutrients*, *16*(23), 4251. https://doi.org/10.3390/nu16234251
24. Johannsen, N. M., Swift, D. L., Johnson, W. D., Dixit, V. D., Earnest, C. P., Blair, S. N., & Church, T. S. (2012). Effect of different doses of aerobic exercise on total white blood cell (WBC) and WBC subfraction number in postmenopausal women: Results from DREW. *PLOS One*, *7*(2), e31319. https://doi.org/10.1371/journal.pone.0031319
25. Kadoglou, N. P. E., Iliadis, F., Angelopoulou, N., Perrea, D., Ampatzidis, G., Liapis, C. D., & Alevizos, M. (2007). The anti-inflammatory effects of exercise training in patients with type 2 diabetes mellitus. *European Journal of Cardiovascular Prevention and Rehabilitation*, *14*(6), 837–843. https://doi.org/10.1097/HJR.0b013e3282efaf50
26. Karacabey, K. (2009). The effect of exercise on leptin, insulin, cortisol and lipid profiles in obese children. *Journal of International Medical Research*, *37*(5), 1472–1478. https://doi.org/10.1177/147323000903700523
27. Kelly, A. S., Steinberger, J., Olson, T. P., & Dengel, D. R. (2007). In the absence of weight loss, exercise training does not improve adipokines or oxidative stress in overweight children. *Metabolism*, *56*(7), 1005–1009. https://doi.org/10.1016/j.metabol.2007.03.009
28. Department of Sport Sciences, Kosar University of Bojnord Faculty of Humanities, Iran, Khademosharie, M., Mollanovruzi, A., & Department of Sport Sciences, Kosar University of Bojnord Faculty of Humanities, Iran. (2023). Effects of high-intensity training upon appetite, body mass, aerobic capacity, and metabolic hormones in overweight women. *Endocrinology Research and Practice*, *27*(1), 21–27. https://doi.org/10.5152/erp.2023.22047
29. Kim, S.-W., Jung, W.-S., Park, W., & Park, H.-Y. (2019). Twelve weeks of combined resistance and aerobic exercise improves cardiometabolic biomarkers and enhances red blood cell hemorheological function in obese older men: A randomized controlled trial. *International Journal of Environmental Research and Public Health*, *16*(24), 5020. https://doi.org/10.3390/ijerph16245020
30. Kim, Y., Nam, J. S., Yeo, D., Kim, K. R., Suh, S., & Ahn, C. W. (2015). The effects of aerobic exercise training on serum osteocalcin, adipocytokines and insulin resistance on obese young males. *Clinical Endocrinology*, *82*(5), 686–694. https://doi.org/10.1111/cen.12601
31. Ku, Y., Han, K., Ahn, H., Kwon, H., Koo, B., Kim, H., & Min, K. (2010). Resistance exercise did not alter intramuscular adipose tissue but reduced retinol-binding protein-4 concentration in individuals with type 2 diabetes mellitus. *Journal of International Medical Research*, *38*(3), 782–791. https://doi.org/10.1177/147323001003800305

32. Li, C., Lyu, S., & Zhang, J. (2022). Effects of aerobic exercise on the serum leptin level and heart rate variability in the obese girl children. *Computational Intelligence and Neuroscience*, *2022*, 1–6. https://doi.org/10.1155/2022/2298994
33. Lopes, W. A., Leite, N., Da Silva, L. R., Brunelli, D. T., Gáspari, A. F., Radominski, R. B., Chacon-Mikahil, M. P. T., & Cavaglieri, C. R. (2016). Effects of 12 weeks of combined training without caloric restriction on inflammatory markers in overweight girls. *Journal of Sports Sciences*, *34*(20), 1902–1912. https://doi.org/10.1080/02640414.2016.1142107
34. Marcell, T. J., McAuley, K. A., Traustadóttir, T., & Reaven, P. D. (2005). Exercise training is not associated with improved levels of C-reactive protein or adiponectin. *Metabolism*, *54*(4), 533–541. https://doi.org/10.1016/j.metabol.2004.11.008
35. Miller, G. D., Nicklas, B. J., Davis, C. C., Ambrosius, W. T., Loeser, R. F., & Messier, S. P. (2004). Is serum leptin related to physical function and is it modifiable through weight loss and exercise in older adults with knee osteoarthritis? *International Journal of Obesity*, *28*(11), 1383–1390. https://doi.org/10.1038/sj.ijo.0802737
36. Moghadasi, M., Mohebbi, H., Rahmani-Nia, F., Hassan-Nia, S., Noroozi, H., & Pirooznia, N. (2012). High-intensity endurance training improves adiponectin mRNA and plasma concentrations. *European Journal of Applied Physiology*, *112*(4), 1207–1214. https://doi.org/10.1007/s00421-011-2073-2
37. Murphy, E. C.-S., Carson, L., Neal, W., Baylis, C., Donley, D., & Yeater, R. (2009). Effects of an exercise intervention using dance dance revolution on endothelial function and other risk factors in overweight children. *International Journal of Pediatric Obesity: IJPO: An Official Journal of the International Association for the Study of Obesity*, *4*(4), 205–214. https://doi.org/10.3109/17477160902846187
38. Nunes, P. R. P., Martins, F. M., Souza, A. P., Carneiro, M. A. S., Orsatti, C. L., Michelin, M. A., Murta, E. F. C., De Oliveira, E. P., & Orsatti, F. L. (2019). Effect of high-intensity interval training on body composition and inflammatory markers in obese postmenopausal women: A randomized controlled trial. *Menopause*, *26*(3), 256–264. https://doi.org/10.1097/GME.0000000000001207
39. Oh, S., So, R., Shida, T., Matsuo, T., Kim, B., Akiyama, K., Isobe, T., Okamoto, Y., Tanaka, K., & Shoda, J. (2017). High-intensity aerobic exercise improves both hepatic fat content and stiffness in sedentary obese men with nonalcoholic fatty liver disease. *Scientific Reports*, *7*(1), 43029. https://doi.org/10.1038/srep43029
40. Park, J.-H., Miyashita, M., Kwon, Y.-C., Park, H.-T., Kim, E.-H., Park, J.-K., Park, K.-B., Yoon, S.-R., Chung, J.-W., Nakamura, Y., & Park, S.-K. (2012). A 12-week after-school physical activity programme improves endothelial cell function in overweight and obese children: A randomised controlled study. *BMC Pediatrics*, *12*(1), 111. https://doi.org/10.1186/1471-2431-12-111
41. Phillips, M. D., Patrizi, R. M., Cheek, D. J., Wooten, J. S., Barbee, J. J., & Mitchell, J. B. (2012). Resistance training reduces subclinical inflammation in obese, postmenopausal women. *Medicine & Science in Sports & Exercise*, *44*(11), 2099–2110. https://doi.org/10.1249/MSS.0b013e3182644984
42. Racil, G., Ben Ounis, O., Hammouda, O., Kallel, A., Zouhal, H., Chamari, K., & Amri, M. (2013). Effects of high vs. Moderate exercise intensity during interval training on lipids and adiponectin levels in obese young females. *European Journal of Applied Physiology*, *113*(10), 2531–2540. https://doi.org/10.1007/s00421-013-2689-5
43. Racil, G., Zouhal, H., Elmontassar, W., Abderrahmane, A. B., De Sousa, M. V., Chamari, K., Amri, M., &

Coquart, J. B. (2016). Plyometric exercise combined with high-intensity interval training improves metabolic abnormalities in young obese females more so than interval training alone. *Applied Physiology, Nutrition and Metabolism*, *41*(1), 103–109. https://doi.org/10.1139/apnm-2015-0384

44. Rajabi, A., Khajehlandi, M., Siahkuhian, M., Akbarnejad, A., Khoramipour, K., & Suzuki, K. (2022). Effect of 8 weeks aerobic training and saffron supplementation on inflammation and metabolism in middle-aged obese women with type 2 diabetes mellitus. *Sports*, *10*(11), 167. https://doi.org/10.3390/sports10110167
45. Rezaeeshirazi, R. (2022). Aerobic versus resistance training: Leptin and metabolic parameters improvement in type 2 diabetes obese men. *Research Quarterly for Exercise and Sport*, *93*(3), 537–547. https://doi.org/10.1080/02701367.2021.1875111
46. Salus, M., Tillmann, V., Remmel, L., Unt, E., Mäestu, E., Parm, Ü., Mägi, A., Tali, M., & Jürimäe, J. (2022). Effect of sprint interval training on cardiometabolic biomarkers and adipokine levels in adolescent boys with obesity. *International Journal of Environmental Research and Public Health*, *19*(19), 12672. https://doi.org/10.3390/ijerph191912672
47. Sasimontonkul, S., & Sirivarasai, J. (2024). The 40-min HIIT acutely induced bone formation which was likely through the increases in muscle derived interleukin 6 and adiponectin activation: The 16 weeks of HIIT intervention, longitudinal randomized controlled trial. *Bone*, *184*, 117105. https://doi.org/10.1016/j.bone.2024.117105
48. Şengün, N., Pala, R., Çınar, V., Akbulut, T., Larion, A., Padulo, J., Russo, L., & Migliaccio, G. M. (2024). Alterations in biomarkers associated with cardiovascular health and obesity with short-term lifestyle changes in overweight women: The role of exercise and diet. *Medicina*, *60*(12), 2019. https://doi.org/10.3390/medicina60122019
49. Shokri, E., Heidarianpour, A., & Razavi, Z. (2021). Positive effect of combined exercise on adipokines levels and pubertal signs in overweight and obese girls with central precocious puberty. *Lipids in Health and Disease*, *20*(1), 152. https://doi.org/10.1186/s12944-021-01588-5
50. Silva, F. M., Duarte-Mendes, P., Ferreira, J. P., Carvalho, E., Monteiro, D., Massart, A., Farinha, C., Soares, C. M., & Teixeira, A. M. (2024). Changes in metabolic and inflammatory markers after a combined exercise program in workers: A randomized controlled trial. *Medicine & Science in Sports & Exercise*, *56*(11), 2156–2172. https://doi.org/10.1249/MSS.0000000000003510
51. Soltani, N., Marandi, S. M., Hovsepian, V., Kazemi, M., & Esmaeil, N. (2023). Resistance exercise training augments the immunomodulatory adaptations to aerobic high-intensity interval training. *European Journal of Sport Science*, *23*(11), 2264–2273. https://doi.org/10.1080/17461391.2023.2222703
52. Suder, A., Makiel, K., Targosz, A., Kosowski, P., & Malina, R. M. (2024). Effects of exercise and dietary interventions on asprosin, leptin, and lipid metabolism in males with abdominal obesity, a randomized controlled trial. *Scientific Reports*, *14*(1), 28109. https://doi.org/10.1038/s41598-024-79853-7
53. Tan, A., Dunseath, G., Thomas, R. L., Prior, S. L., Bracken, R. M., & Churm, R. (2025). Effect of home-based exercise with or without a Mediterranean-style diet on adiposity markers in postmenopausal women: A randomized-control trial. *Physiological Reports*, *13*(3), e70239. https://doi.org/10.14814/phy2.70239
54. Tjønna, A. E., Lee, S. J., Rognmo, Ø., Stølen, T. O., Bye, A., Haram, P. M., Loennechen, J. P., Al-Share, Q. Y., Skogvoll, E., Slørdahl, S. A., Kemi, O. J., Najjar, S. M., & Wisløff, U. (2008). Aerobic interval

training versus continuous moderate exercise as a treatment for the metabolic syndrome: A pilot study. *Circulation*, *118*(4), 346–354. https://doi.org/10.1161/CIRCULATIONAHA.108.772822

55. Trapp, E. G., Chisholm, D. J., Freund, J., & Boutcher, S. H. (2008). The effects of high-intensity intermittent exercise training on fat loss and fasting insulin levels of young women. *International Journal of Obesity*, *32*(4), 684–691. https://doi.org/10.1038/sj.ijo.0803781
56. Vella, C. A., Taylor, K., & Drummer, D. (2017). High-intensity interval and moderate-intensity continuous training elicit similar enjoyment and adherence levels in overweight and obese adults. *European Journal of Sport Science*, *17*(9), 1203–1211. https://doi.org/10.1080/17461391.2017.1359679
57. Venojärvi, M., Wasenius, N., Manderoos, S., Heinonen, O. J., Hernelahti, M., Lindholm, H., Surakka, J., Lindström, J., Aunola, S., Atalay, M., & Eriksson, J. G. (2013). Nordic walking decreased circulating chemerin and leptin concentrations in middle-aged men with impaired glucose regulation. *Annals of Medicine*, *45*(2), 162–170. https://doi.org/10.3109/07853890.2012.727020
58. Wong, A., Sanchez-Gonzalez, M. A., Son, W.-M., Kwak, Y.-S., & Park, S.-Y. (2018). The effects of a 12-week combined exercise training program on arterial stiffness, vasoactive substances, inflammatory markers, metabolic profile, and body composition in obese adolescent girls. *Pediatric Exercise Science*, *30*(4), 480–486. https://doi.org/10.1123/pes.2017-0198
59. Wright, J. L., Schenk, J. M., Gulati, R., Beatty, S. J., VanDoren, M., Lin, D. W., Porter, M. P., Morrissey, C., Dash, A., Gore, J. L., Etzioni, R., Plymate, S. R., & Neuhouser, M. L. (2024). The prostate cancer active lifestyle study (PALS): A randomized controlled trial of diet and exercise in overweight and obese men on active surveillance. *Cancer*, *130*(12), 2108–2119. https://doi.org/10.1002/cncr.35241
60. Gedikbasi, A., & Kayapınar, F. Ç. (不详). 35 publications 343 citations see profile. *J Pak Med Assoc*.
61. Zadeh, M. A. M., Afrasyabi, S., & Mohamadi, Z. A. (2022). The effects of exercise training induced calories expenditure on type 2 diabetes related cardio metabolic physiological parameters and adipocytokines. *Journal of Diabetes & Metabolic Disorders*, *21*(2), 1219–1231. https://doi.org/10.1007/s40200-021-00808-0
62. Ter Veer, E., Van Oijen, M. G. H., & Van Laarhoven, H. W. M. (2019). The use of (network) meta-analysis in clinical oncology. *Frontiers in Oncology*, *9*, 822. https://doi.org/10.3389/fonc.2019.00822
63. Wheeler, D. C., Hickson, D. A., & Waller, L. A. (2010). Assessing local model adequacy in bayesian hierarchical models using the partitioned deviance information criterion. *Computational Statistics and Data Analysis*, *54*(6), 1657–1671. https://doi.org/10.1016/j.csda.2010.01.025
64. White, I. R., Barrett, J. K., Jackson, D., & Higgins, J. P. T. (2012). Consistency and inconsistency in network meta-analysis: Model estimation using multivariate meta-regression. *Research Synthesis Methods*, *3*(2), 111–125. https://doi.org/10.1002/jrsm.1045
65. Cipriani, A., Higgins, J. P. T., Geddes, J. R., & Salanti, G. (2013). Conceptual and technical challenges in network meta-analysis. *Annals of Internal Medicine*, *159*(2), 130–137. https://doi.org/10.7326/0003-4819-159-2-201307160-00008
66. Salanti, G., Del Giovane, C., Chaimani, A., Caldwell, D. M., & Higgins, J. P. T. (2014). Evaluating the quality of evidence from a network meta-analysis. *PLOS One*, *9*(7), e99682. https://doi.org/10.1371/journal.pone.0099682
67. Van Valkenhoef, G., Dias, S., Ades, A. E., & Welton, N. J. (2016). Automated generation of node-splitting models for assessment of inconsistency in network meta-analysis. *Research Synthesis Methods*, *7*(1),

80–93. https://doi.org/10.1002/jrsm.1167

68. Mawdsley, D., Bennetts, M., Dias, S., Boucher, M., & Welton, N. (2016). Model-based network meta-analysis: A framework for evidence synthesis of clinical trial data. *CPT: Pharmacometrics & Systems Pharmacology*, *5*(8), 393–401. https://doi.org/10.1002/psp4.12091
69. Dias, S., Sutton, A. J., Ades, A. E., & Welton, N. J. (2013). Evidence synthesis for decision making 2: A generalized linear modeling framework for pairwise and network meta-analysis of randomized controlled trials. *Medical Decision Making*, *33*(5), 607–617. https://doi.org/10.1177/0272989X12458724
